# A Hanks‐type bacterial kinase, PknS, directly phosphorylates the alternative sigma factor EcfK to promote resistance to protist predation

**DOI:** 10.1111/febs.70384

**Published:** 2026-01-08

**Authors:** Lídia dos Passos Lima, Dev Sriranganadane, Daiane Laise da Silva, Natália C. Drebes Dörr, Enzo Breviglieri Sichi Mello, Caio Vinicius dos Reis, Rogério Ferreira Lourenço, José Felipe Teixeira da Silva Santos, Anita Salmazo, Brenno Wendler Miranda, Katlin B. Massirer, Rafael M. Couñago, Cristina E. Alvarez‐Martinez

**Affiliations:** ^1^ Departamento de Genética, Evolução, Microbiologia e Imunologia, Instituto de Biologia Universidade Estadual de Campinas (UNICAMP) Brazil; ^2^ Centro de Química Medicinal (CQMED), Centro de Biologia Molecular e Engenharia Genética (CBMEG) Universidade Estadual de Campinas (UNICAMP) Brazil; ^3^ Structural Genomics Consortium and Division of Chemical Biology and Medicinal Chemistry, UNC Eshelman School of Pharmacy University of North Carolina Chapel Hill NC USA

**Keywords:** amoeba predation, kinase, sigma factor regulation, type VI secretion system, *Xanthomonas*

## Abstract

Serine/threonine (Ser/Thr) kinases of the Hanks‐type family are widespread in bacteria, playing key roles in signal transduction. The transmembrane Ser/Thr kinase PknS (XAC4127) from the phytopathogenic bacterium *Xanthomonas citri* is required for the expression of a type VI secretion system, which confers resistance to predation by the soil amoeba *Dictyostelium discoideum*. PknS exerts its function via activation of the cognate ECF‐type alternative sigma factor EcfK, ultimately triggering the expression of type VI secretion system (T6SS) genes. In this study, we characterize PknS, demonstrating its ability to undergo autophosphorylation both *in vitro* and within *X. citri* cells. Structural analysis of the PknS kinase domain revealed the conservation of the canonical fold characteristic of Hanks‐type kinases. PknS directly phosphorylates EcfK at five Ser/Thr residues located in two distinct regions of the sigma factor: the conserved σ_2_ domain (residue T51) and a nonconserved linker connecting domains σ_2_ and σ_4_ (residues T104, T106, S108, and S110). The conserved residue T51, previously shown to be essential for sigma factor activity in an EcfK homolog, corresponds to a site that directly interacts with the RNA polymerase β′ subunit. Site‐directed mutagenesis analyses further revealed that the conserved residue T106 is also critical for EcfK function. Structural studies indicated that, in addition to T51, phosphorylation at T106 activates EcfK by promoting its interaction with a positively charged pocket within the RNA polymerase β′ subunit. Collectively, our findings describe a previously unknown signal transduction pathway involving a Hanks‐type kinase and a sigma factor, providing new insights into the mechanisms of sigma factor activation via phosphorylation in bacteria.

## Graphical abstract

The *Xanthomonas citri* Hanks‐type kinase PknS autophosphorylates and directly phosphorylates the alternative sigma factor EcfK at five residues. Besides the conserved residue T51 in the σ2 domain, phosphorylation of a residue in the linker between σ2 and σ4 is critical for EcfK activation by promoting its interaction with a positively charged pocket in the RNA polymerase β′ subunit. Activated EcfK induces T6SS genes and bacterial resistance to amoeba predation.

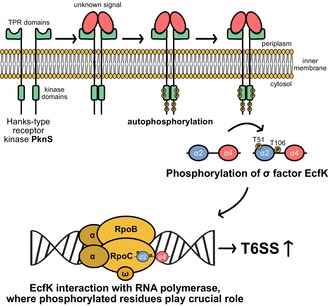

**Abbreviations**
AF3: AlphaFold 3
Cryo‐EM: cryogenic electron microscopy
ECF: extracytoplasmic function
LB: lysogeny broth
LC–MS/MS: liquid chromatography–tandem mass spectrometry
LIC: ligation‐independent cloning
MS: mass spectrometry
RMSD: root mean square deviation
STPK: Serine/threonine and tyrosine protein kinases
T2SS/T3SS/T4SS/T6SS: type II, III, IV, VI secretion system
TB: terrific broth
TCS: two‐component system
TPR: tetratricopeptide repeat domain
WT: wild‐type

## Introduction

Reversible protein phosphorylation mediated by kinases is a major signal transduction mechanism across all domains of life, enabling a rapid cellular response to environmental cues. Typically, a stimulatory signal promotes autophosphorylation of protein kinases, triggering transphosphorylation of specific amino acid residues in target proteins, which then affects a variety of cellular responses, including protein–protein interactions, regulation of transcription or translation, metabolism and other regulatory pathways. In eukaryotes, protein kinases form a large family (Hanks‐type kinases, also known as serine/threonine and tyrosine protein kinases—STPK) that target Ser/Thr or Tyr residues on protein substrates, generating stable phosphate bonds that generally require a cognate phosphatase to reverse the reaction [1]. In bacteria, the identification of diverse protein kinase families has revealed a broader spectrum of phosphorylatable amino acid residues than is typically observed in eukaryotes [2]. Although the His/Asp phosphotransfer reactions mediated by histidine kinases of two‐component systems (TCS) have been considered the canonical phosphorylation‐dependent mechanism for signal transduction in bacteria [3], studies on several bacterial species revealed that Ser/Thr phosphorylation by Hanks‐type protein kinases is widespread and plays key roles in bacterial physiology, including control of virulence, development processes, cell growth and antibiotic persistence [4, 5]. In this sense, recent evidence of TCS regulation by Hanks‐type kinases underscores the presence of intricate phosphorylation‐dependent signal transduction mechanisms in bacteria [5]. In contrast to the high substrate specificity that defines TCS, Hanks‐type Ser/Thr protein kinases (STPKs) usually target multiple substrates simultaneously, and crosstalk with other signaling pathways is common [6]. Bacterial STPKs exhibit a variety of architectures, typically consisting of one or more sensor modules that activate the catalytic domain upon binding to a specific signal [7, 8]. Moreover, many bacterial STPKs function as receptor kinases, characterized by the presence of a transmembrane domain and an extracytoplasmic sensor module, as exemplified by 9 of the 11 STPKs identified in *Mycobacterium tuberculosis* [4].

The *Xanthomonas* genus of Gammaproteobacteria comprises more than 30 species of plant‐associated bacteria, most of them infecting a specific subset of susceptible host plants and, as a group, affect more than 400 economically important crops [9]. Among them, *Xanthomonas citri* pv. *citri* is the causal agent of citrus canker, a disease that results from bacterial colonization of the plant mesophyll and manifests as necrotic lesions in stems, fruits and leaves. *X. citri* is also found colonizing the surface of plant leaves as an epiphyte without causing disease and in association with plant debris in the soil. To adapt to these distinct environments, *X. citri* deploys a variety of secretion systems, which are protein injection nanomachines that deliver protein effectors to the extracellular milieu or directly into prokaryotic or eukaryotic target cells [10]. Among them, the type II and type III secretion systems (T2SS and T3SS) are required for virulence and survival within the plant [10, 11, 12, 13], whereas the *X. citri* type IV secretion system (T4SS) is bactericidal, secreting toxins that eliminate competing bacteria [10, 11, 12]. We described the role of *X. citri*'s type VI secretion system (T6SS) in the resistance to predation by the soil amoeba *Dictyostelium discoideum*, revealing an additional survival strategy in this bacterium, which is also found in a subset of species within the *Xanthomonas* genus [14, 15]. Induction of *X. citri* T6SS gene expression is triggered upon contact with amoeba cells by a signaling pathway that requires the Hanks‐type receptor kinase PknS and the alternative sigma factor EcfK [14]. In our previous study, we identified a phosphomimetic mutation at one conserved threonine residue of EcfK (EcfK^T51E^) that leads to constitutive activation of the sigma factor and induction of the T6SS genes in the absence of amoeba contact. Furthermore, PknS is dispensable for T6SS induction when the phosphomimetic version EcfK^T51E^ is overexpressed, indicating that EcfK is a direct substrate of PknS [14]. EcfK is a member of the extracytoplasmic function sigma factor family (ECF), the most diverse subgroup within the σ^70^ family, which has been mainly associated with signal transduction across the bacterial envelope [16]. This family has been subdivided into 157 phylogenetic groups that display conservation in their genomic context and regulation mechanisms [17]. EcfK belongs to the ECF43 group, in which the co‐occurrence with a gene encoding a Hanks‐type kinase is a hallmark. Further studies on EcfP, the homolog of EcfK in *Vibrio parahaemolyticus*, revealed its direct phosphorylation by the cognate Hanks‐type kinase PknT when co‐expressed in *Escherichia coli* cells [18]. Notably, the sole phosphorylated residue identified in *V*. *parahaemolyticus* EcfP corresponds to T51 of EcfK.

In this study, we describe the direct phosphorylation of EcfK by the Hanks‐type kinase PknS of *X. citri*, revealing additional target residues that are required for sigma activation and T6SS function. We also describe the biochemical characterization of PknS and the structure of its kinase domain. Altogether, our findings provide new insights into this novel mechanism of signal transduction in bacteria, involving direct phosphorylation of a sigma factor.

## Results

### PknS is a typical Hanks‐type receptor kinase that catalyzes autophosphorylation

**Fig. 1:**
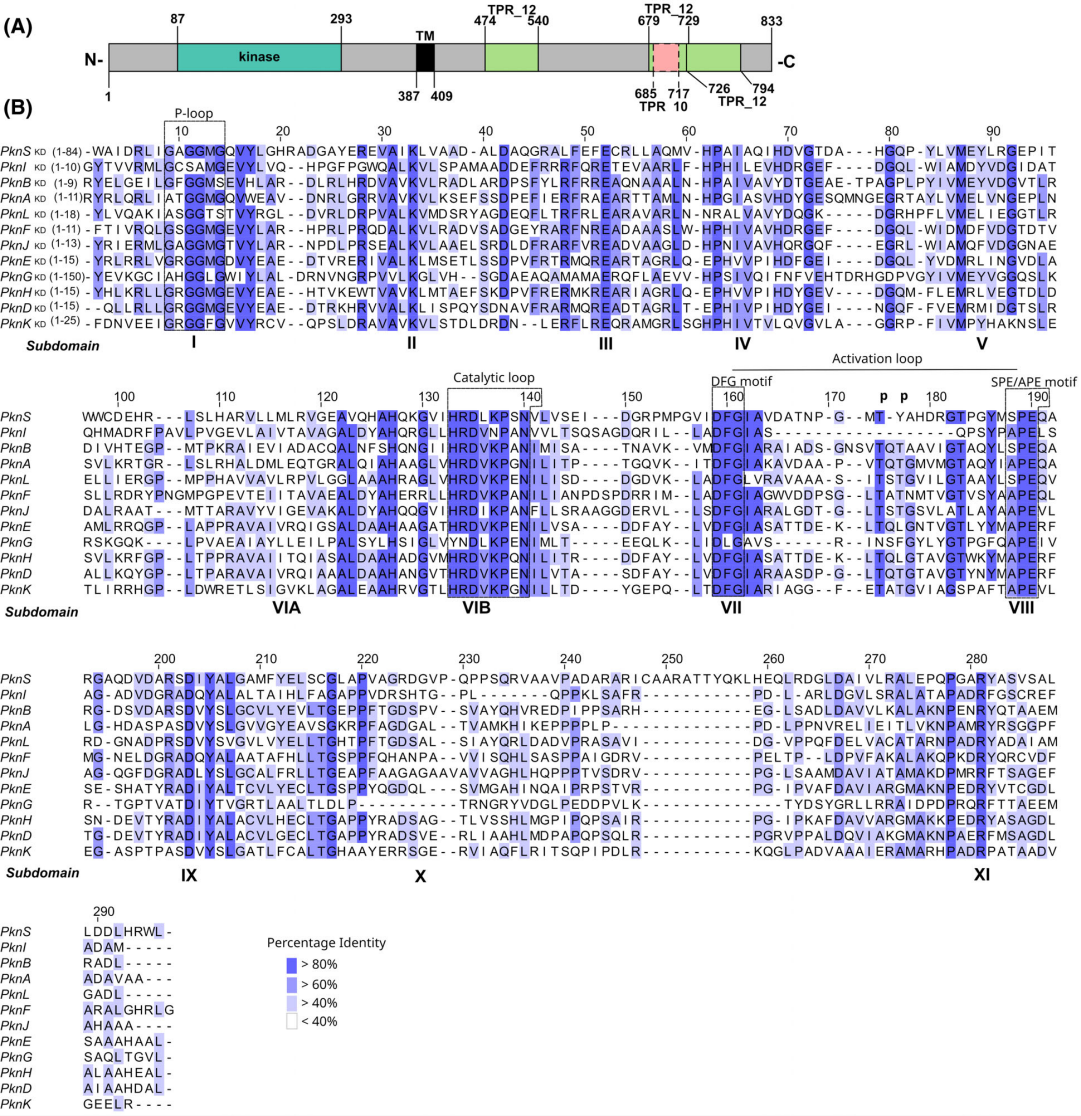
PknS is a canonical Hanks‐type receptor kinase. (A) Schematic representation of PknS (833 amino acids). Conserved domains and predicted transmembrane helix (TM) are depicted by colored boxes. The tetratricopeptide repeat domains (TPR) were identified using the PFAM database. (B) Protein sequence alignment of the PknS kinase domain and the corresponding domains of the 11 Ser/Thr protein kinases (STPKs) from *M. tuberculosis*. The 11 conserved subdomains (I–XI) that define the STPK family are indicated. Conserved residues are highlighted in blue, with shading intensity reflecting the percentage identity at each position. Functional loops are outlined by rectangles. The two major phosphosites identified in *M. tuberculosis* PknB are indicated above the alignment (p). Sequences were obtained from the KEGG database (https://www.genome.jp/kegg/): Rv0410c (PknG); Rv3080c (PknK); Rv0015c (PknA); RV2176 (PknL); Rv0014c (PknB); Rv0931c (PknD); Rv1266c (PknH); Rv1743 (PknE); Rv2088 (PknJ); Rv2914c (PknI); Rv1746 (PknF). Alignments were performed using muscle software [74] and images produced using jalview (https://www.jalview.org/).

PknS is a transmembrane receptor kinase, containing an amino‐terminal cytoplasmic region that encompasses the kinase domain and a carboxy‐terminal periplasmic region characterized by three tetratricopeptide repeats (TPRs), which are typically involved in protein–protein interactions (Fig. 1A). Additionally, PknS presents an extended 83 amino acids segment at its amino terminus, preceding the kinase domain, which is conserved among *Xanthomonadaceae* homologs and may play a functional role. A protein alignment of the PknS kinase domain (PknS_KD_) with the 11 *M. tuberculosis* Hanks‐type kinases demonstrated the presence of the 11 conserved subdomains (I–XI) that define the family (Fig. 1B) [1]. Moreover, PknS presents all key conserved kinase motifs required for ATP binding, kinase activation and substrate phosphorylation: the Gly‐rich P‐loop (GxGGMG motif) for ATP binding; the catalytic loop (H/YRDXKXXN), where aspartate (D) acts as the catalytic residue; and the activation loop, including the Mg^2+^‐binding DFG motif, the phospho‐acceptor threonine and the P + 1 APE/SPE motif (Fig. 1B), which influences substrate specificity. The lower conservation observed in the activation loop is consistent with its role in substrate recognition. Overall, PknS_KD_ shares 27–37% sequence identity with *M. tuberculosis* kinases, with PknB_KD_ showing the highest sequence identity (36.6%). PknB is an essential kinase in *M. tuberculosis* and, similarly to PknS, contains a transmembrane domain and an extracellular sensor module that regulates autophosphorylation and consequent activation of the kinase domain [19].

**Fig. 2:**
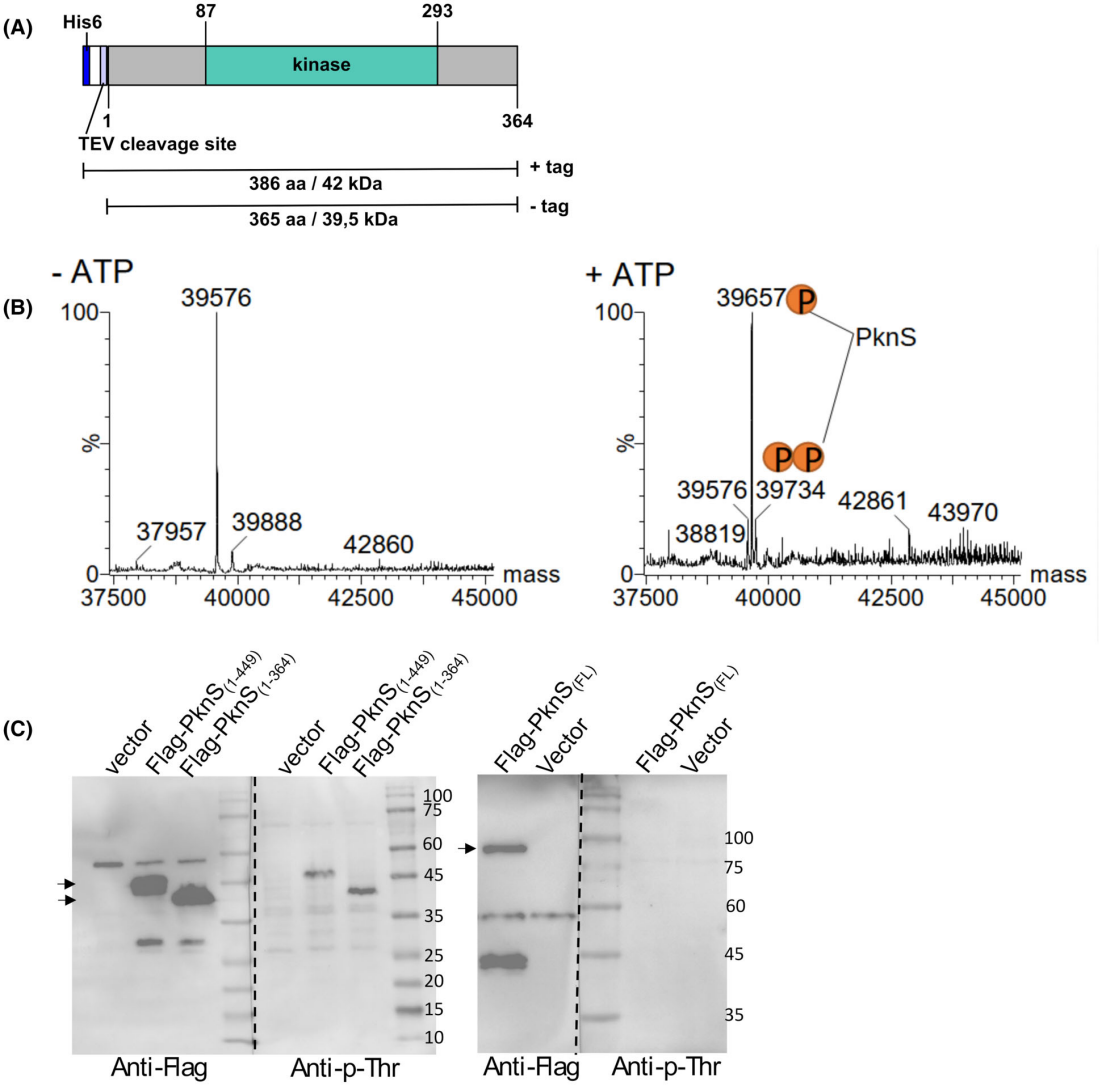
PknS_(1–364)_ autophosphorylates at two residues *in vitro*, with four potential phospho‐residues. (A) Schematic representation of PknS_1–364_. (B) Autophosphorylation detection by LC‐MS (Liquid chromatography–mass spectrometry). Deconvoluted mass spectra of PknS_1–364_ after incubation in the presence or absence of ATP. The original protein mass (39 576 Da) is observed in the absence of ATP. The phosphorylated proteins appear as mass shifts of +80 and +160 Da, indicating the occurrence of 1 or 2 phosphorylations, respectively (*n* = 3). (C) PknS_1‐364_ and a membrane‐tethered version, PknS_(1–449)_, but not the full‐length PknS, are phosphorylated in *X. citri* cells in the absence of an inducing cue. Immunoblot detection of phosphorylated proteins in total cell lysates from *X. citri* cells using a phosphothreonine‐specific antibody (anti‐p‐Thr). Total levels of PknS expression in each strain were also simultaneously analyzed with an anti‐Flag antibody (left side membranes). Flag‐tagged versions of PknS were ectopically expressed from an arabinose‐inducible promoter in the Δ*pknS* strain. A strain carrying the empty vector was used as a control. Protein bands corresponding to the expected mass of Flag‐PknS_1–364_ (40.9 kDa), Flag‐PknS_(1–449)_ (49.9 kDa), and Flag‐PknS_(FL)_ (98.9 kDa) are indicated by arrows. Results are representative of three experiments.

The cytoplasmic portion of PknS – PknS_(1–364)_—was expressed as a N‐terminal His‐tagged version and purified from the soluble fraction by affinity chromatography, followed by His‐tag cleavage and size exclusion chromatography (Fig. 2A, Fig. S1A). We tested whether PknS_(1–364)_ is catalytically active and capable of autophosphorylation *in vitro* upon incubation with ATP, as described for other Hanks‐type kinases. Mass spectrometry analysis (MS) detected a species with the expected molecular mass of PknS_(1–364)_ (39 576 Da) in the sample incubated in the absence of ATP, whereas the addition of ATP caused a mass shift consistent with autophosphorylation and incorporation of 1 or 2 phosphate groups (Fig. 2B, Fig. S1). These results demonstrated that the cytoplasmic portion of PknS is sufficient for catalytic activity and autophosphorylation.

To assess the phosphorylation of PknS in *X. citri* cells, we overexpressed the kinase fused to an N‐terminal Flag peptide in the Δ*pknS* strain and performed ⍺‐phosphothreonine western blotting. Signal transduction mediated by receptor kinases commonly involves ligand binding to their extracytoplasmic sensor domain, which may result in conformational changes, altered protein localization or promote kinase dimerization [20, 21]. To explore the effect of PknS’ distinct domains in its phosphorylation, we tested three variants of the kinase in *X. citri* cells: the soluble kinase domain (Flag‐PknS_(1–364)_); the membrane‐tethered kinase, Flag‐PknS_(1–449)_; and the full‐length protein (Flag‐PknS). These versions were expressed from an arabinose‐inducible promoter in a multicopy plasmid [14]. Immunoblot analysis of total extracts from *X. citri* cells detected anti‐p‐Thr reactive bands corresponding to the kinase domain Flag‐PknS_(1–364)_ and the membrane‐tethered kinase Flag‐PknS_(1–449)_, demonstrating that phosphorylation occurred *in vivo*, independently of the extracytoplasmic domain (Fig. 2C). In contrast, no phosphorylation signal was detected for the full‐length protein, suggesting that the extracytoplasmic region modulates the phosphorylation of PknS in the absence of an inducing cue (Fig. 2C). Accumulation of PknS versions was monitored simultaneously using an antibody against the Flag epitope, which confirmed that lack of detection with anti‐p‐Thr was not caused by low levels of expression of the full‐length PknS (Fig. 2C). The functionality of the full‐length Flag‐tagged version of PknS was validated by its ability to complement the Δ*pknS* mutant phenotype, restoring resistance to amoeba predation in phagocytic plaque formation assays (Fig. S2) [22]. Importantly, the two truncated versions of PknS failed to complement the pknS deletion, suggesting that the extracytoplasmic domain is necessary for the PknS‐mediated signaling under conditions that require T6SS activation (Fig. S2). These findings indicate that the periplasmic domain may play a role in proper PknS localization and/or full kinase activation to promote transphosphorylation.

### Structure of PknS_1–364_

Hanks‐type kinases share a conserved catalytic fold across both prokaryotes and eukaryotes [4]. Despite this structural conservation, they remain relatively poorly characterized in eubacteria, with the notable exception of *Mycobacterium* species, where they have been extensively studied [6]. In addition to PknS, the genome of *X. citri* pv. *citri* 306 encodes at least three other Hanks‐type kinases, XAC4116, XAC1171, and XAC1912, each sharing ~ 40% sequence identity with PknS. However, no structural information is currently available for any *Xanthomonas* Hanks‐type kinase. To address this gap, we pursued the crystallization and structural characterization of PknS to confirm its conserved Ser/Thr kinase fold and investigate its potential regulatory mechanisms.

**Fig. 3:**
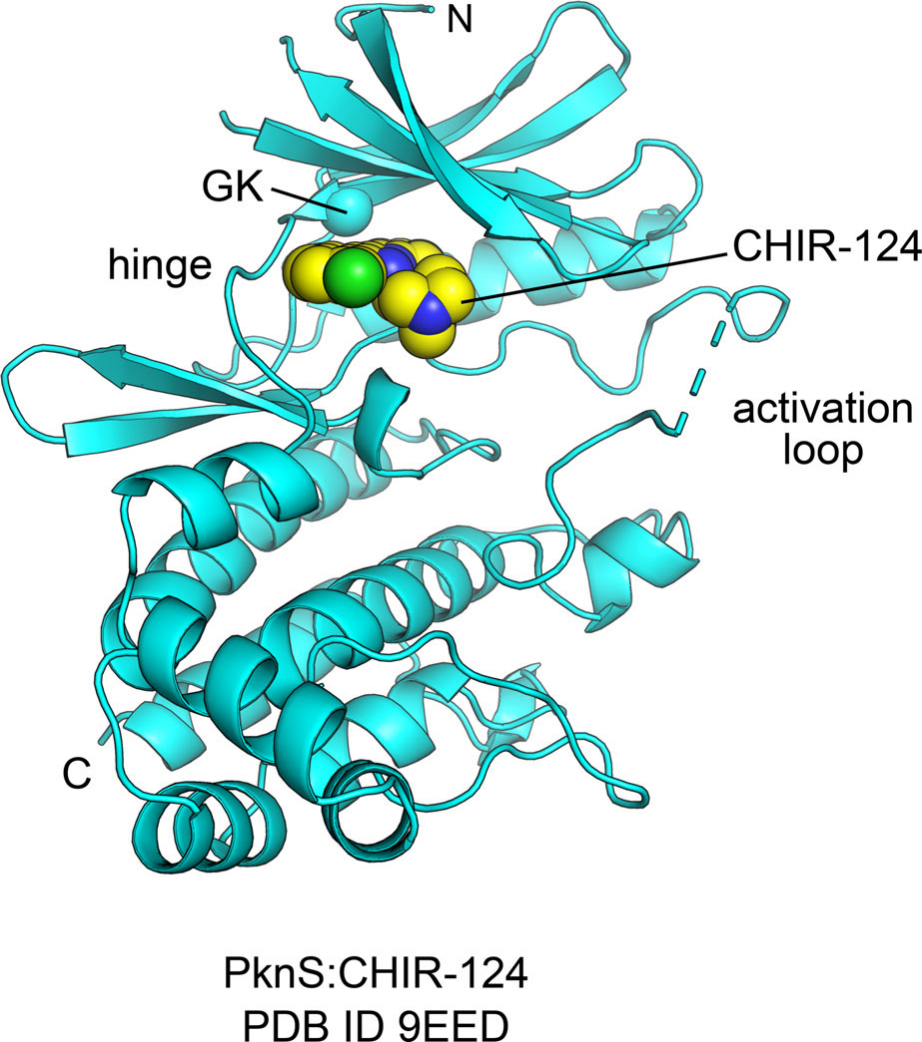
Cocrystal structure of PknS_1–364_M_164_A bound to the ATP‐competitive inhibitor CHIR124 adopts the conserved Hanks‐type kinase fold. Cartoon representation of PknS_1–364_M_164_A protein backbone (cyan). Only residues 82–104, 111–242, and 246–363 could be modeled. Missing residues, including PknS 81 N‐terminal residues were not visible in electron density maps. Missing internal loops are depicted as dashed cyan lines. Ligand (CHIR‐124) atoms are shown as spheres. The C atom of the gatekeeper residue (GK; Ala164) is shown as a cyan sphere. N‐ and C‐termini are indicated by N and C, respectively. Image produced using pymol Molecular Graphics System, Version 3.0.0 (Schrodinger, New York, NY, EUA).

Initial crystallization attempts of the PknS kinase domain (PknS_1–364_) were unsuccessful. However, crystals were obtained for a gatekeeper mutant (PknS_1–364_M_164_A) cocrystallized with CHIR‐124, an ATP‐competitive inhibitor originally developed for the human kinase Chk1 (Fig. 3) [23, 24]. CHIR‐124 was identified as a binder to PknS_1–364_M_164_A using a thermal shift assay in a screen of ~ 400 human kinase inhibitors (Table 1, Table S1) [25, 26]. The cocrystals of PknS_1–364_M_164_A with CHIR‐124 were solved at 2.1 Å resolution in space group P41 (Fig. 3, Table S2). The first 81 residues of PknS were missing from the electron density maps, likely due to flexibility. AlphaFold2 [27] predicts this region to fold into three to four antiparallel α‐helices connected to the kinase domain via a flexible linker (Fig. S3). Although no electron density could be found for these secondary structures, we did observe a large vacant space within the cocrystal lattice that could potentially accommodate PknS N‐terminal residues as predicted by AlphaFold2. The final model of PknS_1–364_M_164_A consisted of residues 82–104, 111–242, and 246–363. Missing protein segments mapped to likely flexible loop regions in the AlphaFold2‐predicted structure (Fig. S3). This included part of the kinase activation loop spanning the conserved residue Thr243, which is likely important for protein function.

**Table 1:** Differential scanning fluorimetry (DSF) analysis of wild‐type PknS (PknS_1–364_) and PknS_1–364_M_164_A using the compound library Selleckchem. Only compounds that caused a thermal shift > 2.0 are depicted; those with the highest thermal shift are highlighted in red. The results for all the tested compounds are described in Table S1.

PknS_1–364_‐M_164_A		PknS_1–364_	
Ligand	ΔTm B	Ligand	ΔTm B
CHIR‐124	6.2	Tyrphostin 9	4.5
TPCA‐1	4.3	AZD3463	3.4
AT9283	4.2	BIRB 796 (Doramapimod)	2.8
AEE788 (NVP‐AEE788)	4.0	JNJ‐7706621	2.5
Dabrafenib (GSK2118436)	3.8	Quercetin	2.2
SGI‐1776 free base	3.7	TPCA‐1	2.0
BI‐D1870	3.0		
Dasatinib	2.9		
TWS119	2.8		
MK‐8776 (SCH 900776)	2.2		
PLX‐4720	2.2		
JNJ‐7706621	2.1		
BIX 02189	2.0		

The cocrystal structure confirmed that PknS adopts the conserved Hanks‐type kinase fold, characterized by an N‐terminal lobe composed predominantly of β‐strands and a C‐terminal lobe dominated by α‐helices. These lobes are connected by a flexible hinge region (residues 165–172 in PknS) that forms part of the ATP‐binding site, a hallmark feature of protein kinases (Fig. 3). Structural alignment using PDBefold [28] revealed significant similarity between PknS and other serine/threonine kinases, including PknA and PknB from *M. tuberculosis* and the human kinase BRAF. Despite low sequence identity (< 35%), the root mean square deviations (RMSDs) for Cα atoms in structurally equivalent regions of PknS and *M. tuberculosis* PknA and PknB and human BRAF ranged from 1.5 to 2.0 Å.

As expected, the ATP‐competitive inhibitor CHIR‐124 was found bound to the ATP‐binding site of PknS. The quinolinone moiety of CHIR‐124 formed hydrogen bonds with the main‐chain atoms of Leu167 in the kinase hinge region. Hydrophobic interactions were observed between CHIR‐124's benzyl imidazole and quinolinone groups and the side chains of Ile91, Val99, Ala114, Tyr166, Leu216, and Ile229. In contrast, the chlorine atom of CHIR‐124 pointed towards the solvent and did not seem to engage with any protein atoms. Interestingly, this binding mode differed from that observed for the cocrystal of CHIR‐124 bound to the human kinase Chk1 [24]. In Chk1, CHIR‐124's chlorine moiety interacts with the bottom of the ATP‐binding pocket, whereas in PknS, it points towards the solvent.

The M164A gatekeeper mutation significantly altered the ATP‐binding pocket organization of PknS. In protein kinases, the so‐called gatekeeper residue determines the accessible volume of the ATP‐binding site for ATP‐competitive ligands [29]. The smaller alanine gatekeeper in the PknS mutant accommodated CHIR‐124's bulky benzyl imidazole moiety, whereas the wild‐type methionine would likely impose steric hindrance. Consistent with this observation, CHIR‐124 did not bind wild‐type PknS in the thermal shift assay. Instead, Tyrphostin 9, a smaller molecule, emerged as the most potent binder for the wild‐type enzyme (Table 1, Table S1) [30]. In fact, fewer inhibitors from the tested library bound the wild‐type enzyme compared to the M164A mutant, suggesting stricter steric constraints in the wild‐type ATP‐binding site. Nevertheless, some bulkier inhibitors, such as BIRB‐796 [31, 32], were identified as wild‐type PknS binders, indicating that steric compatibility is not the sole determinant of binding affinity (Table 1).

In conclusion, the structure of PknS_1–364_M_164_A bound to CHIR‐124 provides further insights into the conserved Ser/Thr kinase fold and highlights the structural consequences of gatekeeper mutations on ATP‐binding site architecture. These findings enhance our understanding of kinase–inhibitor interactions and may inform the design of selective inhibitors for bacterial kinases.

### PknS directly phosphorylates the ECF sigma factor EcfK

**Fig. 4:**
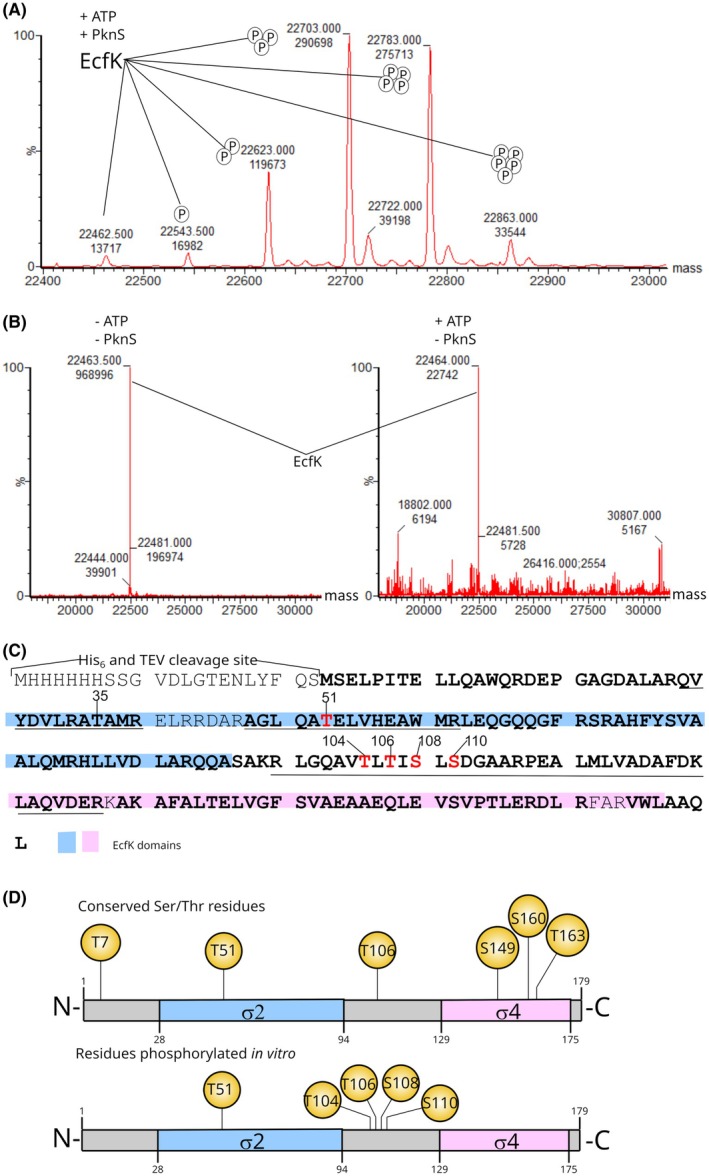
EcfK is directly phosphorylated by PknS at five phosphosites. (A, B) EcfK phosphorylation detection by liquid chromatography–mass spectrometry (LC–MS). Deconvoluted mass spectra of His_6_‐EcfK after incubation with PknS and ATP (A), in the absence of PknS and ATP (B, left panel) and in the absence of PknS and in the presence of ATP (B, right panel), as indicated above each spectrum. The original protein mass (22 463 Da) was identified in the samples incubated in the absence of PknS (B). Phosphorylated EcfK species were identified in phosphorylation reactions as mass shifts of +80 Da increments, corresponding to the addition of 1–5 phosphate groups (A) (*n* = 3). (C) Liquid chromatography–tandem mass spectrometry (LC‐MS/MS) analysis of EcfK after incubation with PknS and ATP. Phosphorylated residues consistently identified in more than one peptide and presenting a score > 7.0 are highlighted in red color. Phosphorylation at residue T35 was identified in a single peptide and was therefore excluded from further analysis. The sequence coverage of EcfK is highlighted in bold (refer to Table S4). Phosphorylated peptides are underlined (refer to Table S3). The conserved σ_2_ and σ_4_ domains are highlighted in blue and pink, respectively. (D) Schematic representations of EcfK, depicting the conserved Ser/Thr residues previously identified and tested for phospho mimicking mutations (upper figure) and the position of the experimentally verified phosphosites (lower figure).

Our previous studies on the role of PknS and EcfK in the regulation of *X. citri*'s T6SS provided evidence for a signal transduction pathway that possibly involves the direct phosphorylation of EcfK by PknS [14]. To test this hypothesis, we used purified EcfK alongside the functional PknS_1–364_. The full‐length EcfK was expressed in *E. coli* as an N‐terminal fusion to a polyhistidine tag and purified from inclusion bodies, resulting in a highly pure fraction with the expected protein mass (Fig. 4B, Fig. S4). We then performed *in vitro* phosphorylation reactions by co‐incubation of His_6_‐EcfK and PknS_1–364_ in the presence of ATP, followed by mass spectrometry to determine the intact mass of EcfK. Interestingly, five new EcfK species were detected following the phosphorylation reactions, which corresponded to cumulative increments of up to five phosphates in the protein (Fig. 4A, Fig. S5). These species were not detected in EcfK samples incubated with ATP in the absence of PknS (Fig. 4B, Fig. S6). To further identify the residues that are targeted for phosphorylation by PknS, we performed LC‐MS/MS analysis of trypsin‐digested samples. Notably, results from this analysis identified with high confidence the conserved residue T51 as a phosphosite, corroborating our previous work that described the constitutive activation of EcfK by a phosphomimetic version of T51 (T51E) [14]. In a similar fashion, Iyer *et al*. [18] demonstrated that the EcfK homolog EcfP of *V. parahaemolyticus* is phosphorylated at the corresponding residue by a PknS homolog. However, in contrast to the unique phosphosite described for EcfP, four additional phosphorylation sites were consistently identified in EcfK, albeit with less confidence, namely T104, T106, S108, and S110, which are located in the linker region between the conserved σ2 and σ4 domains (Fig. 4C,D, Tables S3–S5, Fig. S7). Among these residues, T106 is one of six conserved threonines found in members of the ECF43 subfamily (Fig. 4D). Our previous study demonstrated that the phosphomimetic mutant variant T106E retained functionality for *in vivo* complementation of the Δ*ecfK* strain, despite lacking constitutive activation via phospho‐mimicry [14].

Bacterial σ factors of the σ^70^ family contain up to four conserved modular domains (σ_1_–σ_4_), but members of the ECF subfamily typically contain only two of them, σ_2_ and σ_4_, which recognize the −10 and −35 promoter regions, respectively [33]. In the primary σ factors (homologs to *E. coli* σ^70^), domain σ_1_ inhibits binding of the free σ to DNA and also recognizes an extended σ^70^‐specific promoter element, while domains σ_2_, σ_3_, and σ_4_ coordinate all σ functions, which include −10 and −35 promoter recognition, transcription initiation, abortive transcription, pausing, and promoter escape [33]. In contrast, ECF σ factors are more divergent and contain a nonconserved linker connecting domains σ_2_ and σ_4_. Recent structures of ECF‐containing transcription initiation complexes demonstrated that the linker traverses the RNA polymerase active center, playing a similar role as domain σ_3_ of primary σs during holoenzyme formation and transcription initiation [34, 35, 36]. EcfK residue T51 is located in a conserved region of the σ_2_ domain (σ_2.2_) that, in primary σs and ECF σ factors not associated with STPKs, forms a polar surface and contains negatively charged residues that make direct contacts with the RNA polymerase core (DxxDxxQE), as demonstrated by several genetic and structural studies [34, 37, 38, 39, 40]. ECF43 members typically contain amino acid substitutions for noncharged amino acids in the consensus motif DxxD within this region, with a Thr residue corresponding to EcfK position T51 conserved in 88% of the ECF43 family members (QTTA consensus motif for ECF43, where the phosphothreonine is underlined) [18]. Therefore, phosphorylation of this conserved Thr in ECF43 members is proposed to activate the σ factor by imparting the required negative charge to the σ_2.2_ domain, thus promoting interaction with the β'subunit of the RNA polymerase. This model was based on functional studies of *V. parahaemolyticus* EcfP, in which all negatively charged residues are absent. Notably, however, the corresponding motif in EcfK is QATE, retaining a negative charge adjacent to the phospho residue (Fig. 4C). A protein alignment of all ECF43 members showed 15.8% conservation of a negative charge in this position [18]. Moreover, the phospho ablative variant EcfK^T51A^ is functional in *X. citri*, as it is sufficient to restore the resistance to amoeba predation of the Δ*ecfK* strain, suggesting that other phospho sites account for the σ function [14]. Altogether, these results demonstrate that EcfK is directly phosphorylated by PknS and indicate that activation of ECF43 family members may involve additional phosphosites located in the linker region of sigma factors.

**Fig. 5:**
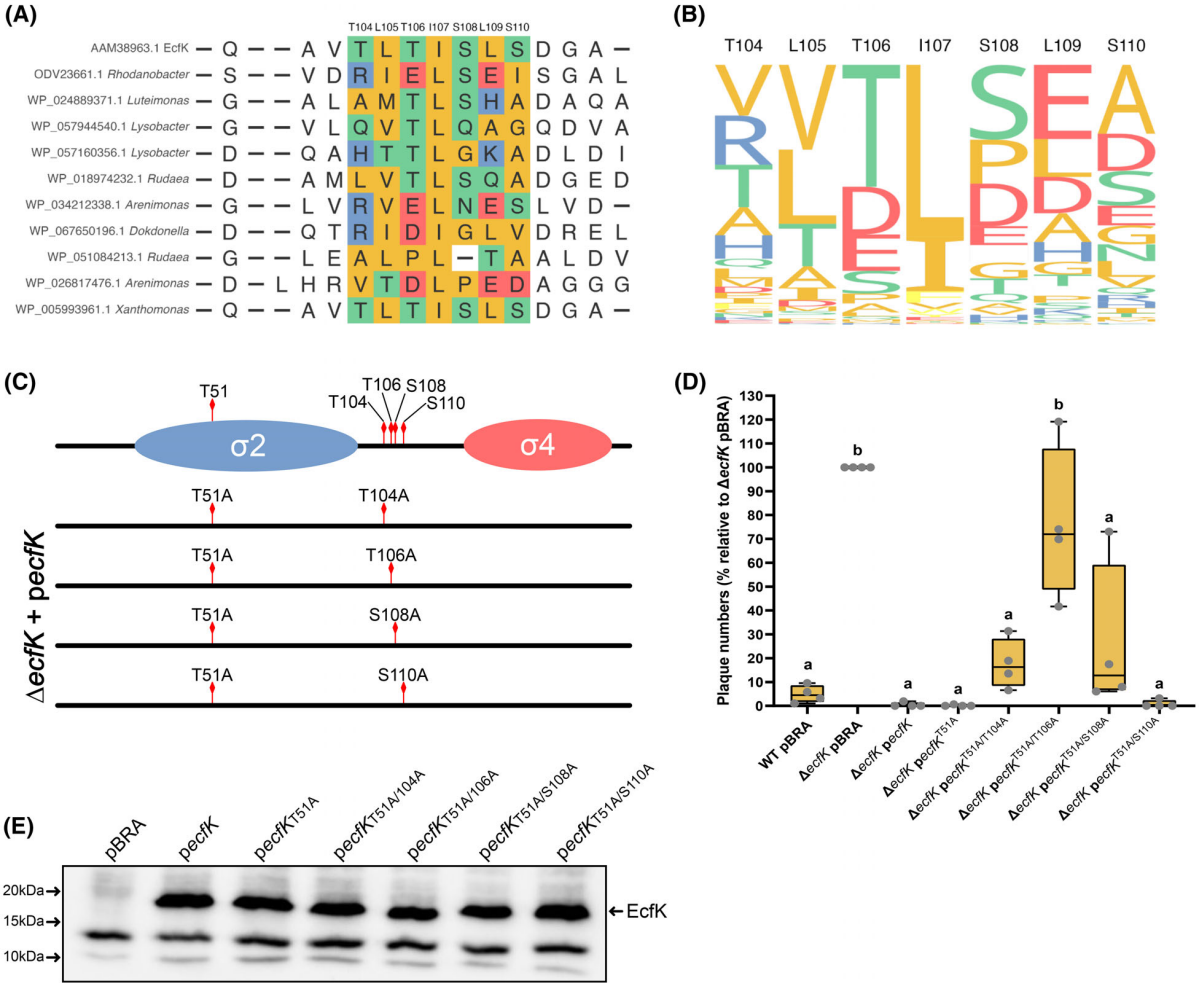
T51 and T106 residues are required for EcfK function *in vivo*. (A) Protein sequence alignment of EcfK with all ECF43 representatives from Xanthomonadales. For simplicity, the figure includes 10 randomly selected representatives from the general alignment. Colored amino acids correspond to the region spanning residues T104‐S110 of EcfK. Amino acids are colored by side‐chain chemistry: red, acidic; blue, basic; green, polar uncharged; orange, hydrophobic; yellow, aromatic. NCBI protein accession numbers precede each genus name. Sequences were obtained from the dataset of [18]. Alignment was performed using the DECIPHER R package [75] and visualization was generated with the ggmsa R package [76]. (B) Sequence logo of amino acids spanning the loop region corresponding to residues T104 to S110 of *X. citri* EcfK. (C) Schematic representation of EcfK, depicting the conserved σ2 and σ4 domains and the position of threonine and serine residues phosphorylated by PknS *in vitro*. Each line below the scheme shows the evaluated combinations of mutant variants of EcfK, which included T51A and one of the four phospho‐residues located in the loop region (amino acid substitutions to alanine, rendering the residues nonphosphorylatable). (D) Sensitivity of *X. citri* strains to *D. discoideum* predation by phagocytic plaque assays. Experiments were performed using the Δ*ecfK* complemented with plasmids expressing the mutated versions of *ecfK* (as depicted in C) under the control of the *P*_BAD_ promoter. Wild‐type (WT) and Δ*ecfK* strains carrying the empty vector or constructs expressing *ecfK* and *ecfK* ^T51A^ were used as controls. Bacterial and amoeba cells were mixed in SorC buffer and applied onto N agar plates. Predation sensitivity is scored by the number of amoeba plaques formed onto the bacterial lawn for up to 7 days. Results are shown as the percentage of plaques formed after 7 days relative to the positive control for plaque formation (T6SS mutant, Δ*ecfK* pBRA). The experiment was repeated four times (each small circle in the plots), and the letters on top of each plot indicate strains with statistically equal sensitivity (Dunnett's multiple comparison test, alpha 0.05). All strains marked as distinct (“a”) from the T6SS mutant strain used as control for plaque formation (Δ*ecfK* pBRA, ‘b’) presented an adjusted *P* value < 0.0001. Error bars indicate standard deviation. Individual experiments are shown in Fig. S9. (E) Combined mutations in *ecfK* phospho‐acceptor residues do not affect EcfK protein stability. Strains carrying the indicated plasmids were grown in LB (lysogeny broth medium) and EcfK levels were estimated by western blotting using a polyclonal anti‐EcfK antibody (*n* = 1).

To further analyze the conservation of the loop region of the ECF43 subfamily, we performed a protein alignment including 1032 ECF43 sequences, obtained from Iyer *et al*. [18]. Despite a low overall sequence conservation in the loop, the position corresponding to residue T106 contains a Ser/Thr or the negatively charged residues Asp/Glu in 76.9% of all ECF43 members analyzed, reaching even higher conservation (87.9%) when only the 343 Xanthomonadales representatives are used for the alignment (Tables S6 and S7, Fig. 5A,B). In addition to position EcfK^T106^, we also observed an overrepresentation of Ser/Thr/Asp/Glu amino acids in the position corresponding to EcfK^S108^, that is, 62.5% and 63.3% among Xanthomonadales and all ECF43, respectively (Tables S6 and S7).

### Biological effect of phosphorylation: Phospho ablative mutations at residues T51 and T106 abolish EcfK activation

To determine the contribution to σ activation of each of the four phosphosites clustered in the EcfK linker region, we tested the functionality of EcfK mutant variants *in vivo*. For that, we evaluated their ability to restore the resistance to amoeba predation in a Δ*ecfK* strain when expressed from a multicopy vector. Since the mutant variant EcfK^T51A^ is functional for complementation, we combined phospho ablative mutations in each residue with the previous functional mutant variant T51A, generating double mutant versions (*ecfK* ^T51A/T104A^; *ecfK* ^T51A/T106A^; *ecfK* ^T51A/S108A^; *ecfK* ^T51A/S110A^) (Fig. 5C). The double mutants were generated by site‐directed mutagenesis using a multicopy plasmid expressing *ecfK* ^T51A^ under control of an arabinose‐inducible promoter as template [14]. Immunoblot analysis using a polyclonal antibody against EcfK confirmed that all mutant versions are stably expressed in *X. citri* (Fig. 5E).

The Δ*ecfK* strain carrying the nonphosphorylatable mutant variants (Δ*ecfK ecfK* ^T51A/T104A^; Δ*ecfK ecfK* ^T51A/T106A^; Δ*ecfK ecfK* ^T51A/S108A^; Δ*ecfK ecfK* ^T51A/S110A^) was used in a quantitative assay that evaluates the efficiency of amoeba predation upon co‐incubation with bacteria on solid medium. The efficiency of predation is calculated as the number of clear halos (phagocytic plaques) formed on a bacterial lawn as a result of amoeba grazing and replication, with no plaques formed in co‐incubations with amoeba‐resistant bacteria [41]. A *D. discoideum* sensitive strain of *Klebsiella pneumoniae* (nonvirulent strain) was used as a control, showing a high number of plaques formed from 4 to 7 days of co‐incubation (Fig. S8). No plaques were formed on the lawn of the *X. citri* WT strain carrying the empty vector after 7 days, confirming the virulent phenotype of *X. citri*. As expected based on previous work, the Δ*ecfK* strain carrying the empty vector was highly sensitive to predation when compared to the WT strain, and resistance was restored by complementation with *ecfK* and *ecfK* ^T51A^ in the multicopy vector (Fig. 5D, Fig. S8) [14]. In contrast, combining the T51A with T106A mutation abrogated EcfK functionality, as transformation with *ecfK* ^T51A/T106A^ was not sufficient to efficiently complement the deleted strain (Fig. 5D, Fig. S8). Importantly, very few plaques were observed in the Δ*ecfK* strain carrying the other mutant combinations (*ecfK* ^T51A/T104A^; *ecfK* ^T51A/S108A^ and *ecfK* ^T51A/S110A^) after 7 days of co‐incubation, demonstrating the functionality of these phospho ablative variants (Fig. 5D, Fig. S8). These results suggest that phosphorylation of either T51 or T106 is required and sufficient for EcfK activation and consequent induction of the T6SS‐mediated resistance to amoeba.

### Phosphorylation of the EcfK residue Thr106 plays a crucial role in the interaction with the RNA polymerase

**Fig. 6:**
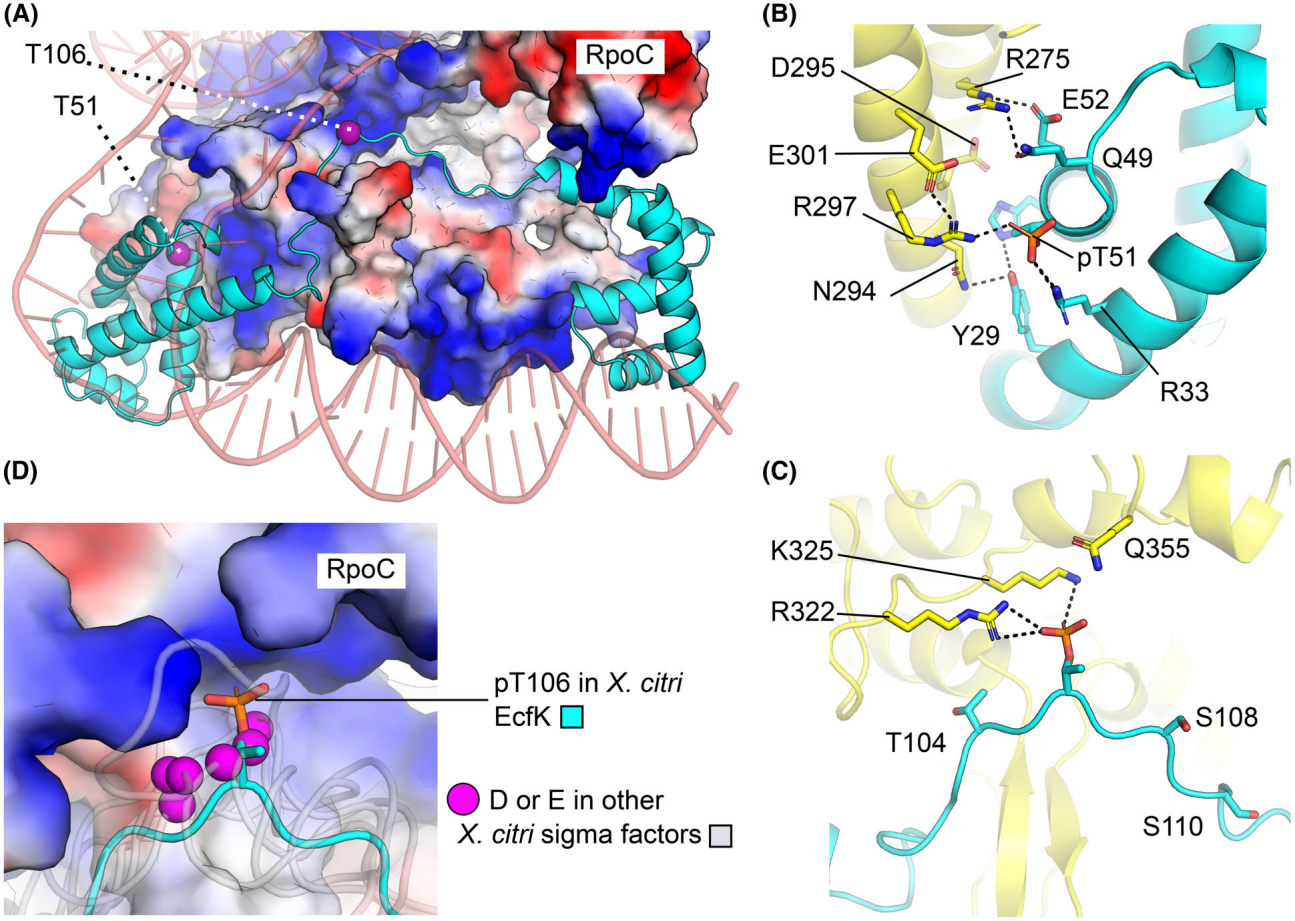
AlphaFold 3 (AF3) prediction of the *X. citri* holo‐RNA polymerase complex bound to EcfK suggests that phosphorylation of Thr106 plays a crucial role in the interaction between RNA polymerase and the sigma factor. (A) AF3‐predicted structure of the *X. citri* transcription initiation complex, highlighting the structures of EcfK (cyan cartoon) and RpoC (protein surface represented as an electrostatic potential map—blue indicates positive charge, red indicates negative charge). The locations of Thr51 and Thr106 are marked by magenta spheres. (B, C) Potential hydrogen bond interactions between residues in EcfK (carbon atoms and cartoon shown in cyan) and RpoC (carbon atoms and cartoon shown in yellow) near phosphorylated Thr51 (pThr51, B) and Thr106 (pThr106, C). Additional EcfK residues shown to be phosphorylated by PknS *in vitro* (Thr104, Ser108, and Ser110) are depicted as sticks. (D) Other *X. citri* sigma factors have negatively charged residues occupying positions structurally equivalent to pThr106 in EcfK. EcfK is shown as a cyan cartoon, and pThr106 is represented in a stick model. The following *X. citri* sigma factors are shown as semi‐transparent gray cartoons: RfaY (UNIPROT ID: A0AAI7ZGL0), PrtI (UNIPROT ID: A0AAI7ZIN4), RpoE (UNIPROT ID: A0AAI8ER26), XAC0922 (UNIPROT ID: A0AAI7ZDN4), XAC1682 (UNIPROT ID: A0AAI7ZET4), XAC2191 (UNIPROT ID: A0AAI7ZFJ4), and AlgU (UNIPROT ID: A0AAI8ES64). Negatively charged residues (Asp and Glu) in these sigma factors, located within 3 Å of pThr106 in EcfK, are highlighted as pink spheres (C⍺s). All images produced using pymol Molecular Graphics System, Version 3.0.0. See also Fig. S9.

To better understand how the phosphorylation of residues Thr51 and Thr106 may influence the interaction with the RNA polymerase and transcription of T6SS genes in *X. citri*, we used AlphaFold 3 (AF3) [42] to generate predictive models of *X. citri* holo‐RNA polymerase complexes (Fig. 6A). The AF3‐predicted model was in excellent agreement with cryo‐EM structures of transcription elongation complexes from related bacteria, including *X. oryzae* [PDB ID: 6J9E; root mean square deviation (RMSD) = 1.0 Å after superimposing 2570 equivalent C⍺ atoms] [43], *P. aeruginosa* (PDB ID: 7XYA; RMSD = 1.2 Å after superimposing 2613 equivalent C⍺ atoms) [44], and *E. coli* (PDB ID: 6JBQ; RMSD = 1.2 Å after superimposing 2435 equivalent C⍺ atoms) [34]. The DNA‐directed RNA polymerase subunits—alpha (RpoA), beta (RpoB), and beta’ (RpoC) – share > 99% sequence identity between *X. citri* and *X. oryzae*, and over 70% identity with the corresponding proteins in *P. aeruginosa* and *E. coli*.

These structural analyses revealed that phosphorylation of Thr51 and Thr106 may promote protein–protein interactions between EcfK and RpoC via charge complementarity and hydrogen bond formation (Fig. 6B,C). In the AF3‐predicted models, phosphorylated Thr51 (pThr51) is within hydrogen bond distance of RpoC residue Arg297. Notably, several polar residues near Thr51 in EcfK—specifically Arg33, Gln49, Glu52, His55, and Tyr29—contribute to an extensive hydrogen bond network involving RpoC residues Arg275, Asn294, Glu295, Arg297, and Glu301 (Fig. 6B). The presence of multiple residues capable of forming interactions with RpoC likely explains why mutating Thr51 to alanine does not disrupt EcfK‐mediated gene transcription in *X. citri*.

Similarly, the AF3‐predicted models suggest that phosphorylated Thr106 (pThr106) is positioned within a positively charged patch on the surface of RpoC and may form hydrogen bonds with RpoC residues Arg322 and Lys325 (Fig. 6A,C). Unlike pThr51, pThr106 appears to be the primary contact point between EcfK and RpoC in this region of the initiation complex. This finding is consistent with our experimental observations showing that a threonine‐to‐alanine mutation at position 106 affects gene expression in *X. citri* when combined with the T51A mutation.

To further evaluate the significance of the pThr106‐RpoC interaction, we used AF3 to generate predictive models of transcription initiation complexes with the seven other sigma factors encoded in the *X. citri* genome (Fig. 6D and Fig. S9). In the AF3‐predicted model of the transcription initiation complex bound to EcfK, Thr106 is located within a flexible loop region connecting the alpha‐helix‐rich domains of the sigma factor. This loop fits within a positively charged patch on the surface of RpoC. Our analyses indicate that six of the other seven *X. citri* sigma factors contain a similar flexible loop that interacts with the same region of RpoC (Fig. 6D). Importantly, these six sigma factors feature negatively charged residues within the loop, serving as structural equivalents of pThr106. The sole exception, XAC3989, displays a shorter intervening loop that lacks negatively charged residues, suggesting a distinct interaction mechanism with RpoC.

Altogether, these results demonstrate that the ECF43 family of sigma factors may have one or two sites of regulation by Hanks‐type kinases, as exemplified by *V*. *parahaemolyticus* EcfP and *X. citri* EcfK, respectively. These phosphosites are located in distinct domains that make direct hydrogen bond contacts with positively charged residues of the RNA polymerase core.

## Discussion

In this study, we used a combination of structural and functional approaches to characterize a Hanks‐type kinase from the phytopathogenic bacterium *X. citri*, also revealing the mechanism for direct activation of an alternative RNA polymerase sigma factor, which culminates with the induction of a T6SS. Notably, among the four STPKs of *X. citri*, two of them, PknS (XAC4127) and PpkA (XAC4116), are encoded within the anti‐amoeba T6SS gene cluster. Our previous work has described the role of PknS in the induction of T6SS gene expression in an EcfK‐dependent manner [14], whereas PpkA is homologous to STPKs that are involved in a post‐translational signaling pathway that controls T6SS assembly and firing events, also involving the cognate phosphatase PppA (XAC4117) [45]. Besides PknS, only XopAU has been functionally characterized, encoding a protein effector secreted directly to plant cells by the Type III secretion system (T3SS), the major virulence determinant of *Xanthomonas* species [46]. Phosphoproteomic analysis of *X. citri* cells has identified a subset of proteins that undergo differential phosphorylation depending on growth conditions, with the vast majority (> 90%) of phosphosites occurring on threonine and serine residues [47]. However, the STPK(s) responsible for this regulatory switch in response to growth conditions remains elusive. Additionally, serine phosphorylation by a yet unidentified STPK triggers induction of the *X. citri* T3SS in response to the plant environment [47]. Therefore, STPKs play an important regulatory role in the activation of at least two secretion systems of *X. citri* and in the bacterial adaptation to environmental conditions. Results hereby described contribute to the understanding of these important regulators in *X. citri*.

We demonstrate that the cytoplasmic domain of PknS catalyzes its autophosphorylation in up to two residues, suggesting a similar auto‐activation mechanism for PknS as described for canonical STPKs. Moreover, phosphorylation of the kinase domain was detected in *X. citri* cells under noninducing conditions (absence of amoeba cells) using two truncated versions that lack the periplasmic domain, while the full‐length protein was not phosphorylated. This result suggests a modulatory role of the periplasmic domain, possibly inhibiting the auto‐activation of PknS in the absence of a stimulus. This domain may play additional roles in PknS function *in vivo*, since overexpression of the truncated versions was not sufficient to rescue the function of PknS in a Δ*pknS* strain. In contrast to eukaryotic STKs, bacterial counterparts commonly present a transmembrane region and a periplasmic domain with a role in signal sensing, as exemplified by *M. tuberculosis* STPKs and *Pseudomonas aeruginosa* PpkA [45, 48]. A survey of STPKs associated with ECF43 members has identified a wide variety of extracytoplasmic domains [18]. Typically, extracytoplasmic regions of STPKs bind ligands and promote kinase dimerization, which may activate autophosphorylation and kinase activity. Among some common extracytoplasmic domains in STPKs, we can cite PASTA domains found in many Gram‐positive bacteria and in *M. tuberculosis* PknB, which binds muropeptides and is involved in cell wall synthesis. The PASTA domain of *Bacillus subtilis* PrkC is required for dimerization and kinase activation during the stationary phase, while kinase activation relies on the cell division protein GpsB and occurs independently of the extracellular region in exponential phase cells, revealing distinct mechanisms of kinase activation according to growth conditions [49]. Similarly, PknS may be activated differently during growth in the presence of amoeba cells, requiring the periplasmic TPR‐rich domain for autophosphorylation and activation. PASTA domains of STPKs have also been involved in proper localization of the kinase at the division septum, as shown for *Streptococcus pneumoniae* StkP [50]. Similarly, PknB PASTA domains are required for protein localization at the cell poles [19, 20]. Remarkably, ligand binding to PknB PASTA domains modulates kinase activation by avoiding hyperphosphorylation and consequent cell death [19]. Future studies will be focused on the mechanisms of PknS activation through its periplasmic region in response to signals for the presence of predatory amoeba.

Our work demonstrates the direct phosphorylation of a sigma factor by PknS, further identifying five residues that are targeted by the kinase *in vitro*. This work builds upon our previous study that described the functional characterization of phosphomimetic EcfK mutants and pointed to a possible direct mechanism of activation of EcfK by phosphorylation [14]. The direct phosphorylation of an ECF43 family member has been described for EcfP of *V. parahaemolyticus*, and a unique phosphosite at the σ_2.2_ domain was identified as the switch required to promote σ activation by increased binding to the RNA polymerase [18]. We used a combination of *in vitro* assays with purified EcfK and PknS, functional analysis by complementation of a *X. citri ecfK* mutant strain, and protein structure prediction to demonstrate that EcfK presents a different mode of activation that involves two possible phosphosites as switches for σ activation by the kinase. In addition to the σ_2.2_ identified in EcfP, we describe the phosphorylation of residues located in the loop region between σ_2_ and σ_4_, which mediates direct contacts with the RNA polymerase core in σ^ECF^ group members, having a similar role as the σ_3_ domain found in non‐ECF σ^70^ [34, 35, 36]. Protein sequence alignments showed conservation of Ser/Thr residues or the presence of negatively charged amino acids (Glu/Asp) in the position corresponding to the phospho residue EcfK T106 in the loop region of ECF43 homologs (Fig. 5A,B and Tables S6 and S7). Structural models of the complex between EcfK and the core RNA polymerase further suggested that the negative charge imparted by phosphorylation of residue T106 is required for hydrogen bonding at the contact surface with RNA polymerase, which is substituted for negatively charged residues in other *X. citri* σ^ECF^ nonassociated with STPKs. Based on these results, we propose that different mechanisms of activation by STPKs are found within the ECF43 group, which may involve one or two points of contact with the RNA polymerase core. The presence of different sites of activation by the cognate STPK may be important to fine‐tune the response to a stimulus, which may be further controlled by Ser/Thr phosphatase‐mediated inactivation. The effects of multisite Ser/Thr phosphorylation have been well‐described for some eukaryotic transcription factors, and the distinct levels of phosphorylation and/or targeted phosphosites may differentially affect the magnitude of protein activation/inhibition, protein stability and allow for the integration of multiple signals [51]. Interestingly, plant plastid sigma factors contain multiple phosphorylation sites at a nonconserved region located at the N terminus, and they may differently affect the specificity for promoter binding [51, 52, 53].

Phosphorylation of a σ factor has been first described for the *M. tuberculosis* SigH, which is not a member of the ECF43 family of STPKs associated ECFs and is directly phosphorylated in two residues by PknB [54]. Notably, two phospho‐residues have been identified within the loop region between domains σ2 and σ4 of SigH, T106, and T110, and the structure of the holoenzyme (PDB 5ZX3) indicates that the negatively charged residue adjacent to SigH T106, E107, engages in hydrogen bond contacts with a positively charged pocket of the RNA polymerase core, while SigH T106 seems to be involved in intramolecular interactions. SigH activity is inhibited by interaction with the anti‐σ factor RshA, which is also phosphorylated by PknB, resulting in the release of the σ to bind to the core RNA polymerase. In contrast, SigH phosphorylation does not affect its interaction with RshA, and the biological significance of this post‐translational modification has not been further investigated [54]. Our results reveal new insights into the role of a transmembrane STPK in the activation of a ECF43 sigma factor, forming a novel signal transduction pathway that enables *X. citri* to monitor and respond to environmental changes. The identification of distinct domains within the σ factor that are regulated through phosphorylation suggests a mechanism to enhance the dynamics of the response, possibly enabling various levels of σ activation and/or regulatory inputs. The structure and autophosphorylation of PknS hereby described further contribute to the understanding of this important family of molecular switches that are relatively poorly characterized in bacteria.

## Materials and methods

### Culture conditions

Bacterial and *D. discoideum* strains used in this study are listed in Table S8 [55, 56, 57]. *X. citri* strains were routinely cultivated in LB medium at 28–30 °C, under continuous agitation (200 rpm). *E. coli* strains were used for cloning purposes or for heterologous expression of recombinant proteins and routinely grown in LB medium at 37 °C, under agitation (200 rpm). TB medium (12 g·L^−1^ triptone, 24 g·L^−1^ yeast extract, 4 g·L^−1^ glycerol, 17 mm KH_2_PO_4_ and 72 mm K_2_HPO_4_) supplemented with kanamycin (35 μg·mL^−1^) was used for protein expression in *E. coli*. Media was supplemented with antibiotics for plasmid selection and maintenance when applicable, using the following concentrations: 100 μg·m^−1^ ampicillin; kanamycin (50 μg·m^−1^); chloramphenicol (34 μg·m^−1^); and streptomycin (50 μg·m^−1^).

*D. discoideum* strain AX2 was routinely grown under axenic conditions using HL5 medium (14.3 g·L^−1^ bacteriological peptone; 7.15 g·L^−1^ yeast extract; 18 g·L^−1^ maltose monohydrate, 0.641 g·L^−1^ Na_2_HPO_4_.2H_2_O and 0.49 g·L^−1^ KH_2_PO_4_, pH 6.5) in bacteriological Petri dishes at 22 °C.

### Cloning and site‐directed mutagenesis

Constructs and primers are listed in Tables S9–S11. For overexpression of different versions of *pknS* under regulation of an arabinose‐inducible promoter in *X. citri*, the corresponding coding regions were amplified from genomic DNA and cloned into the pBRA vector using NheI/SalI restriction sites. Versions of *ecfK* and *pknS* with point mutations were obtained using the QuikChange II XL Site‐Directed Mutagenesis kit (Agilent Technologies, Santa Clara, CA, USA, #200521), following the manufacturer's instructions. The pNIC‐His_6_PknS_1–364_ was used as a template to obtain the pNIC‐His_6_PknS_1–364_M_164_A construct. The construct pBRA‐*ecfK* ^T51A^ was used as a template for generation of the double mutant versions of *ecfK*.

For heterologous expression in *E. coli*, *pknS*_1–364_ and *ecfK* open reading frames were amplified from genomic DNA via PCR and cloned into the expression vector pNIC28‐Bsa4 (GenBank accession no. EF198106), using a ligation‐independent cloning (LIC) method based on annealing of complementary overhangs generated by treatment with T4 DNA polymerase, as previously described in [58, 59, 60, 61, 62]. Primers for PCR amplification included the LIC sequence specific to the target vector. This cloning strategy introduced an amino‐terminal tag of 22 residues (MHHHHHHSSGVDLGTENLYFQ*SM), including a hexahistidine (His6) and a TEV protease cleavage site (marked with *) into the final protein product. Clones were screened by colony PCR, using primers specific to the vector: pLIC‐F and pLIC‐R (Table S11). The resulting PknS and EcfK constructs were transformed into *Escherichia coli* BL21(DE3)‐R3‐pRARE2 and BL21(DE3)‐R3‐lambda‐PPase cells to generate expression clones. All constructs were verified by Sanger DNA sequencing.

### PknS protein production and structure determination

PknS_1–364_ and PknS_1–364_M_164_A were purified from the soluble fraction of *E. coli* cultures. For induction of protein expression, cultures were supplemented with 0.1 mm IPTG and incubated at 18 °C for 16–18 h, with agitation (200 rpm). Cells were collected by centrifugation (15 min, 6222 ***g*** at 4 °C). The pellet was suspended in 2× binding buffer (1 mL per gram of cells) (1× binding buffer is 50 mm HEPES pH 7.5, 0.5 m NaCl, 5.0% (v/v) glycerol, 10 mm imidazole and 1 mm TCEP) supplemented with protease inhibitor cocktail EDTA‐free (Merck Millipore, Burlington, MA, USA; 1 : 200). Cells were stored at −80 °C until use. For protein purification, cells were lysed by sonication (Sonics Vibra Cell VCX750 ultrasonic cell disruptor) on ice for 5 min (5 s on, 10 s off – amplitude = 35%). Polyethyleneimine (PEI – 1 mL of a 5% (w/v) per 40 mL lysate) was added to the cell lysate prior to clarification by centrifugation (60 min, 40 000 ***g***, 4 °C). Recombinant proteins were purified using Ni‐Sepharose resin (HisTrap FF 5 mL, GE Healthcare, Chicago, IL, USA) and eluted stepwise in binding buffer with 300 mm imidazole. Removal of hexahistidine tags was performed at 4 °C overnight using recombinant TEV protease while dialyzing against excess gel filtration buffer [25 mm HEPES, 500 mm NaCl, 0.5 mm TCEP, 5% (v/v) glycerol]. Proteins were further purified by reverse affinity in Ni‐Sepharose followed by gel filtration (Superdex 200 16/60, GE Healthcare). The expected molecular mass was obtained following LC‐MS analysis of purified PknS (39.6 kDa for the wild‐type and 39.5 kDa for the M164A mutant).

For protein crystallization, PknS_1–364_M_164_A in gel filtration buffer was concentrated to 12.4 mg·mL^−1^ using 30 kDa MWCO centrifugal concentrators (Millipore) at 4 °C. CHIR‐124 (Selleckchem Chemicals LLC, Houston, TX, EUA) in 100% DMSO was added to the protein solution at threefold molar excess and incubated on ice for approximately 30 min. This mixture was centrifuged at 21 000 ***g*** for 10 min at 4 °C prior to setting up 150 nL volume sitting drops at three ratios of protein–inhibitor complex to reservoir solution (2 : 1, 1 : 1, or 1 : 2). Crystallization experiments were performed at 20 °C. Crystals were cryoprotected in reservoir solution supplemented with 20–25% glycerol before flash‐freezing in liquid nitrogen for data collection. Diffraction data were collected at the Diamond Light Source (DLS, beamline I‐24). The best‐diffracting crystals grew under the conditions described in Table S2. Crystal optimization used Newman's buffer system [63].

To determine and refine the crystal structure, we processed the diffraction images with XDS [64] and applied scaling corrections using AIMLESS from the CCP4 package [65]. Phaser [66] was then used to carry out molecular replacement, with the *M. tuberculosis* cPknF structure (PDB 7NAA) [67] serving as the template. Model adjustments were carried out manually in real space with Coot [68] and subsequent reciprocal‐space refinement employed REFMAC5 as implemented in CCP4 [69]. The ligand CHIR‐124 was modeled at full occupancy (1.0). Overall stereochemical quality was evaluated using MolProbity [70]. A summary of data processing and refinement statistics is provided in Table S2. Coordinates and structure factors have been deposited in the PDB under accession code 9EED. An evaluation of the AlphaFold3‐predicted PknS model shown in Fig. S3 is presented in Fig. S10.

### Purification of soluble EcfK from inclusion bodies

Recombinant production of EcfK in *E. coli* followed a slightly different procedure than the one described above for PknS because EcfK accumulated in inclusion bodies. For that, cultures (OD600 ∼ 0.5) were induced with 0.1 mm IPTG for 2.5 h at 37 °C and cells were collected by centrifugation (15 min, 5000 rpm at 4 °C). The cell pellet was suspended in Inclusion Bodies Resuspension Buffer (25 mL per liter of culture) (20 mm Tris/HCl pH 8.0). Cells were stored at −80 °C until use.

To isolate inclusion bodies containing EcfK, cells were first lysed by sonication and the cell lysate clarified by centrifugation as described above. Following centrifugation, the cell pellet was suspended in cold Inclusion Bodies Wash Buffer (25 mL per liter of culture) (2 m Urea, 20 mm Tris pH 8.0, 0.5 m NaCl, 1% Triton X‐100). The mixture was sonicated (Sonics Vibra Cell VCX750 ultrasonic cell disrupter) on ice for 2 min (30 s on, 30 s off—amplitude = 35%), and clarified by centrifugation (10 min, 15 000 rpm, 4 °C). This process was repeated five times and the final pellet containing the inclusion bodies was washed one more time with Inclusion Bodies Resuspension Buffer and the supernatant was discarded. Inclusion bodies were flash frozen in liquid nitrogen and stored at −80 °C until use.

To purify soluble EcfK, inclusion bodies were resuspended in Denaturing Buffer (10 mL per liter of culture) (6 m guanidine‐HCl, 0.5 m NaCl, 20 mm Tris/HCl pH 8.0, 5 mm imidazole, 1 mm TCEP, 0.1 mm EDTA) and stirred 2 h at room temperature. The mixture was centrifuged (21 130 ***g*** for 15 min at 4 °C) and the supernatant was filtered through a 0.22 μm filter (Millipore). This mixture was applied onto a nickel resin (~ 2 mL of slurry) packed into an Econo‐Pac chromatography column (Bio‐Rad, Hercules, CA, USA) previously equilibrated with 5 column volumes (CV) of Denaturing Buffer (6 m guanidine‐HCl, 0.5 m NaCl, 20 mm Tris/HCl pH 8.0, 5 mm imidazole, 1 mm TCEP, 0.1 mm EDTA). Unbound proteins were washed off with 10 CV of Denaturing Wash Buffer (6 m Urea, 0.5 m NaCl, 20 mm Tris/HCl pH 8.0, 20 mm imidazole, 1 mm TCEP). Urea was removed in a stepwise manner (each step consisting of 5 CV and a 1 m drop in urea concentration) using Refolding Buffer (0.5 m NaCl, 50 mm Hepes pH 7.5, 20 mm imidazole, 1 mm TCEP, 5% glycerol). The column was washed one more time with 5 CV of Refolding Buffer. To elute soluble EcfK, 2.5 CV of Elution buffer was applied onto the column and the eluate was collected in a tube containing 1 mL of Refolding Buffer complemented with 20% glycerol. Soluble EcfK was further purified by size exclusion chromatography (Superdex 200 16/60, GE Healthcare) in gel filtration buffer [25 mm HEPES, 500 mm NaCl, 0.5 mm TCEP, 5% (v/v) glycerol]. The expected molecular mass was obtained following LC‐MS analysis of purified EcfK (22.4 kDa).

### Thermal shift assays

Assays were performed with 2.0 μm of purified PknS_1–364_ and PknS_1–364_M_164_A in gel filtration buffer with 1000‐fold diluted SYPRO orange dye (Applied Biosystems, Thermo Scientific, Waltham, MA, USA). A liquid handling robot (Felix‐CyBio) was used to add the compounds from SelleckChem library (20 nL) to each well of a 384‐well plate containing the protein of interest. Temperature denaturation was performed in a QuantStudio6 real‐time PCR machine (Applied Biosystems). Data were analyzed with Protein Thermal Shift Software v1.3 (Thermo Fischer Scientific).

### Computational analysis of protein–protein interactions mediated by sigma factors within transcription initiation complexes

All predicted structures were obtained using AlphaFold 3 as implemented in www.alphafoldserver.com [42]. Per‐residue confidence scores (pLDDT) and Predicted Aligned Error (PAE) heatmaps are presented in Figs S11–S19.

Holo‐RNA Polymerase complexes for *X. citri* pv. *citri* 306 were composed of two copies of DNA‐directed RNA polymerase alpha subunit (RpoA; KEGG Entry: XAC0996; UNIPROT ID: P0A0Y2), and one copy each of: DNA‐directed RNA polymerase subunit beta (RpoB; KEGG Entry: XAC0965; UNIPROT ID: Q8PNT0); DNA‐directed RNA polymerase subunit beta' (RpoC; KEGG Entry: XAC0966; UNIPROT ID: Q8PNS9); and DNA‐directed RNA polymerase subunit omega (RpoZ; KEGG Entry: XAC3394; UNIPROT ID: P66732). Nucleic acids within the complexes were as follows: one copy of double‐stranded DNA (5′ CTGCATCCGTGAGTCGAGGGTGTTCAATAATTAGCACTAAAAGTTCCG 3′) and one copy of single‐stranded RNA (5′ CUCGA 3′) derived from the CryoEM structure of *E. coli* σ^E^ transcription initiation complex (PDB ID: 6JBQ) [34]. Sigma factors used to build individual complexes were: EcfK (KEGG Entry: XAC4128; UNIPROT ID: A0AAI7ZIW4); RfaY (KEGG Entry: XAC2814; UNIPROT ID: A0AAI7ZGL0); PrtI (KEGG Entry: XAC3989; UNIPROT ID: A0AAI7ZIN4); RpoE (KEGG Entry: XAC1380 UNIPROT ID: A0AAI8ER26); XAC0922 (KEGG Entry: XAC0922; UNIPROT ID: A0AAI7ZDN4); XAC1682 (KEGG Entry: XAC1682; UNIPROT ID: A0AAI7ZET4); XAC2191 (KEGG Entry: XAC2191; UNIPROT ID: A0AAI7ZFJ4); and AlgU (KEGG Entry: XAC1319; UNIPROT ID: A0AAI8ES64).

Holo‐RNA Polymerase complexes for *V. parahaemolyticus* RIMD 2210633 were composed of two copies of DNA‐directed RNA polymerase alpha subunit (RpoA; KEGG Entry: VP0282; UNIPROT ID: Q87SZ0), and one copy each of: DNA‐directed RNA polymerase subunit beta (RpoB; KEGG Entry: VP2922; UNIPROT ID: Q87KQ4); DNA‐directed RNA polymerase subunit beta’ (RpoC; KEGG Entry: VP2921; UNIPROT ID: Q87KQ5); and DNA‐directed RNA polymerase subunit omega (RpoZ; KEGG Entry: VP0160; UNIPROT ID: Q87TB0). As above, the nucleic acids within the complexes were as follows: one copy of double‐stranded DNA (5′ CTGCATCCGTGAGTCGAGGGTGTTCAATAATTAGCACTAAAAGTTCCG 3′) and one copy of single‐stranded RNA (5′ CUCGA 3′) derived from the CryoEM structure of *E. coli* σ^E^ transcription initiation complex (PDB ID 6JBQ). *V. parahaemolyticus* sigma factor used was as follows: VP0055 (KEGG Entry: VP0055; UNIPROT ID: Q87TL4).

### *In vitro* phosphorylation assays

Proteins were incubated at room temperature for 16 h in kinase buffer (50 mm HEPES pH 7.5, 0.5 mm TCEP, 5 mm MnCl_2_) with and without addition of 1 mm ATP. Reactions were inactivated by addition of 0.1% formic acid, and samples were analyzed by LC‐MS/MS for intact mass determination. Transphosphorylation assays were performed by co‐incubation of PknS_1–364_ (5.6 μm) and EcfK (11.5 μm).

### Phosphosite identification by mass spectrometry

The *in vitro* phosphorylation assay was performed as described above, except for a 2‐h incubation time. Samples were processed as previously described [71]. Briefly, following the reaction, samples were exchanged into 50 mm ammonium bicarbonate and incubated with 25 μL of RapiGest SF (0.2%; Waters Corp., Milford, MA, USA; catalog #186001861) for 15 min at 80 °C. The solution was then supplemented with DTT to a final concentration of 4 mm and heated for 30 min at 60 °C. Alkylation was performed by adding iodoacetamide to a final concentration of 12 mm, followed by a 30‐min incubation protected from light. Trypsin (Promega, Fitchburg, WI, USA; catalog #V511A), prepared in 50 mm ammonium bicarbonate, was added at a 1 : 100 enzyme‐to‐substrate mass ratio, and digestion proceeded for 16 h at 37 °C with gentle agitation. RapiGest was subsequently cleaved by adding trifluoroacetic acid (TFA; Pierce, Waltham, MA, USA; catalog #53102) and incubating the mixture for 90 min at 37 °C. Samples were centrifuged at 14 000 rpm for 30 min at 6 °C, and the clarified supernatant was transferred to a clean microcentrifuge tube (Axygen, Union City, CA, USA) for LC‐MS/MS analysis.

### Mass spectrometry analysis

For intact mass analysis, samples were analyzed as previously described [71]. For intact mass measurements, protein samples were examined by reverse‐phase HPLC coupled to ESI‐QToF mass spectrometry in positive ion mode using an Acquity H‐class HPLC system interfaced with an XEVO G2 Xs Q‐ToF instrument (Waters Corp.). Approximately 0.5 μL of each sample (~ 12.5 ng), prepared in Solvent A (0.1% formic acid in water), was injected onto a C4 analytical column (ACQUITY UPLC Protein BEH C4, 300 Å, 1.7 μm, 2.1 × 100 mm; Waters Corp.) maintained at 45 °C. Protein was eluted with a 4‐min linear gradient from 10 to 90% Solvent B (0.1% formic acid in acetonitrile). After each run, the column was regenerated with 90% Solvent B for 90 s and returned to starting conditions (10% B) over 210 s. The loading flow rate was 0.5 μL·min^−1^, while washing and elution were carried out at 400 μL·min^−1^. Leu‐enkephalin (556.2771 Da) served as the lock‐mass reference, recorded with a 0.5‐s scan time and a ± 0.5 Da mass window. Time‐of‐flight spectra were acquired between 100 and 2000 Da at 1 s per scan, with the ESI cone voltage held at 40 V.

For phosphopeptide mapping, digested samples were evaluated using a low‐flow LC‐ESI‐MS/MS configuration equipped with a Waters low‐flow probe (ref. #186007529), an Acquity M‐class UPLC, and an XEVO G2 Xs Q‐ToF mass spectrometer. Ten microliters of sample, in 99% Solvent A and 1% Solvent B, were loaded onto a trap column (Symmetry C18, 100 Å, 5 μm, 180 μm × 20 mm; Waters Corp.) held at 40 °C with a 15 μL·min^−1^ flow for 4 min. Peptides were subsequently transferred to an HSS C18 T3 analytical column (300 μm × 150 mm, 1.8 μm), also maintained at 40 °C. Chromatographic separation was performed at 5 μL·min^−1^ over a 30.11‐min gradient: 7–40% Solvent B from 0 to 27.99 min, 40–85% from 27.99 to 30.11 min, a hold at 85% B from 30.11 to 34.35 min, re‐equilibration to 7% B from 34.35 to 36.47 min, and a final hold at 7% B until 57.68 min.

The nano‐ESI source settings included a capillary voltage of 2.8 kV, sampling cone at 30 V, source offset at 80 V, a source temperature of 80 °C, and a desolvation gas flow of 600 L h^−1^. Data were recorded in positive ion, sensitivity‐optimized mode at 0.5 scans s^−1^ across *m/z* 50–2000. Data‐independent acquisition was used, with the low‐energy channel set to 0 eV and the high‐energy fragmentation channel applying a 15–45 eV collision‐energy ramp. Leu‐enkephalin (556.2771 Da, 2+), at 100 pM in 40 : 60 ACN/H₂O with 0.1% FA, was infused through the lock‐spray at 3 μL·min^−1^ for mass‐accuracy monitoring. The lock‐spray capillary voltage was 3.1 kV, and lock‐mass spectra were acquired every 30 s at 0.5 scans s^−1^, although the correction was not applied in real time.

### MS data analysis

Raw MS data files were examined in MassLynx v4.1 and deconvoluted for intact mass determination using the MaxEnt 1 algorithm (both from Waters Corp.), as described before [71]. Processing of proteomics data was carried out in ProteinLynx Global Server 3.0.3 (PLGS, Waters Corp.) using a two‐stage workflow. In the first stage, spectra were extracted with the following settings: lock‐mass correction using the doubly charged Leu‐enkephalin ion (*m/z* 556.2771) with a 0.4‐Da window; a 500‐count threshold for low‐energy spectra; a 50‐count threshold for elevated‐energy spectra; and automatic assignment of chromatographic peak width and MS‐ToF resolution. In the second stage, the extracted spectra were searched against the *Xanthomonas axonopodis* UniProt (Swiss‐Prot/TrEMBL) database released in February 2024. Database‐search parameters included automatic peptide and fragment mass tolerances, a minimum of three fragment‐ion matches per peptide and five per protein, and at least one peptide required for protein identification. Searches allowed one missed tryptic cleavage, with carbamidomethylation of cysteine specified as a fixed modification and methionine oxidation plus Ser/Thr/Tyr phosphorylation defined as variable modifications. A reversed‐sequence decoy database generated within PLGS was used to estimate peptide and protein false discovery rates. Protein identifications were accepted only if they met a confidence threshold greater than 95%.

### Amoeba grazing assays

To assess the virulence of *X. citri* strains to *D. discoideum* amoebae, we scored the number of amoebal plaques formed on bacterial lawns. We based our method on previously established protocols, with modifications [14, 41, 72]. Bacteria were cultured overnight in LB medium supplemented with streptomycin. Xanthan gum was removed after centrifugation, and the cell pellet was resuspended in SorC buffer (Sorensen phosphate buffer pH 6, supplemented with 50 μm CaCl_2_) (DictyBase Stock Center recipe) and the OD_600nm_ was adjusted to 3 in 500 μL. *D. discoideum* cells were kept in HL5c medium with glucose (Formedium, Swaffham, Norfolk, UK) until ≈ 80–90% confluence. Cells were detached from the culture dish, resuspended in fresh HL5c medium, collected by gentle centrifugation (5 min; 1500× g), and resuspended in SorC buffer. Cell numbers were determined using a Neubauer chamber, and the amoebal concentration was adjusted to 5 × 10^4^ cells mL^−1^. About 10 μL of this suspension (corresponding to 500 amoebal cells) were mixed with 500 μL of bacterial suspension. Half of the mix was gently spread onto two replicate plates with N agar medium (1 g peptone, 1 g glucose, 20 g agar in 1 liter of Sorensen phosphate buffer pH 6) [73]. Plates were wrapped in aluminum foil and incubated at 22 °C for 7 days. *D. discoideum* plaques were scored starting on the third day until the seventh day. The plaque numbers obtained on the lawn of the T6SS mutant strain (Δ*ecfK* pBRA) was set to 100% in each replicate. The plaque numbers obtained for the other strains are shown relative to the numbers obtained in the T6SS mutant strain. Four biologically independent experiments were performed. The individual experimental data points (corresponding to the means of technical replicates) are shown in the graph. Statistical significance was determined by one‐way ANOVA (*P* value < 0.0001) followed by a Dunnett's multiple comparison test (alpha of 0.05).

### Immunoblot analysis

For *in vivo* analysis of PknS phosphorylation in *X. citri*, saturated cultures of the strains of interest carrying pBRA with *pknS* versions were normalized to OD_600nm_ = 0.1 and incubated until exponential growth phase (OD_600nm_ = 0.5). Arabinose (0.3%) was added, and cultures were incubated for 2 h at 28 °C to induce expression of *pknS* from the pBRA vector. Cells were harvested by centrifugation and lysed by direct resuspension in SDS/PAGE sample buffer (60% glycerol, 1 M Tris/HCl pH 6.8, 12% SDS, 0.1% β‐mercaptoethanol, 0.4% bromophenol blue). To normalize protein samples, the volume of SDS/PAGE sample buffer added was adjusted according to the OD_600nm_ of cultures.

Protein samples were separated in 10% or 12.5% SDS/PAGE and transferred to PVDF membranes. The anti‐FLAG antibody was used at 1 : 20 000 (ANTI‐FLAG M2 monoclonal, Sigma‐Aldrich) and membranes were incubated for 1 h at room temperature. To detect PknS phosphorylation, Phospho‐Threonine Antibody (P‐Thr‐Polyclonal #9381, Cell Signalling, Danvers, MA, USA) was used at 1 : 1000, followed by incubation at 4 °C for16 h.

## Conflict of interest

The authors declare no conflicts of interest.

## Author contributions

LPL planned and performed experiments and analyzed data; DS, DLS, BWM and NDD performed experiments and analyzed data; EBSM, CVR, RFL, JFTSS, and AS performed experiments; KBM supervised work and analyzed data; RMC and CEAM performed experiments, analyzed data, wrote the paper, and supervised work.

## Supporting information


**Fig. S1.** Analysis of PknS_1–364_ purification and autophosphorylation.
**Fig. S2.** Functionality of Flag‐tagged versions of PknS in *X. citri*, evaluated by ability to complement the phenotype of the Δ*pknS* strain and restore the resistance to amoeba predation.
**Fig. S3.** Crystal packing for PknS_1–364_M_164_A:CHIR‐124 co‐crystals and predicted structures of residues 1–81 in PknS.
**Fig S4.** His_6_‐EcfK purification.
**Fig. S5.** Mass spectra (A) and deconvoluted mass spectra (B) obtained by LC/MS analysis of His_6_‐EcfK incubated with ATP and PknS_1‐364_.
**Fig. S6.** Mass spectra (A) and deconvoluted mass spectra (B) of His_6_‐EcfK with ATP, as determined by LC/MS analysis.
**Fig. S7.** Mass spectra of phosphorylated peptides of His_6_‐EcfK identified by LC‐MS/MS.
**Fig. S8.** Results of individual plaque assay experiments, which were combined and displayed as relative values in Fig. 5D.
**Fig. S9.** Detailed views of potential contacts and charge complementarity between *X. citri* RpoC and the selected ECF sigma factors.
**Fig. S10.** Quality assessment of AlphaFold3‐predicted structures of PknS shown in Fig. S3.
**Fig. S11.** Quality assessment of the AlphaFold3‐predicted structure of Holo‐RNA Polymerase complexes for *X. citri* pv. *citri* 306 bound to sigma factor EcfK (XAC4128).
**Fig. S12.** Quality assessment of the AlphaFold3‐predicted structure of Holo‐RNA Polymerase complexes for *X. citri* pv. *citri* 306 bound to sigma factor RfaY (XAC2814).
**Fig. S13.** Quality assessment of the AlphaFold3‐predicted structure of Holo‐RNA Polymerase complexes for *X. citri* pv. *citri* 306 bound to sigma factor PrtI (XAC3989).
**Fig. S14.** Quality assessment of the AlphaFold3‐predicted structure of Holo‐RNA Polymerase complexes for *X. citri* pv. *citri* 306 bound to sigma factor RpoE (XAC1380).
**Fig. S15.** Quality assessment of the AlphaFold3‐predicted structure of Holo‐RNA Polymerase complexes for *X. citri* pv. *citri* 306 bound to sigma factor XAC0922.
**Fig. S16.** Quality assessment of the AlphaFold3‐predicted structure of Holo‐RNA Polymerase complexes for *X. citri* pv. *citri* 306 bound to sigma factor XAC2191.
**Fig. S17.** Quality assessment of the AlphaFold3‐predicted structure of Holo‐RNA Polymerase complexes for *X. citri* pv. *citri* 306 bound to sigma factor AlgU (XAC1319).
**Fig. S18.** Quality assessment of the AlphaFold3‐predicted structure of Holo‐RNA Polymerase complexes for *X. citri* pv. *citri* 306 bound to sigma factor XAC1682.
**Fig. S19.** Quality assessment of the AlphaFold3‐predicted structure of Holo‐RNA Polymerase complexes for *V. parahaemolyticus* RIMD 2210633.
**Table S1.** Thermal shift data for all compounds of the Selleckchem library.
**Table S2.** Data collection and Refinement Statistics.
**Table S3.** Phosphorylated peptides of His_6_‐EcfK identified by LC‐MS/MS.
**Table S4.** His_6_‐EcfK peptides identified by LC‐MS/MS.
**Table S5.** Mass spectrometry raw data for His_6_‐EcfK.
**Table S6.** Amino acid frequencies in all ECF43.
**Table S7.** Amino acid frequencies in Xanthomonadales´ ECF43.
**Table S8.** Strains used in this study.
**Table S9.** Plasmids used in this study.
**Table S10.** Constructs for heterologous expression in *E. coli*.
**Table S11.** Oligonucleotides used in this study.

## Data Availability

PknS protein structure has been deposited in Protein Data Bank (PDB ID: 9EED), data collection and refinement statistics are presented in Table S2. Computer generated structures are publicly available at ModelArchive (https://www.modelarchive.org/) under the following entries: ma‐dz1ku (XAC4127‐PknS; Fig. S10); ma‐dzkrz (XAC4128; Fig. S11); ma‐hli0b (XAC2814; Fig. S12); ma‐8p4ct (XAC3989; Fig. S13); ma‐mtt3k (XAC1380; Fig. S14); ma‐wli7x (XAC0922; Fig. S15); ma‐kfrzg (XAC2191; Fig. S16); ma‐omv7d (XAC1319; Fig. S17); ma‐nq0bu (XAC1682; Fig. S18); ma‐wv6wh (Vibrio; Fig. S19). The mass spectrometry data that support the findings of this study are available in (Tables S3–S5 and Figs S4–S7).

## References

[1] Hanks SK & Hunter T (1995) Protein kinases 6. The eukaryotic protein kinase superfamily: kinase (catalytic) domain structure and classification. FASEB J 9, 576–596. 7768349

[2] Mijakovic I , Grangeasse C & Turgay K (2016) Exploring the diversity of protein modifications: special bacterial phosphorylation systems. FEMS Microbiol Rev 40, 398–417. 26926353 10.1093/femsre/fuw003

[3] Stock AM , Robinson VL & Goudreau PN (2000) Two‐component signal transduction. Annu Rev Biochem 69, 183–215. 10966457 10.1146/annurev.biochem.69.1.183

[4] Nagarajan SN , Lenoir C & Grangeasse C (2022) Recent advances in bacterial signaling by serine/threonine protein kinases. Trends Microbiol 30, 553–566. 34836791 10.1016/j.tim.2021.11.005

[5] Frando A & Grundner C (2024) More than two components: complexities in bacterial phosphosignaling. mSystems 9, e0028924. 38591891 10.1128/msystems.00289-24 PMC11097640

[6] Frando A , Boradia V , Gritsenko M , Beltejar C , Day L , Sherman DR , Ma S , Jacobs JM & Grundner C (2023) The mycobacterium tuberculosis protein O‐phosphorylation landscape. Nat Microbiol 8, 548–561. 36690861 10.1038/s41564-022-01313-7 PMC11376436

[7] Barthe P , Mukamolova GV , Roumestand C & Cohen‐Gonsaud M (2010) The structure of PknB extracellular PASTA domain from mycobacterium tuberculosis suggests a ligand‐dependent kinase activation. Structure 18, 606–615. 20462494 10.1016/j.str.2010.02.013

[8] Hsu F , Schwarz S & Mougous JD (2009) TagR promotes PpkA‐catalysed type VI secretion activation in *Pseudomonas aeruginosa* . Mol Microbiol 72, 1111–1125. 19400797 10.1111/j.1365-2958.2009.06701.x PMC3402362

[9] Leyns F , De Cleene M , Swings J‐G & De Ley J (1984) The host range of the genus *Xanthomonas* . Bot Rev 50, 308–356.

[10] Alvarez‐Martinez CE , Sgro GG , Araujo GG , Paiva MRN , Matsuyama BY , Guzzo CR , Andrade MO & Farah CS (2021) Secrete or perish: the role of secretion systems in biology. Comput Struct Biotechnol J 19, 279–302. 33425257 10.1016/j.csbj.2020.12.020 PMC7777525

[11] Souza DP , Oka GU , Alvarez‐Martinez CE , Bisson‐Filho AW , Dunger G , Hobeika L , Cavalcante NS , Alegria MC , Barbosa LRS , Salinas RK *et al*. (2015) Bacterial killing via a type IV secretion system. Nat Commun 6, 6453. 25743609 10.1038/ncomms7453

[12] White FF , Potnis N , Jones JB & Koebnik R (2009) The type III effectors of *Xanthomonas* . Mol Plant Pathol 10, 749–766. 19849782 10.1111/j.1364-3703.2009.00590.x PMC6640274

[13] Baptista JC , Machado MA , Homem RA , Torres PS , Vojnov AA & do Amaral AM (2010) Mutation in the xpsD gene of *Xanthomonas axonopodis* pv. *citri* affects cellulose degradation and virulence. Genet Mol Biol 33, 146–153. 21637619 10.1590/S1415-47572009005000110 PMC3036071

[14] Bayer‐Santos E , Lima LDP , Ceseti L d M , Ratagami CY , de Santana ES , da Silva AM , Farah CS & Alvarez‐Martinez CE (2018) Xanthomonas *citri* T6SS mediates resistance to *Dictyostelium* predation and is regulated by an ECF σ factor and cognate Ser/Thr kinase. Environ Microbiol 20, 1562–1575. 29488354 10.1111/1462-2920.14085

[15] Bayer‐Santos E , Ceseti LDM , Farah CS & Alvarez‐Martinez CE (2019) Distribution, function and regulation of type 6 secretion systems of Xanthomonadales. Front Microbiol 10, 1635. 31379785 10.3389/fmicb.2019.01635 PMC6653060

[16] Mascher T (2013) Signaling diversity and evolution of extracytoplasmic function (ECF) σ factors. Curr Opin Microbiol 16, 148–155. 23466210 10.1016/j.mib.2013.02.001

[17] Casas‐Pastor D , Müller RR , Jaenicke S , Brinkrolf K , Becker A , Buttner MJ , Gross CA , Mascher T , Goesmann A & Fritz G (2021) Expansion and re‐classification of the extracytoplasmic function (ECF) σ factor family. Nucleic Acids Res 49, 986–1005. 33398323 10.1093/nar/gkaa1229 PMC7826278

[18] Iyer SC , Casas‐Pastor D , Kraus D , Mann P , Schirner K , Glatter T , Fritz G & Ringgaard S (2020) Transcriptional regulation by σ factor phosphorylation in bacteria. Nat Microbiol 5, 395–406. 31988380 10.1038/s41564-019-0648-6

[19] Kaur P , Rausch M , Malakar B , Watson U , Damle NP , Chawla Y , Srinivasan S , Sharma K , Schneider T , Jhingan GD *et al*. (2019) LipidII interaction with specific residues of *Mycobacterium tuberculosis* PknB extracytoplasmic domain governs its optimal activation. Nat Commun 10, 1231. 30874556 10.1038/s41467-019-09223-9 PMC6428115

[20] Mir M , Asong J , Li X , Cardot J , Boons G‐J & Husson RN (2011) The extracytoplasmic domain of the *Mycobacterium tuberculosis* Ser/Thr kinase PknB binds specific muropeptides and is required for PknB localization. PLoS Pathog 7, e1002182. 21829358 10.1371/journal.ppat.1002182 PMC3145798

[21] Greenstein AE , Echols N , Lombana TN , King DS & Alber T (2007) Allosteric activation by dimerization of the PknD receptor Ser/Thr protein kinase from *Mycobacterium tuberculosis* . J Biol Chem 282, 11427–11435. 17242402 10.1074/jbc.M610193200

[22] Froquet R , Lelong E , Marchetti A & Cosson P (2009) *Dictyostelium discoideum*: a model host to measure bacterial virulence. Nat Protoc 4, 25–30. 19131953 10.1038/nprot.2008.212

[23] Tse AN , Rendahl KG , Sheikh T , Cheema H , Aardalen K , Embry M , Ma S , Moler EJ , Ni ZJ , de Lopes Menezes DE *et al*. (2007) CHIR‐124, a novel potent inhibitor of Chk1, potentiates the cytotoxicity of topoisomerase I poisons in vitro and in vivo. Clin Cancer Res 13, 591–602. 17255282 10.1158/1078-0432.CCR-06-1424

[24] Ni Z‐J , Barsanti P , Brammeier N , Diebes A , Poon DJ , Ng S , Pecchi S , Pfister K , Renhowe PA , Ramurthy S *et al*. (2006) 4‐(Aminoalkylamino)‐3‐benzimidazole‐quinolinones as potent CHK‐1 inhibitors. Bioorg Med Chem Lett 16, 3121–3124. 16603354 10.1016/j.bmcl.2006.03.059

[25] Fedorov O , Marsden B , Pogacic V , Rellos P , Müller S , Bullock AN , Schwaller J , Sundström M & Knapp S (2007) A systematic interaction map of validated kinase inhibitors with Ser/Thr kinases. Proc Natl Acad Sci USA 104, 20523–20528. 18077363 10.1073/pnas.0708800104 PMC2154464

[26] Niesen FH , Berglund H & Vedadi M (2007) The use of differential scanning fluorimetry to detect ligand interactions that promote protein stability. Nat Protoc 2, 2212–2221. 17853878 10.1038/nprot.2007.321

[27] Jumper J , Evans R , Pritzel A , Green T , Figurnov M , Ronneberger O , Tunyasuvunakool K , Bates R , Žídek A , Potapenko A *et al*. (2021) Highly accurate protein structure prediction with AlphaFold. Nature 596, 583–589. 34265844 10.1038/s41586-021-03819-2 PMC8371605

[28] Krissinel E & Henrick K (2004) Secondary‐structure matching (SSM), a new tool for fast protein structure alignment in three dimensions. Acta Crystallogr D Biol Crystallogr 60, 2256–2268. 15572779 10.1107/S0907444904026460

[29] Zuccotto F , Ardini E , Casale E & Angiolini M (2010) Through the “gatekeeper door”: exploiting the active kinase conformation. J Med Chem 53, 2681–2694. 20000735 10.1021/jm901443h

[30] Levitzki A , Gazit A , Osherov N , Posner I & Gilon C (1991) Inhibition of protein‐tyrosine kinases by tyrphostins. Methods Enzymol 201, 347–361. 1658552 10.1016/0076-6879(91)01031-v

[31] Regan J , Pargellis CA , Cirillo PF , Gilmore T , Hickey ER , Peet GW , Proto A , Swinamer A & Moss N (2003) The kinetics of binding to p38MAP kinase by analogues of BIRB 796. Bioorg Med Chem Lett 13, 3101–3104. 12941343 10.1016/s0960-894x(03)00656-5

[32] Pargellis C , Tong L , Churchill L , Cirillo PF , Gilmore T , Graham AG , Grob PM , Hickey ER , Moss N , Pav S *et al*. (2002) Inhibition of p38 MAP kinase by utilizing a novel allosteric binding site. Nat Struct Biol 9, 268–272. 11896401 10.1038/nsb770

[33] Feklístov A , Sharon BD , Darst SA & Gross CA (2014) Bacterial sigma factors: a historical, structural, and genomic perspective. Ann Rev Microbiol 68, 357–376. 25002089 10.1146/annurev-micro-092412-155737

[34] Fang C , Li L , Shen L , Shi J , Wang S , Feng Y & Zhang Y (2019) Structures and mechanism of transcription initiation by bacterial ECF factors. Nucleic Acids Res 47, 7094–7104. 31131408 10.1093/nar/gkz470 PMC6648896

[35] Lin W , Mandal S , Degen D , Cho MS , Feng Y , Das K & Ebright RH (2019) Structural basis of ECF‐σ‐factor‐dependent transcription initiation. Nat Commun 10, 710. 30755604 10.1038/s41467-019-08443-3 PMC6372665

[36] Li L , Fang C , Zhuang N , Wang T & Zhang Y (2019) Structural basis for transcription initiation by bacterial ECF σ factors. Nat Commun 10, 1153. 30858373 10.1038/s41467-019-09096-y PMC6411747

[37] Zuo Y & Steitz TA (2015) Crystal structures of the *E. coli* transcription initiation complexes with a complete bubble. Mol Cell 58, 534–540. 25866247 10.1016/j.molcel.2015.03.010 PMC5567806

[38] Murakami KS , Masuda S & Darst SA (2002) Structural basis of transcription initiation: RNA polymerase holoenzyme at 4 A resolution. Science 296, 1280–1284. 12016306 10.1126/science.1069594

[39] Joo DM , Ng N & Calendar R (1997) A sigma32 mutant with a single amino acid change in the highly conserved region 2.2 exhibits reduced core RNA polymerase affinity. Proc Natl Acad Sci USA 94, 4907–4912. 9144163 10.1073/pnas.94.10.4907 PMC24604

[40] Sharp MM , Chan CL , Lu CZ , Marr MT , Nechaev S , Merritt EW , Severinov K , Roberts JW & Gross CA (1999) The interface of sigma with core RNA polymerase is extensive, conserved, and functionally specialized. Genes Dev 13, 3015–3026. 10580008 10.1101/gad.13.22.3015 PMC317155

[41] Pukatzki S , Ma AT , Sturtevant D , Krastins B , Sarracino D , Nelson WC , Heidelberg JF & Mekalanos JJ (2006) Identification of a conserved bacterial protein secretion system in *Vibrio cholerae* using the *Dictyostelium* host model system. Proc Natl Acad Sci USA 103, 1528–1533. 16432199 10.1073/pnas.0510322103 PMC1345711

[42] Abramson J , Adler J , Dunger J , Evans R , Green T , Pritzel A , Ronneberger O , Willmore L , Ballard AJ , Bambrick J *et al*. (2024) Accurate structure prediction of biomolecular interactions with AlphaFold 3. Nature 630, 493–500. 38718835 10.1038/s41586-024-07487-w PMC11168924

[43] You L , Shi J , Shen L , Li L , Fang C , Yu C , Cheng W , Feng Y & Zhang Y (2019) Structural basis for transcription antitermination at bacterial intrinsic terminator. Nat Commun 10, 3048. 31296855 10.1038/s41467-019-10955-x PMC6624301

[44] Wen A , Zhao M , Jin S , Lu Y‐Q & Feng Y (2022) Structural basis of AlpA‐dependent transcription antitermination. Nucleic Acids Res 50, 8321–8330. 35871295 10.1093/nar/gkac608 PMC9371919

[45] Mougous JD , Gifford CA , Ramsdell TL & Mekalanos JJ (2007) Threonine phosphorylation post‐translationally regulates protein secretion in *Pseudomonas aeruginosa* . Nat Cell Biol 9, 797–803. 17558395 10.1038/ncb1605

[46] Teper D , Girija AM , Bosis E , Popov G , Savidor A & Sessa G (2018) The *Xanthomonas euvesicatoria* type III effector XopAU is an active protein kinase that manipulates plant MAP kinase signaling. PLoS Pathog 14, e1006880. 29377937 10.1371/journal.ppat.1006880 PMC5805367

[47] Zhou X , Teper D , Andrade MO , Zhang T , Chen S , Song W‐Y & Wang N (2018) A phosphorylation switch on Lon protease regulates bacterial type III secretion system in host. MBio 9, 10–1128. 10.1128/mBio.02146-17 PMC5784255 29362236

[48] Prisic S & Husson RN (2014) *Mycobacterium tuberculosis* serine/threonine protein kinases. Microbiol Spectr 2, 681–708. 10.1128/microbiolspec.MGM2-0006-2013 PMC4242435 25429354

[49] Pompeo F , Byrne D , Mengin‐Lecreulx D & Galinier A (2018) Dual regulation of activity and intracellular localization of the PASTA kinase PrkC during Bacillus subtilis growth. Sci Rep 8, 1660. 29374241 10.1038/s41598-018-20145-2 PMC5786024

[50] Zucchini L , Mercy C , Garcia PS , Cluzel C , Gueguen‐Chaignon V , Galisson F , Freton C , Guiral S , Brochier‐Armanet C , Gouet P *et al*. (2018) PASTA repeats of the protein kinase StkP interconnect cell constriction and separation of *Streptococcus pneumoniae* . Nat Microbiol 3, 197–209. 29203882 10.1038/s41564-017-0069-3

[51] Holmberg CI , Tran SEF , Eriksson JE & Sistonen L (2002) Multisite phosphorylation provides sophisticated regulation of transcription factors. Trends Biochem Sci 27, 619–627. 12468231 10.1016/s0968-0004(02)02207-7

[52] Schweer J , Türkeri H , Kolpack A & Link G (2010) Role and regulation of plastid sigma factors and their functional interactors during chloroplast transcription – recent lessons from Arabidopsis thaliana. Eur J Cell Biol 89, 940–946. 20701995 10.1016/j.ejcb.2010.06.016

[53] Schweer J , Türkeri H , Link B & Link G (2010) AtSIG6, a plastid sigma factor from *Arabidopsis*, reveals functional impact of cpCK2 phosphorylation. Plant J 62, 192–202. 20088902 10.1111/j.1365-313X.2010.04138.x PMC2988416

[54] Park ST , Kang C‐M & Husson RN (2008) Regulation of the SigH stress response regulon by an essential protein kinase in mycobacterium tuberculosis. Proc Natl Acad Sci USA 105, 13105–13110. 18728196 10.1073/pnas.0801143105 PMC2529121

[55] Hanahan D (1983) Studies on transformation of *Escherichia coli* with plasmids. J Mol Biol 166, 557–580. 6345791 10.1016/s0022-2836(83)80284-8

[56] da Silva ACR , Ferro JA , Reinach FC , Farah CS , Furlan LR , Quaggio RB , Monteiro‐Vitorello CB , Van Sluys MA , Almeida NF , Alves LMC *et al*. (2002) Comparison of the genomes of two *Xanthomonas* pathogens with differing host specificities. Nature 417, 459–463. 12024217 10.1038/417459a

[57] Bloomfield G , Tanaka Y , Skelton J , Ivens A & Kay RR (2008) Widespread duplications in the genomes of laboratory stocks of *Dictyostelium discoideum* . Genome Biol 9, R75. 18430225 10.1186/gb-2008-9-4-r75 PMC2643946

[58] Savitsky P , Bray J , Cooper CDO , Marsden BD , Mahajan P , Burgess‐Brown NA & Gileadi O (2010) High‐throughput production of human proteins for crystallization: the SGC experience. J Struct Biol 172, 3–13. 20541610 10.1016/j.jsb.2010.06.008 PMC2938586

[59] Aslanidis C & de Jong PJ (1990) Ligation‐independent cloning of PCR products (LIC‐PCR). Nucleic Acids Res 18, 6069–6074. 2235490 10.1093/nar/18.20.6069 PMC332407

[60] Stols L , Gu M , Dieckman L , Raffen R , Collart FR & Donnelly MI (2002) A new vector for high‐throughput, ligation‐independent cloning encoding a tobacco etch virus protease cleavage site. Protein Expr Purif 25, 8–15. 12071693 10.1006/prep.2001.1603

[61] Gileadi O , Burgess‐Brown NA , Colebrook SM , Berridge G , Savitsky P , Smee CEA , Loppnau P , Johansson C , Salah E & Pantic NH (2008) High throughput production of recombinant human proteins for crystallography. Methods Mol Biol 426, 221–246. 18542867 10.1007/978-1-60327-058-8_14

[62] Strain‐Damerell C , Mahajan P , Gileadi O & Burgess‐Brown NA (2014) Medium‐throughput production of recombinant human proteins: ligation‐independent cloning. Methods Mol Biol 1091, 55–72. 24203324 10.1007/978-1-62703-691-7_4

[63] Newman J (2004) Novel buffer systems for macromolecular crystallization. Acta Crystallogr D Biol Crystallogr 60, 610–612. 14993709 10.1107/S0907444903029640

[64] Kabsch W (2010) XDS. Acta Crystallogr D Biol Crystallogr 66, 125–132. 20124692 10.1107/S0907444909047337 PMC2815665

[65] Winn MD , Ballard CC , Cowtan KD , Dodson EJ , Emsley P , Evans PR , Keegan RM , Krissinel EB , Leslie AGW , McCoy A *et al*. (2011) Overview of the CCP4 suite and current developments. Acta Crystallogr D Biol Crystallogr 67, 235–242. 21460441 10.1107/S0907444910045749 PMC3069738

[66] McCoy AJ , Grosse‐Kunstleve RW , Adams PD , Winn MD , Storoni LC & Read RJ (2007) Phaser crystallographic software. J Appl Crystallogr 40, 658–674. 19461840 10.1107/S0021889807021206 PMC2483472

[67] Cabarca S , Frazão de Souza M , Albert Oliveira A , Vignoli Muniz GS , Lamy MT , Vinicius Dos Reis C , Takarada J , Effer B , Souza LS , de la Iriarte Torre L *et al*. (2021) Structure of the PknF and conformational changes induced in forkhead‐associated regulatory domains. Curr Res Struct Biol 3, 165–178. 34382010 10.1016/j.crstbi.2021.07.001 PMC8339232

[68] Emsley P , Lohkamp B , Scott WG & Cowtan K (2010) Features and development of coot. Acta Crystallogr D Biol Crystallogr 66, 486–501. 20383002 10.1107/S0907444910007493 PMC2852313

[69] Murshudov GN , Vagin AA & Dodson EJ (1997) Refinement of macromolecular structures by the maximum‐likelihood method. Acta Crystallogr D Biol Crystallogr 53, 240–255. 15299926 10.1107/S0907444996012255

[70] Chen VB , Arendall WB 3rd , Headd JJ , Keedy DA , Immormino RM , Kapral GJ , Murray LW , Richardson JS & Richardson DC (2010) MolProbity: all‐atom structure validation for macromolecular crystallography. Acta Crystallogr D Biol Crystallogr 66, 12–21. 20057044 10.1107/S0907444909042073 PMC2803126

[71] Righetto GL , Sriranganadane D , Halabelian L , Chiodi CG , Elkins JM , Massirer KB , Gileadi O , Menossi M & Couñago RM (2019) The C‐terminal domains SnRK2 box and ABA box have a role in sugarcane SnRK2s auto‐activation and activity. Front Plant Sci 10, 1105. 31620147 10.3389/fpls.2019.01105 PMC6759772

[72] Drebes Dörr NC & Blokesch M (2020) Interbacterial competition and anti‐predatory behaviour of environmental *Vibrio cholerae* strains. Environ Microbiol 22, 4485–4504. 32885535 10.1111/1462-2920.15224 PMC7702109

[73] Sillo A , Matthias J , Konertz R , Bozzaro S & Eichinger L (2011) *Salmonella typhimurium* is pathogenic for *Dictyostelium* cells and subverts the starvation response. Cell Microbiol 13, 1793–1811. 21824247 10.1111/j.1462-5822.2011.01662.x

[74] Edgar RC (2004) MUSCLE: a multiple sequence alignment method with reduced time and space complexity. BMC Bioinformatics 5, 113. 15318951 10.1186/1471-2105-5-113 PMC517706

[75] Wright ES (2015) DECIPHER: harnessing local sequence context to improve protein multiple sequence alignment. BMC Bioinformatics 16, 322. 26445311 10.1186/s12859-015-0749-z PMC4595117

[76] Zhou L , Feng T , Xu S , Gao F , Lam TT , Wang Q , Wu T , Huang H , Zhan L , Li L *et al*. (2022) ggmsa: a visual exploration tool for multiple sequence alignment and associated data. Brief Bioinform 23, bbac222. 35671504 10.1093/bib/bbac222

