# Supplementary material for: A Hanks‐type bacterial kinase, PknS, directly phosphorylates the alternative sigma factor EcfK to promote resistance to protist predation
Source: FEBS J. 2026 Jan 8;293(9):2772–93. doi: 10.1111/febs.70384 (PMC13147305; doi:10.1111/febs.70384)
Supplement: Supplementary file 1 — Fig. S1. Analysis of PknS1–364 purification and autophosphorylation. Fig. S2. Functionality of Flag‐tagged versions of PknS in X. citri, evaluated by ability to complement the phenotype of the ΔpknS strain and restore the resistance to amoeba predation. Fig. S3. Crystal packing for PknS1–364M164A:CHIR‐124 co‐crystals and predicted structures of residues 1–81 in PknS. Fig S4. His6‐EcfK purification. Fig. S5. Mass spectra (A) and deconvoluted mass spectra (B) obtained by LC/MS analysis of His6‐EcfK incubated with ATP and PknS1‐364. Fig. S6. Mass spectra (A) and deconvoluted mass spectra (B) of His6‐EcfK with ATP, as determined by LC/MS analysis. Fig. S7. Mass spectra of phosphorylated peptides of His6‐EcfK identified by LC‐MS/MS. Fig. S8. Results of individual plaque assay experiments, which were combined and displayed as relative values in Fig. 5D. Fig. S9. Detailed views of potential contacts and charge complementarity between X. citri RpoC and the selected ECF sigma factors. Fig. S10. Quality assessment of AlphaFold3‐predicted structures of PknS shown in Fig. S3. Fig. S11. Quality assessment of the AlphaFold3‐predicted structure of Holo‐RNA Polymerase complexes for X. citri pv. citri 306 bound to sigma factor EcfK (XAC4128). Fig. S12. Quality assessment of the AlphaFold3‐predicted structure of Holo‐RNA Polymerase complexes for X. citri pv. citri 306 bound to sigma factor RfaY (XAC2814). Fig. S13. Quality assessment of the AlphaFold3‐predicted structure of Holo‐RNA Polymerase complexes for X. citri pv. citri 306 bound to sigma factor PrtI (XAC3989). Fig. S14. Quality assessment of the AlphaFold3‐predicted structure of Holo‐RNA Polymerase complexes for X. citri pv. citri 306 bound to sigma factor RpoE (XAC1380). Fig. S15. Quality assessment of the AlphaFold3‐predicted structure of Holo‐RNA Polymerase complexes for X. citri pv. citri 306 bound to sigma factor XAC0922. Fig. S16. Quality assessment of the AlphaFold3‐predicted structure of Holo‐RNA Polymerase complexes f [file FEBS-293-2772-s001.zip › febs70384-sup-0001-Supinfo.pdf]

Supplementary material

Title: A Hanks-Type Bacterial Kinase Directly Phosphorylates the Alternative Sigma Factor EcfK to Promote Resistance to Protist Predation

Authors: Lídia dos Passos Lima, Dev Sriranganadane, Daiane Laise da Silva, Natália Carolina Drebes Dörr, Enzo Breviglieri Sichi Mello, Caio Vinicius dos Reis, Rogério Ferreira Lourenço, José Felipe Teixeira da Silva Santos, Anita Salmazo, Brenno Wendler Miranda, Katlin B. Massirer, Rafael M. Couñago, Cristina E. Alvarez-Martinez

FIGURE S1

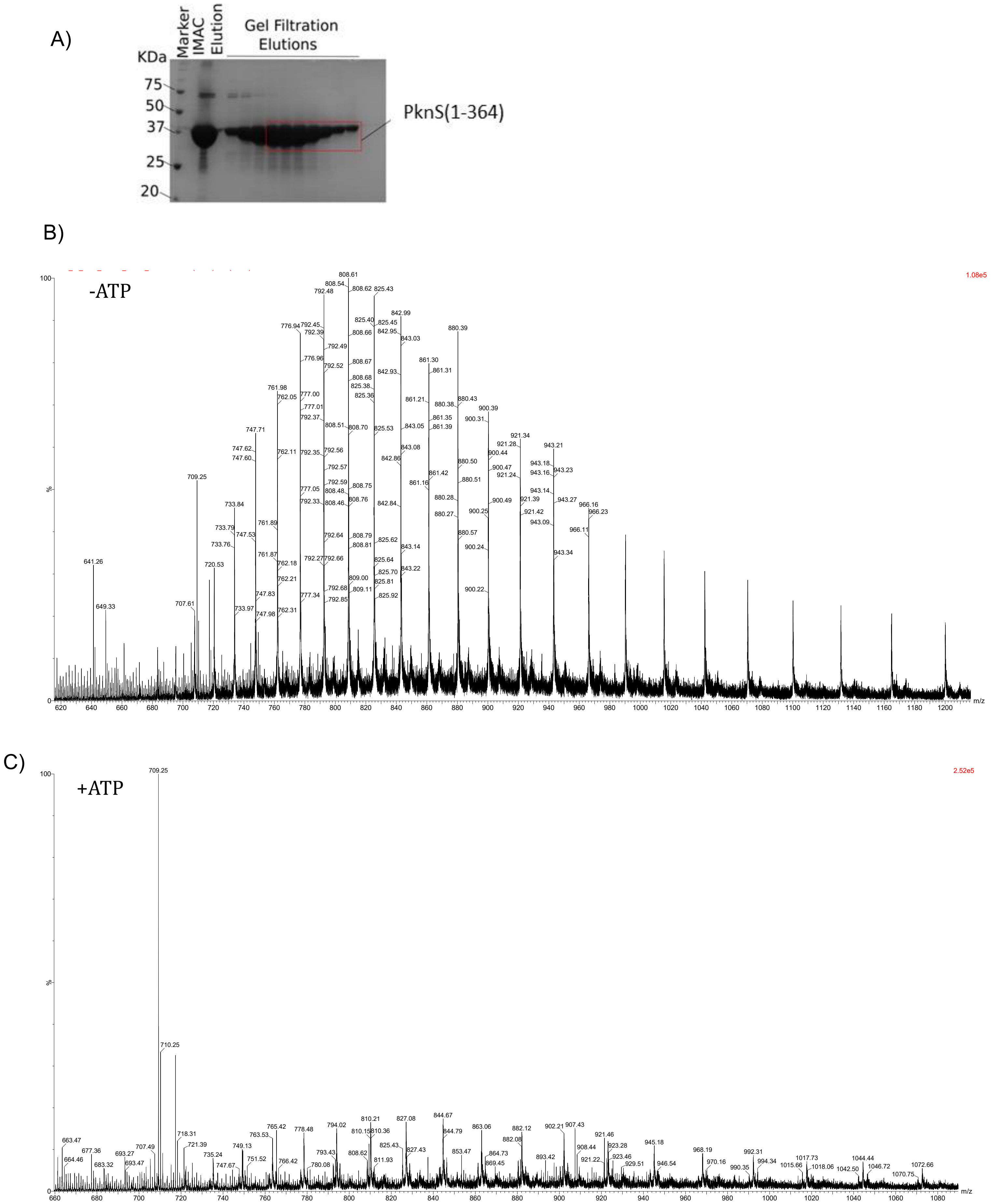
A)
Marker
IMAC Elution
Gel Filtration Elutions
KDa
75
50
37
25
20
PknS(1-364)
B)
-ATP
1.08e5
%
m/z
C)
+ATP
2.52e5
709.25
710.25
%
m/z

Figure S1: Analysis of $PknS_{1-364}$ purification and autophosphorylation. A) SDS-PAGE analysis of the purified $PknS_{1-364}$ after IMAC and gel filtration purification. Fractions containing $PknS_{1-364}$ (red box) were combined and then analyzed by LC-MS, showing the expected mass of 39576 Da. B) Mass spectra of $PknS_{1-364}$ by LC/MS analysis. C) Mass spectra of $PknS_{1-364}$ autophosphorylation by LC/MS analysis.

FIGURE S2

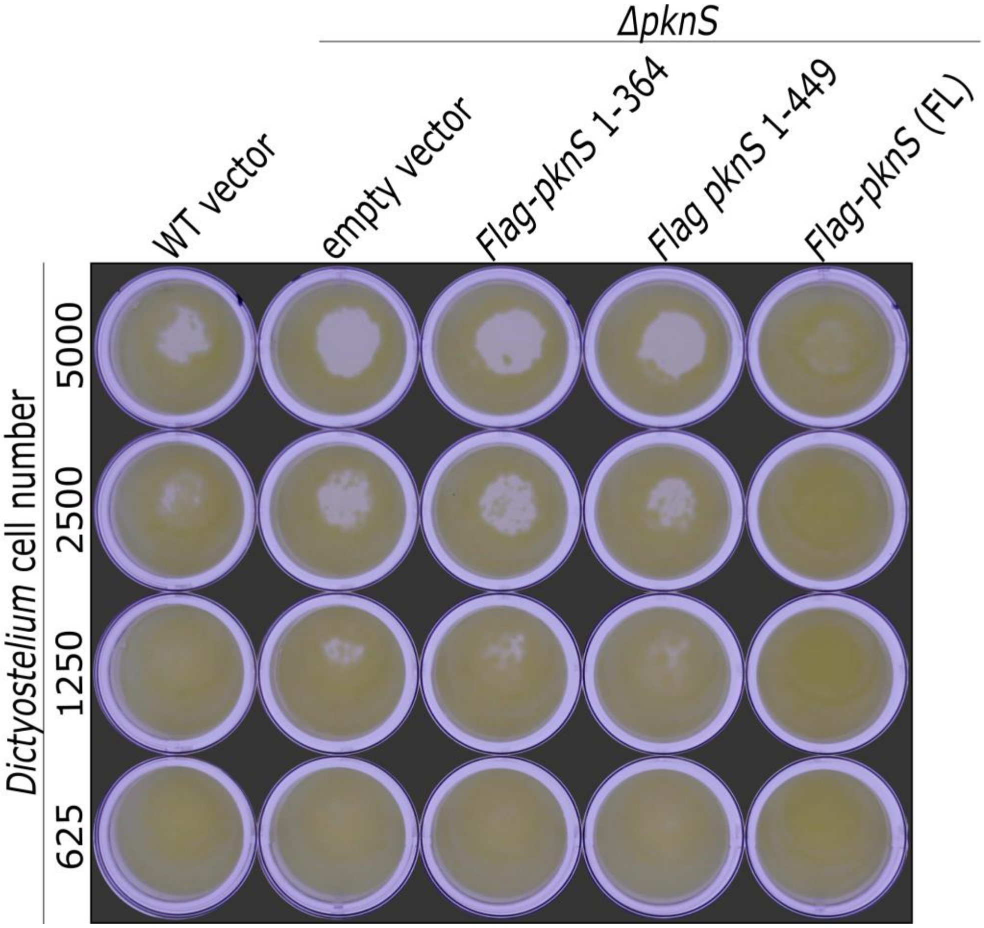

Figure S2. Functionality of Flag-tagged versions of PknS in *X. citri*, evaluated by ability to complement the phenotype of the Δ*pknS* strain and restore the resistance to amoeba predation. Susceptibility to amoeba predation was evaluated by phagocytic plaque formation assay [22], using the WT and Δ*pknS* carrying the empty vector, and Δ*pknS* carrying the pBRA plasmid with Flag-PknS$_{(1\text{-}364)}$, Flag-PknS$_{(1\text{-}449)}$ or Flag-PknS$_{(FL)}$, as indicated. Bacterial cultures were normalized to OD_{600nm}= 3.0 and spread onto solid media in 24-well plates, forming a bacterial lawn. Increasing amounts of *D. discoideum* cells (indicated in the figure) were spotted in a 5 µl volume in the center of the bacterial lawns and co-cultures were incubated for 5 days at 22ºC. Results are representative of 3 independent experiments.

Table S2 – Data collection and Refinement Statistics

PDB ID	9EED
Ligand	CHIR-124
Protein	*X. citri* PknS (XAC4127)
Diffraction source	DLS beamline I24
Wavelength (Å)	0.96858
Temperature (K)	100
Detector	PILATUS3 6M
Space group	P41
a=b, c (Å)	76.7, 58.9
α=β=γ (°)	90
Resolution range (Å)	29.65 -2.10 (2.16–2.10)
Total No. of reflections	207,179 (17,457)
No. of unique reflections	201,39 (1,657)
Completeness (%)	100.0 (100.0)
Redundancy	10.3 (10.5)
⟨ I/σ(I)⟩	14.1 (1.5)
Rmerge	0.076 (1.457)
Overall B factor from Wilson plot (Å2)	45.9
Resolution range (Å)	25.00–2.10 (2.10–2.15)
Completeness (%)	99.9 (100.0)
Final Rcryst	0.196 (0.305)
Final Rfree	0.235 (0.328)
No. of non-H atoms (Average B factors - Å2)	2114
Protein	2012 (58.6)
Ligand (CHIR-124)	30 (61.7)
Solvent	72 (58.8)
R.m.s. deviations	
Bonds (Å)	0.007
Angles (°)	1.592
Ramachandran plot	
Most favoured (%)	96
Outliers (%)	0.4
Crystallization conditions	0.2 M Lithium Sulfate; 0.1M SBG pH 6.5; 25% (w/v) PEG 3350

FIGURE S3

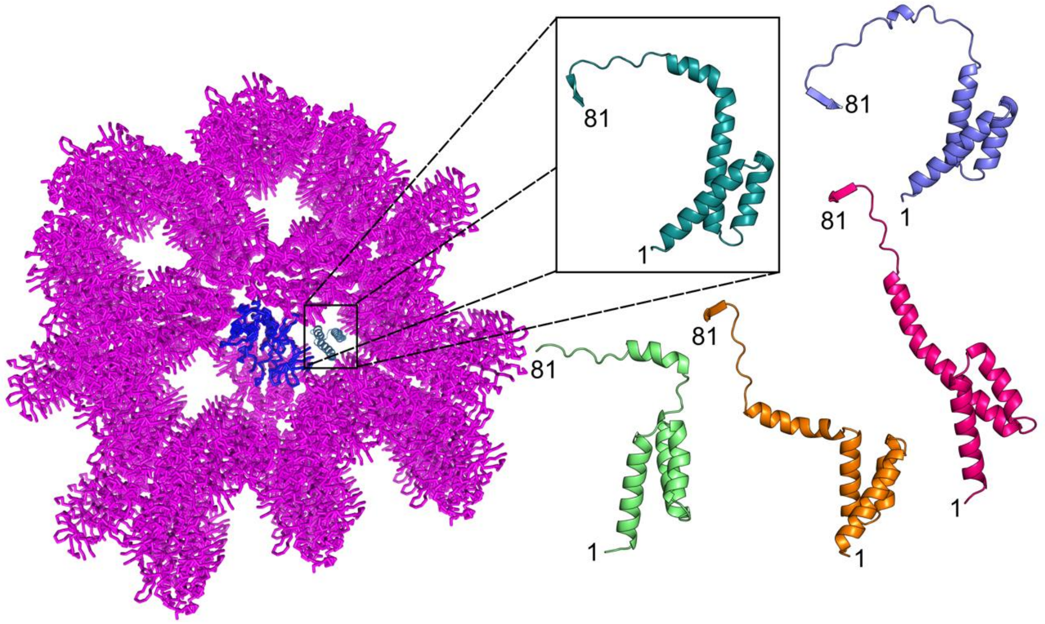

Figure S3: Crystal packing for $PknS_{1-364}M_{164}A$: CHIR-124 co-crystals and predicted structures of residues 1-81 in PknS. Blue ribbon depicts $PknS_{1-364}M_{164}A$ found in the crystal's asymmetric unit, while pink ribbons indicate its symmetry mates. The black box highlights the large, vacant space found within the crystal lattice. This space is large enough to manually place one copy of the AlphaFold2-predicted structures for residues 1-81 in PknS (right panel inset). Five of these predicted structures are shown in the right panel. Image produced using PyMOL Molecular Graphics System, Version 3.0.0. Quality assessment for AlphaFold2 structure prediction is shown in Figure S10.

FIGURE S4

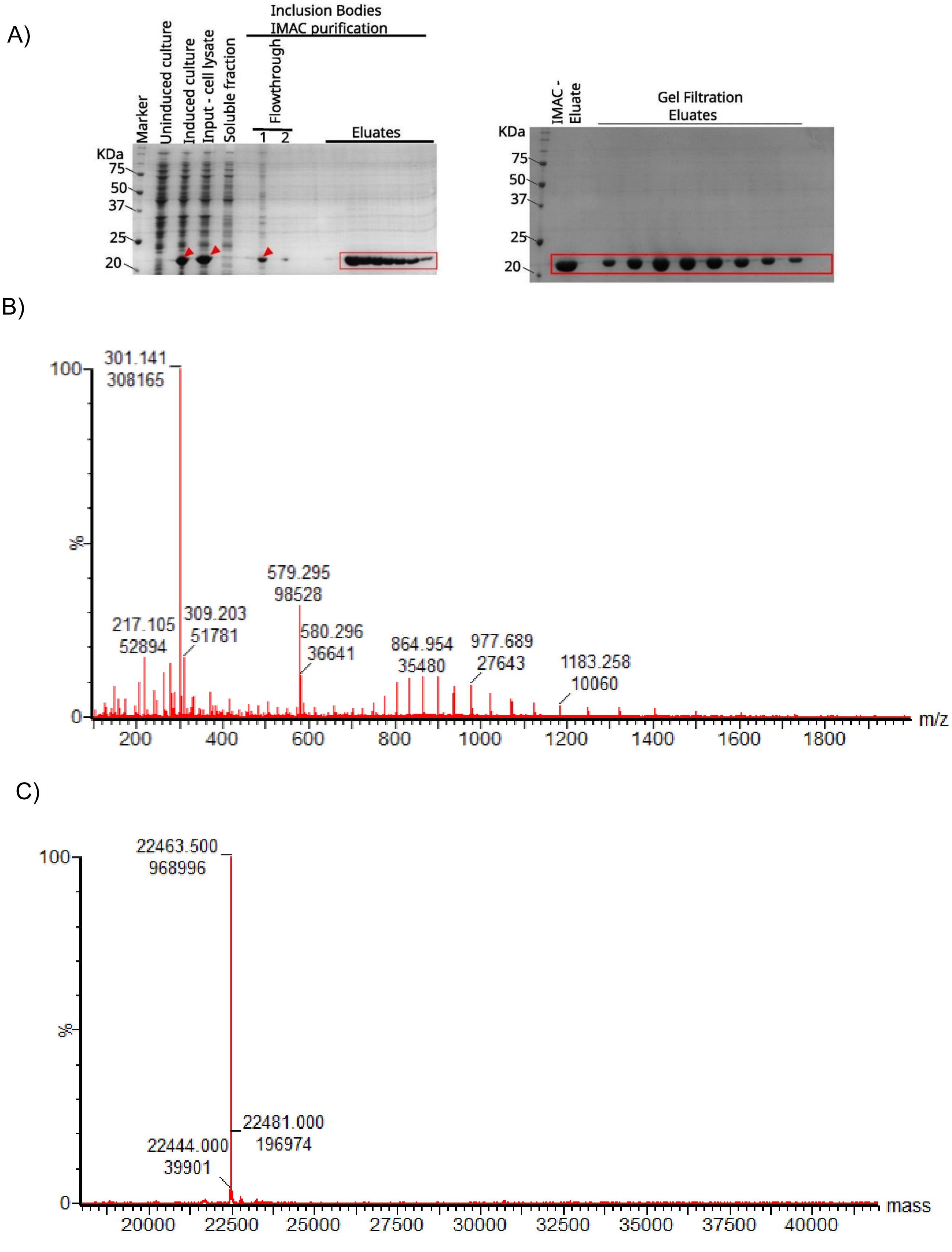

Figure S4: His$_6$-EcfK purification. SDS-PAGE analysis of His$_6$-EcfK purification (A). (B, C) mass spectra (B) and deconvoluted mass spectra (C) of His$_6$-EcfK as determined by LC/MS analysis.

FIGURE S5

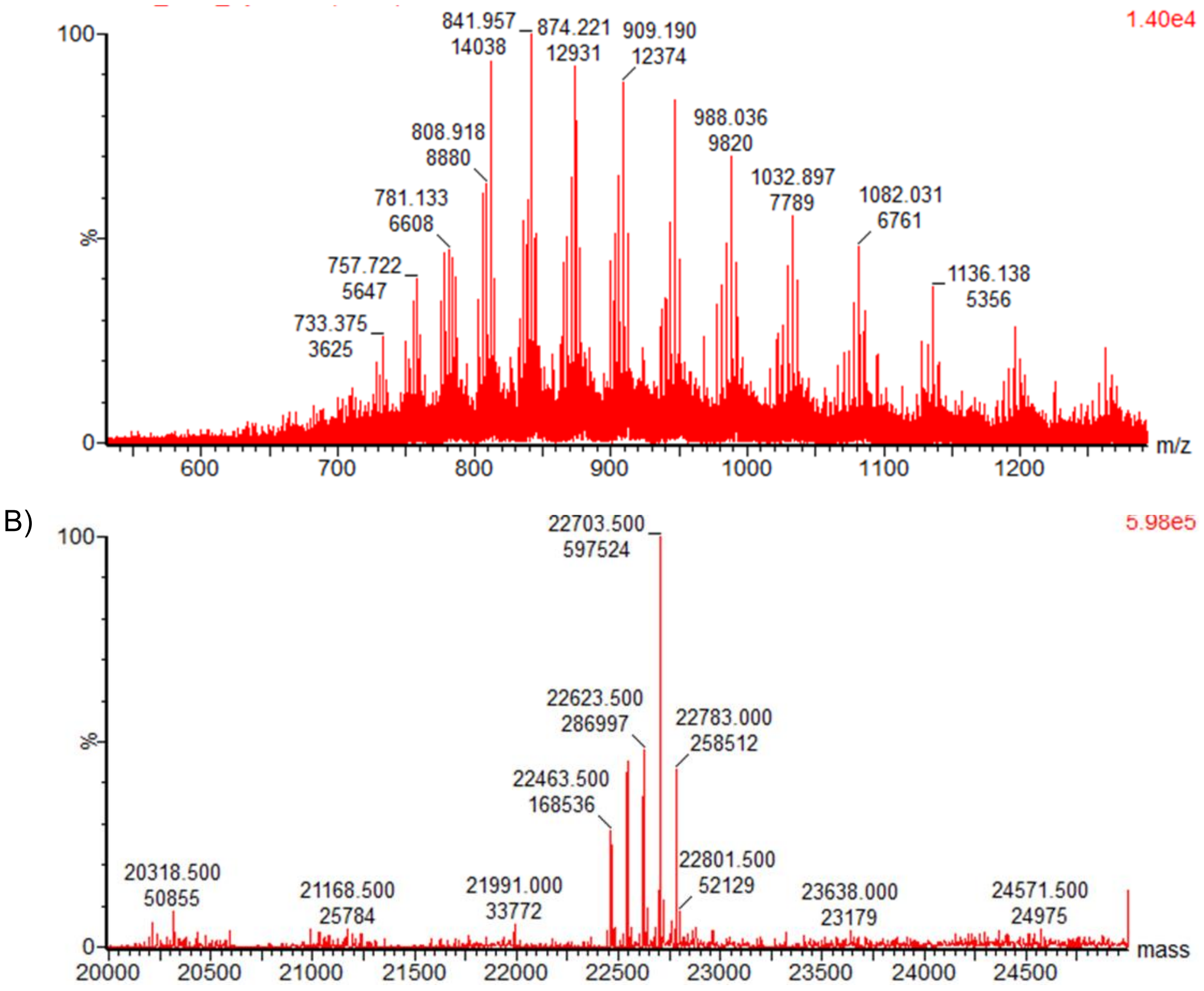

Figure S5: Mass spectra (A) and deconvoluted mass spectra (B) obtained by LC/MS analysis of His$_6$-EcfK incubated with ATP and PknS$_{1\text{-}364}$.

FIGURE S6

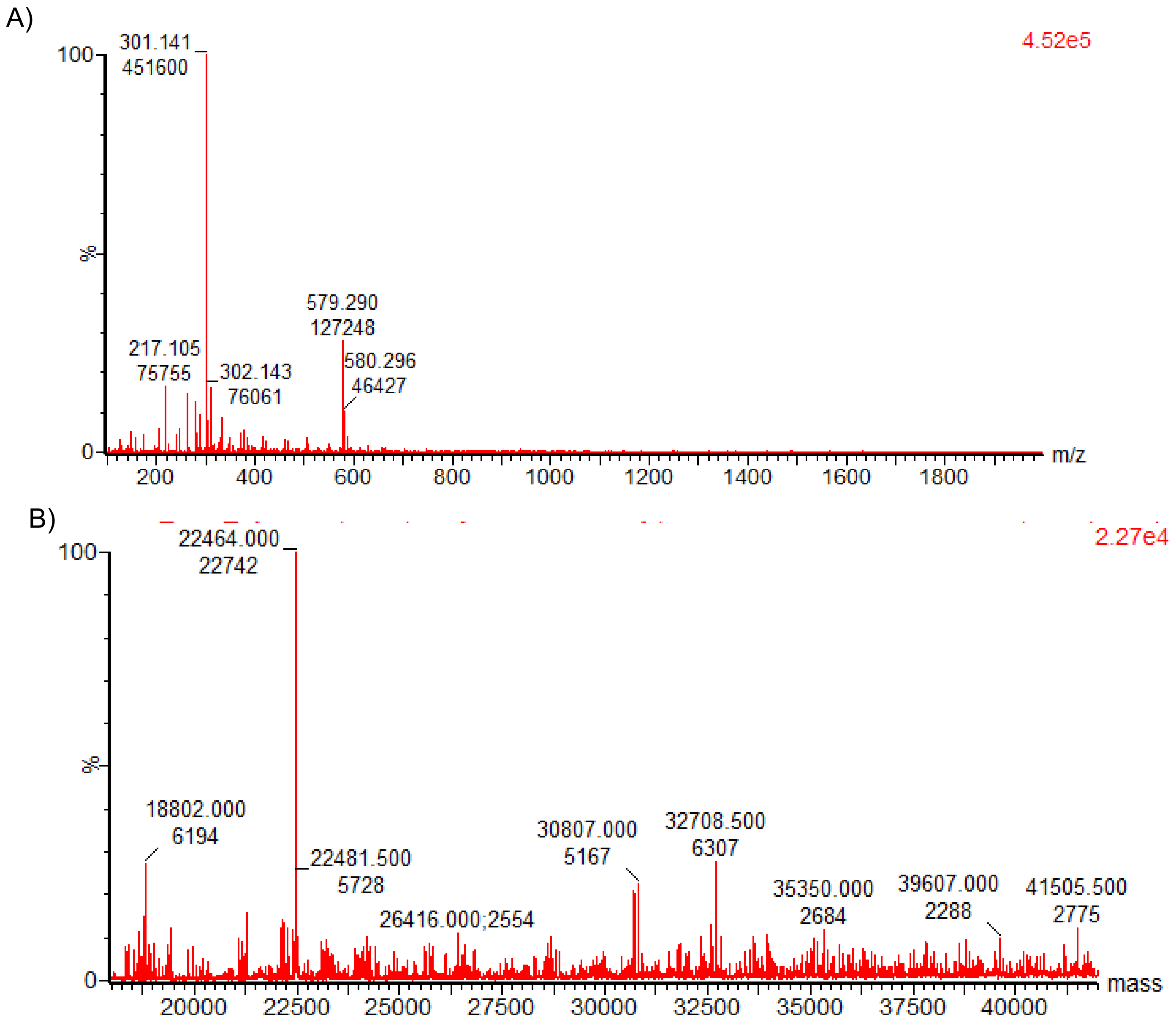

Figure S6: Mass spectra (A) and deconvoluted mass spectra (B) of His_6-EcfK with ATP, as determined by LC/MS analysis.

Table S3. Phosphorylated peptides of His$_6$-EcfK identified by LC-MS/MS.

protein.Entry	peptide.seq	peptide.modification	residue position	peptide.score
Q8PF57_XANAC	AGLQA**T**ELVHEAWMR	Phosphoryl+STY(6)	T51	9.1
		Oxidation+M(14);Phosphoryl+STY(6)		6.9
	LGQAV**T**L**T**ISLSDGAARPEALMLVADAFDK	Phosphoryl+STY(6);Phosphoryl+STY(8)	T104, T106	8.3
	LGQAV**T**LTISLSDGAARPEALMLVADAFDK	Phosphoryl+STY(6)	T104	7.2
	LGQAV**T**LTISL**S**DGAARPEALMLVADAFDK	Phosphoryl+STY(6);Phosphoryl+STY(12)	T104, S110	7.6
	LGQAVTL**T**ISL**S**DGAARPEALMLVADAFDK	Phosphoryl+STY(8);Phosphoryl+STY(12)	T106, S110	7.6
	LGQAVTLTI**S**LSDGAARPEALMLVADAFDK	Phosphoryl+STY(10)	S108	7.5
		Oxidation+M(22);Phosphoryl+STY(10)		7.6
	LGQAVTL**T**ISLSDGAARPEALMLVADAFDKLAQVDER	Phosphoryl+STY(8)	T106	8.8
	LGQAVTL**T**ISL**S**DGAARPEALMLVADAFDKLAQVDER	Phosphoryl+STY(8);Phosphoryl+STY(12)	T106, S110	8.4
	LGQAV**T**LTISLSDGAARPEALMLVADAFDKLAQVDER	Phosphoryl+STY(6)	T104	8.2
	LGQAV**T**LTI**S**LSDGAARPEALMLVADAFDKLAQVDER	Phosphoryl+STY(6);Phosphoryl+STY(10)	T104, S108	7.4
	RLGQAV**T**LTI**S**LSDGAARPEALMLVADAFDK	Phosphoryl+STY(7);Phosphoryl+STY(11)	T104, S108	8.8
	RLGQAV**T**LTISLSDGAARPEALMLVADAFDK	Phosphoryl+STY(7)	T104	8.5
	RLGQAVTLTI**S**LSDGAARPEALMLVADAFDK	Phosphoryl+STY(11)	S108	8.2
	RLGQAVTLTISL**S**DGAARPEALMLVADAFDK	Phosphoryl+STY(13)	S110	7.4
	QVYDVLRA**T**AMR	Phosphoryl+STY(9)	T35	8.7

Table S4. His$_6$-EcfK peptides identified by LC-MS/MS. The table presents peptides with highest scores corresponding to 93.85% coverage of the hit "Extracytoplasmic sigma factor" from *Xanthomonas axonopodis* (Protein entry: Q8PF57_XANAC).

Protein Entry	Protein Description	Peptide Sequence	Peptide Unique Products	Peptide Raw Score	Peptide Score
Q8PF57_XANAC (Score: 38422.4)	Extracytoplasmic sigma factor OS=Xanthomonas axonopodis pv. citri	AFALTELVGFSVAEAAEQLEVSVPTLER	50	181717.3	9.8
		AKAFALTELVGFSVAEAAEQLEVSVPTLER	54	109423.8	9.6
		AHFYSVAALQMRHLLVDLAR	33	41293.23	9.2
		SRAHFYSVAALQMR	24	36903.55	9.2
		AGLQATELVHEAWMR	23	32436.29	9.1
		AHFYSVAALQMR	19	28465.83	9.0
		VWLAAQL	12	18106.65	8.8
		RLGQAVTLTISLSDGAARPEALMLVADAFDK	32	17409.3	8.8
		LGQAVTLTISLSDGAARPEALMLVADAFDKLAQVDER	41	17281.24	8.8
		LEQGQQGFR	12	15861.38	8.8
		HLLVDLAR	15	15002.49	8.8
		QVYDVLRATAMR	17	12442.58	8.7
		AFALTELVGFSVAEAAEQLEVSVPTLERDLR	26	11353.21	8.6
		QVYDVLR	12	10384.16	8.6
		LAQVDER	8	7151.111	8.4
		LGQAVTLTISLSDGAARPEALMLVADAFDK	15	5171.982	8.3
		DEPGAGDALAR	11	1853.504	7.9
		HLLVDLARQQASAK	11	1538.853	7.8
		MSELPITELLQAWQRDEPGAGDALAR	1	281.4959	7.0

FIGURE S7

Peptide Sequence	Peptide Modification	Peptide Score
AGLQATELVHEAWMR	Phosphoryl+STY(6)	9.0956

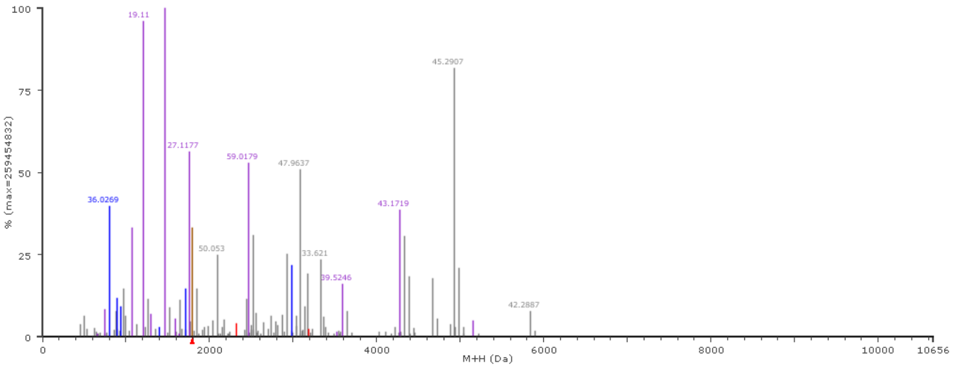

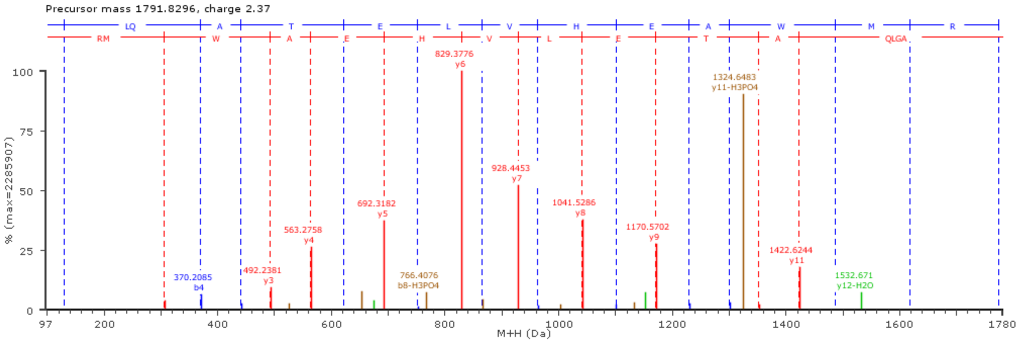

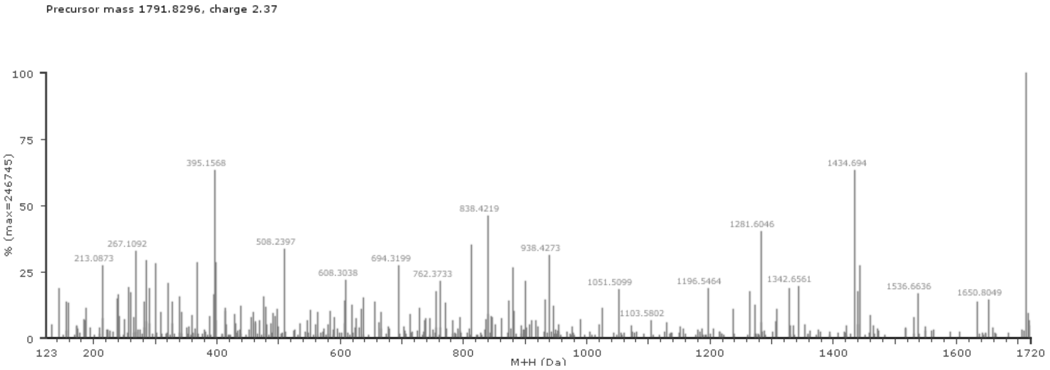

Peptide Sequence	Peptide Modification	Peptide Score
AGLQATELVHEAWMR	Oxidation+M(14);Phosphoryl+STY(6)	6.9422

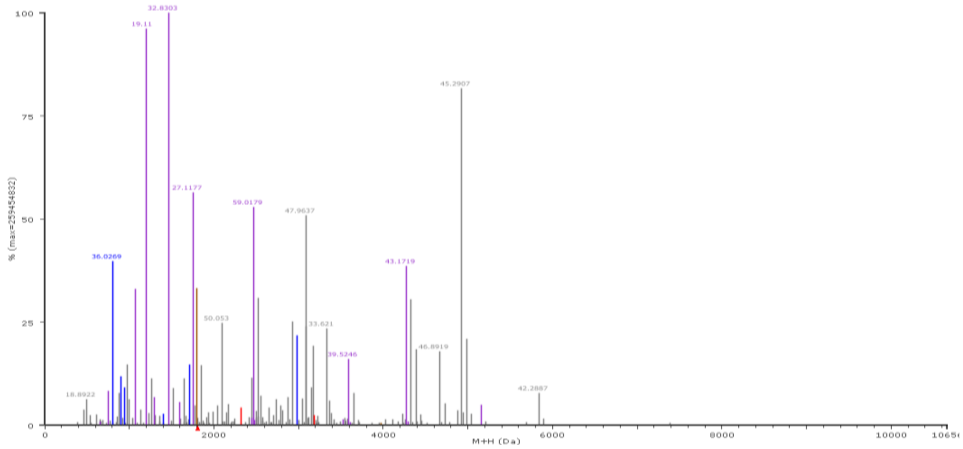

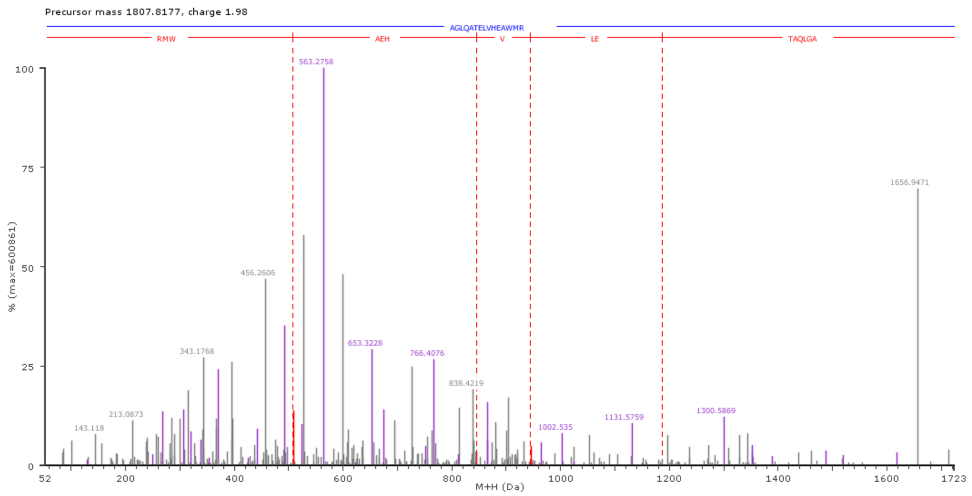

Peptide Sequence	Peptide Modification	Peptide Score
LGQAVTLTISLSDGAARPEALMLVADAFDK	Phosphoryl+STY(6);Phosphoryl+STY(8)	8.2982

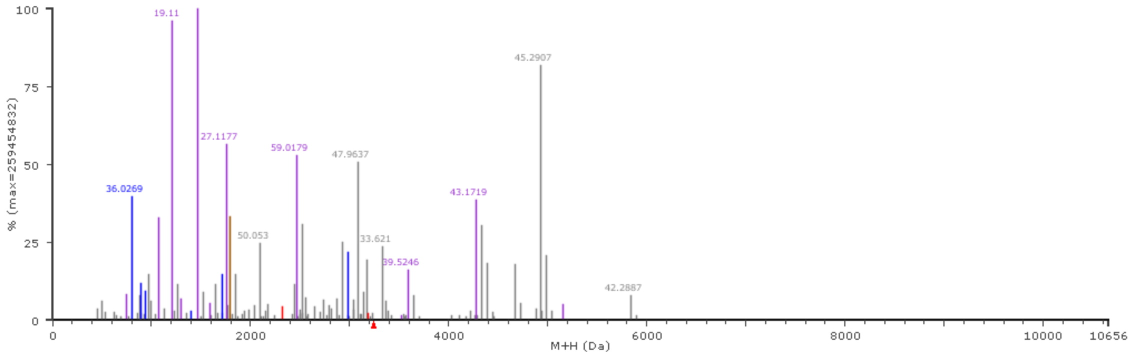

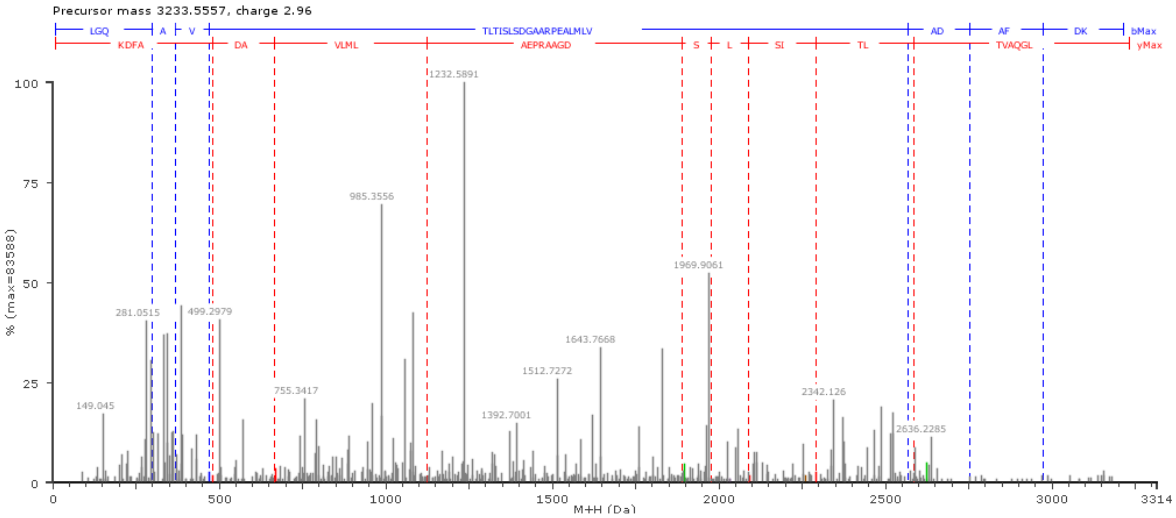

Peptide Sequence	Peptide Modification	Peptide Score
LGQAV**T**LTISLSDGAARPEALMLVADAFDK	Phosphoryl+STY(6)	7.1769

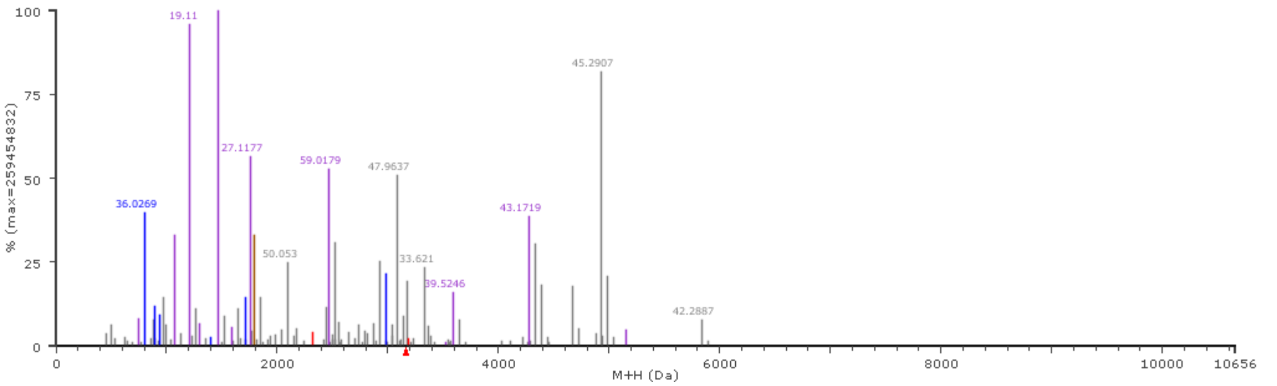

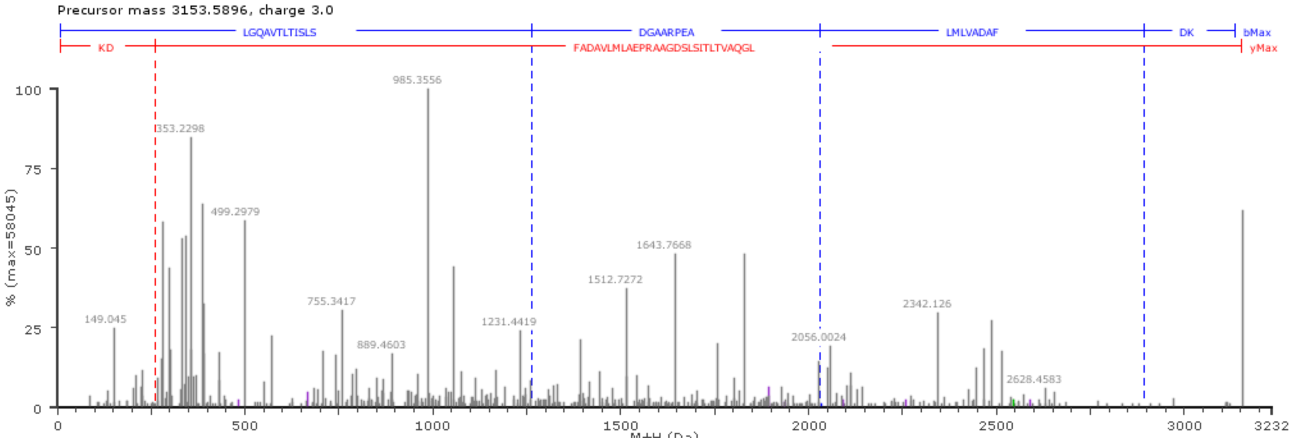

Peptide Sequence	Peptide Modification	Peptide Score
LGQAV**T**LTISL**S**DGAARPEALMLVADAFDK	Phosphoryl+STY(6);Phosphoryl+STY(12)	7.5762

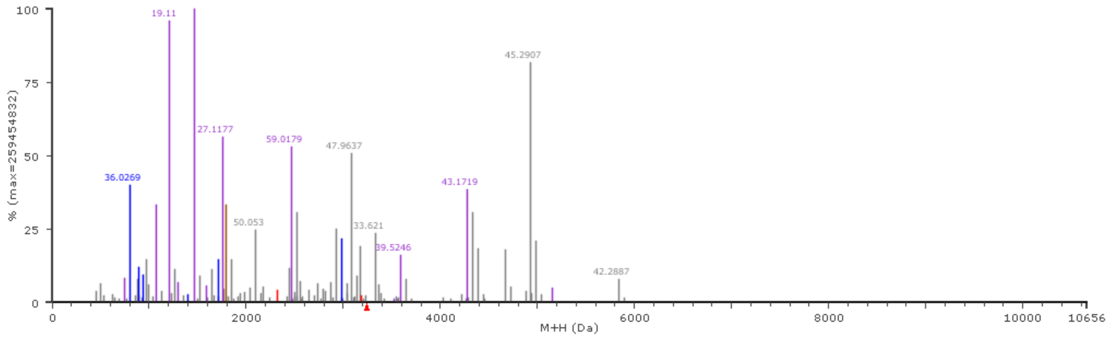

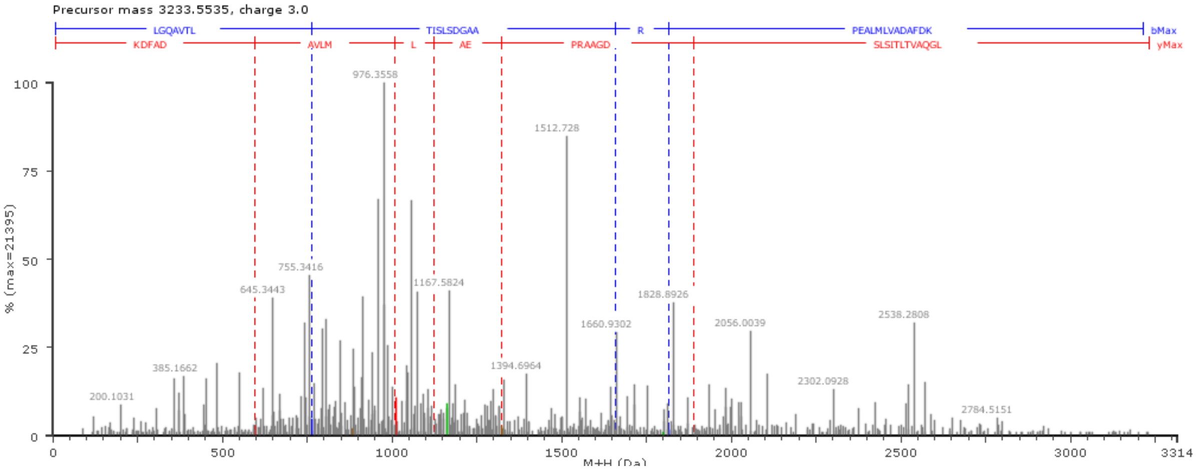

Peptide Sequence	Peptide Modification	Peptide Score
LGQAVTLTISLSDGAARPEALMLVADAFDK	Phosphoryl+STY(8);Phosphoryl+STY(12)	7.5914

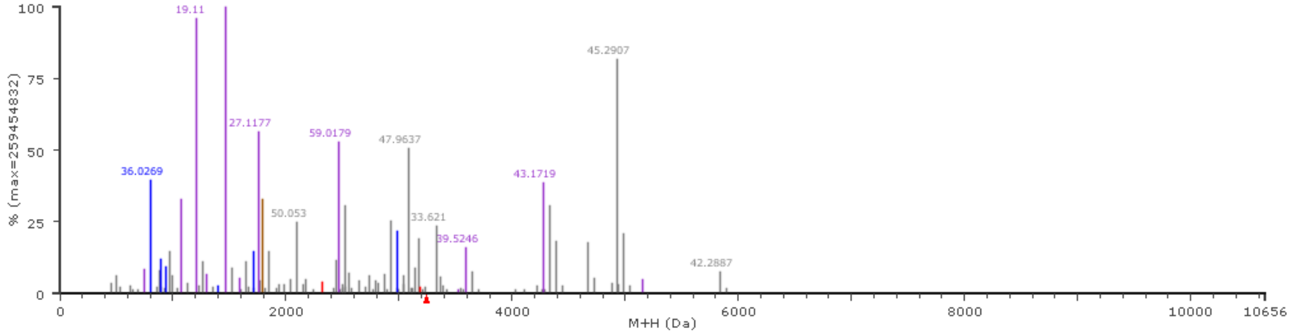

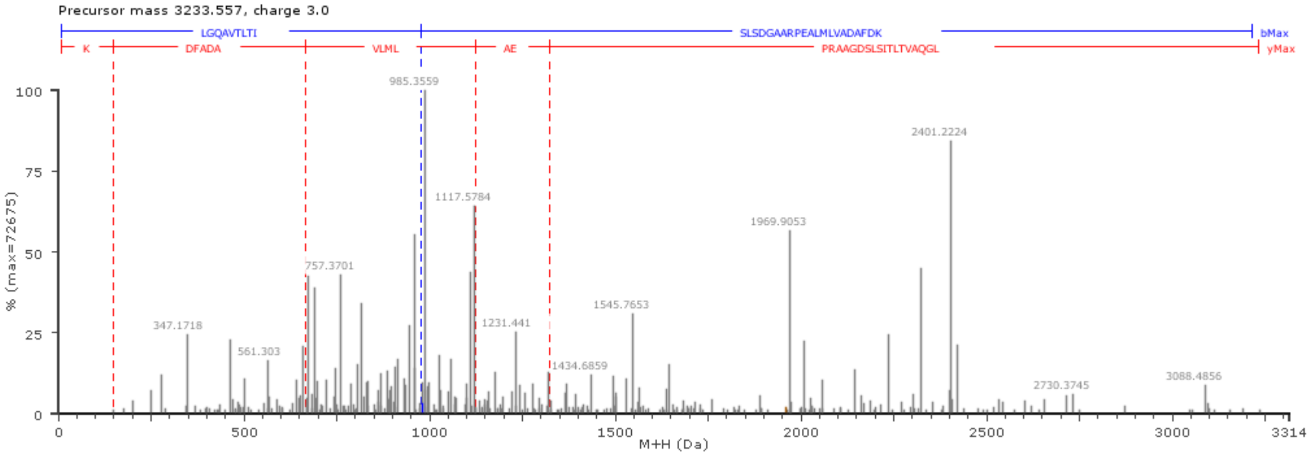

Peptide Sequence	Peptide Modification	Peptide Score
LGQAVTLTISLSDGAARPEALMLVADAFDK	Oxidation+M(22);Phosphoryl+STY(10)	7.551

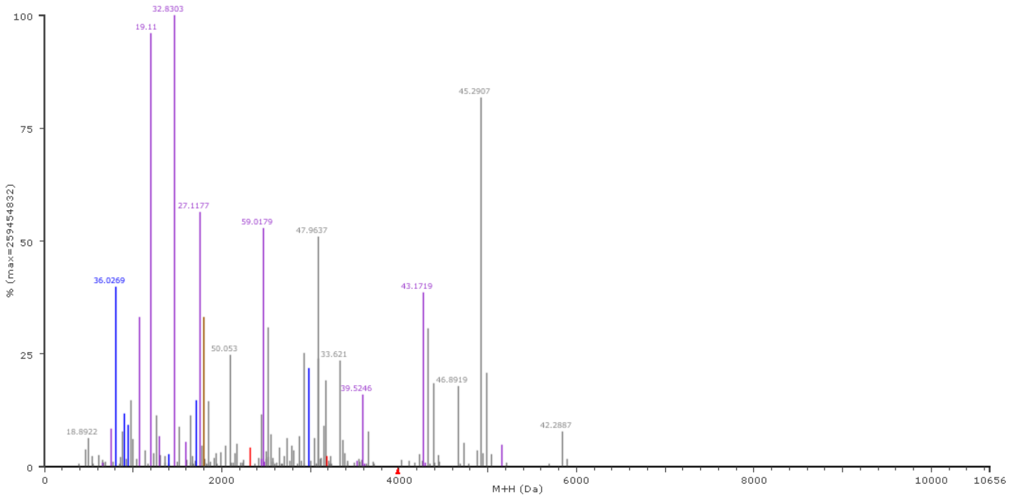

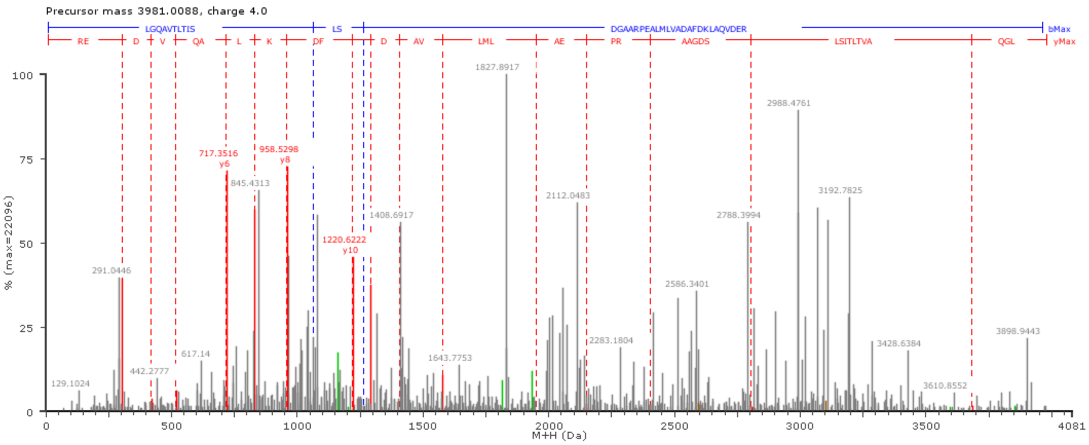

Peptide Sequence	Peptide Modification	Peptide Score
LGQAVTLTISLSDGAARPEALMLVADAFDKLAQVDER	Phosphoryl+STY(8)	8.8222

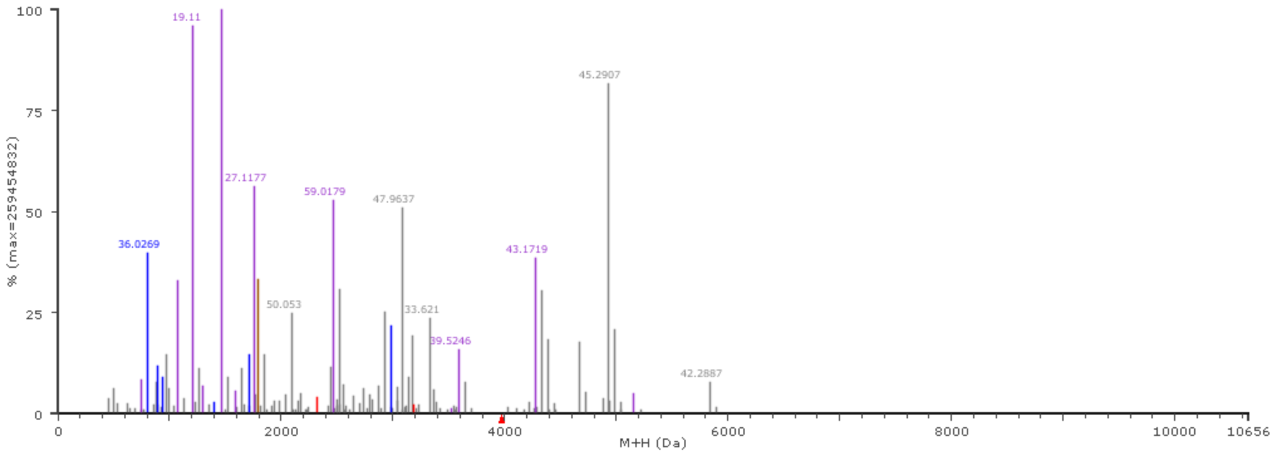

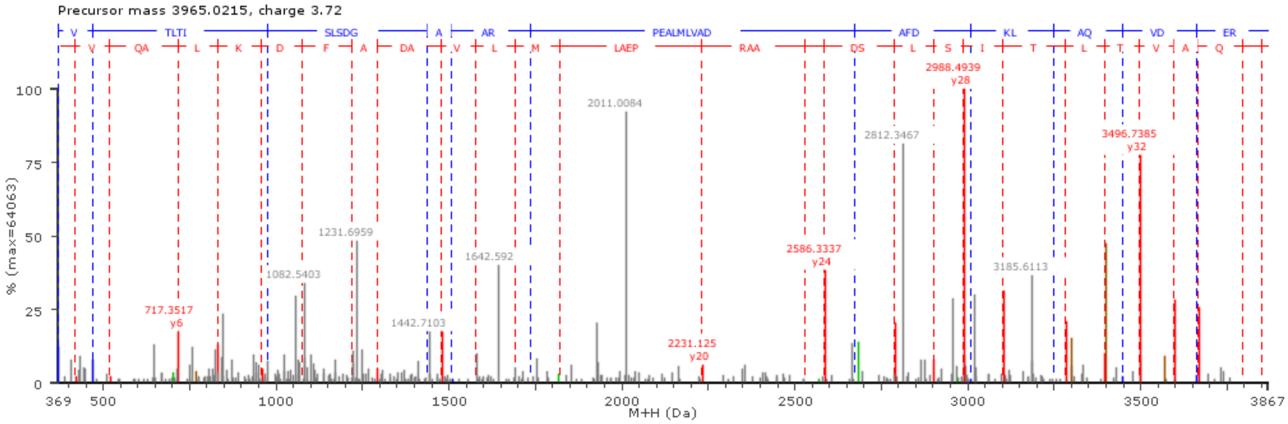

Peptide Sequence	Peptide Modification	Peptide Score
LGQAVTLTISLSDGAARPEALMLVADAFDKLAQVDER	Phosphoryl+STY(8);Phosphoryl+STY(12)	8.422

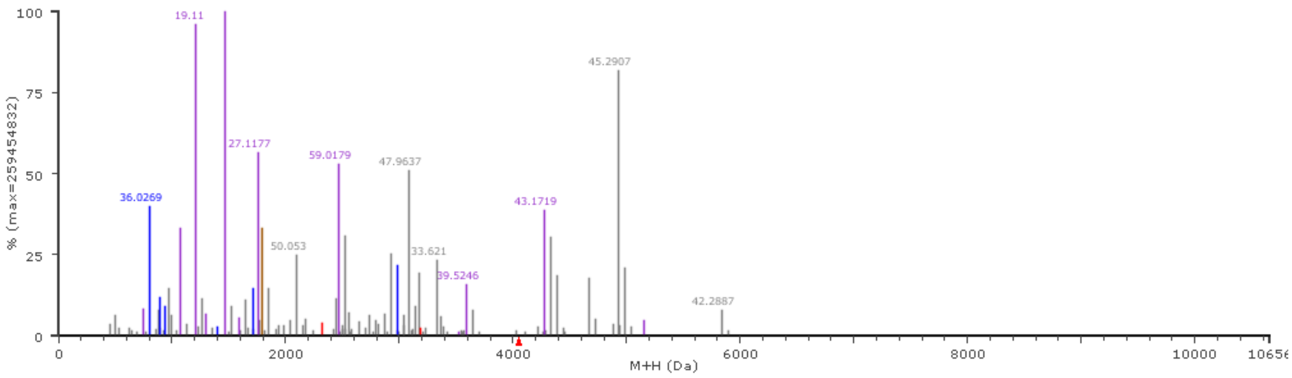

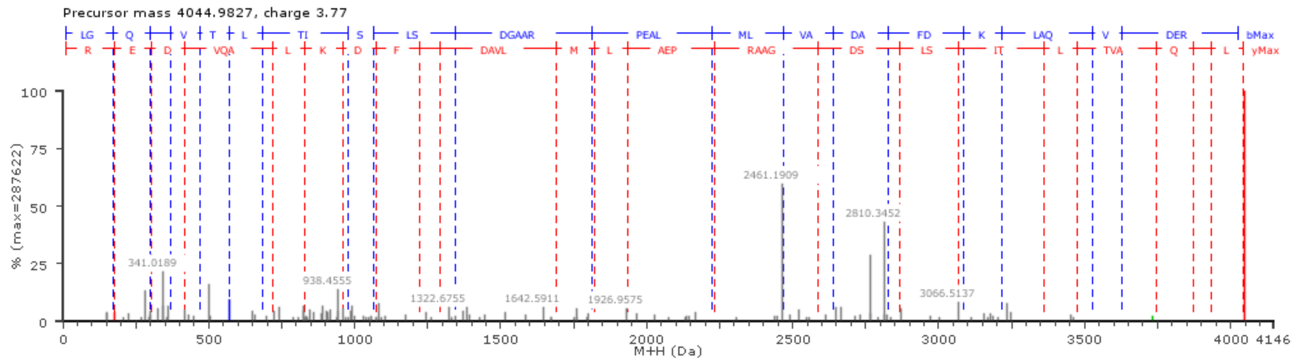

Peptide Sequence	Peptide Modification	Peptide Score
LGQAVTLTISLSDGAARPEALMLVADAFDKLAQVDER	Phosphoryl+STY(6)	8.1982

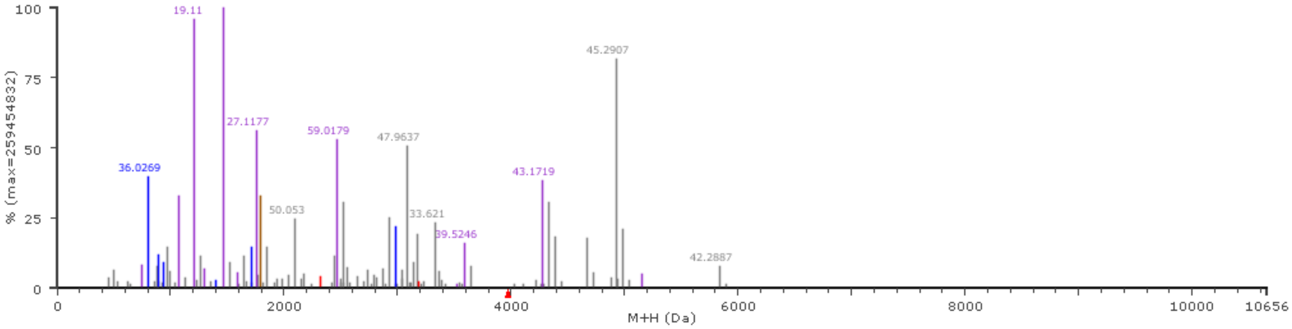

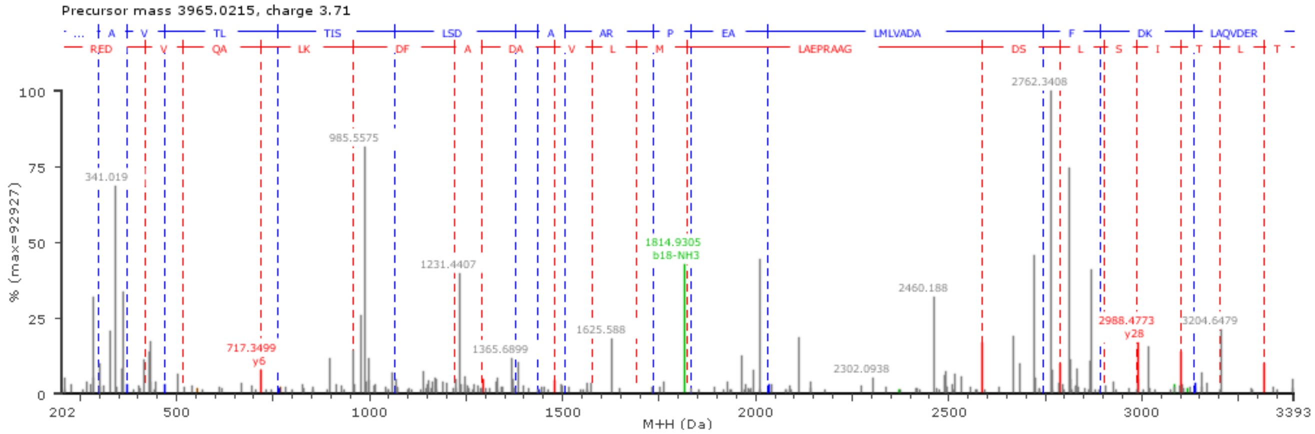

Peptide Sequence	Peptide Modification	Peptide Score
LGQAVTLTISLSDGAARPEALMLVADAFDKLAQVDER	Phosphoryl+STY(6);Phosphoryl+STY(10)	7.3967

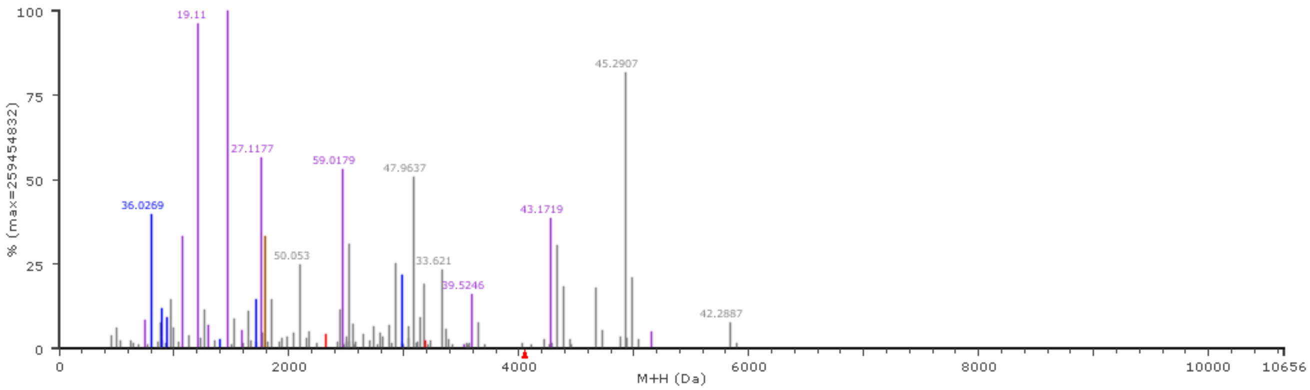

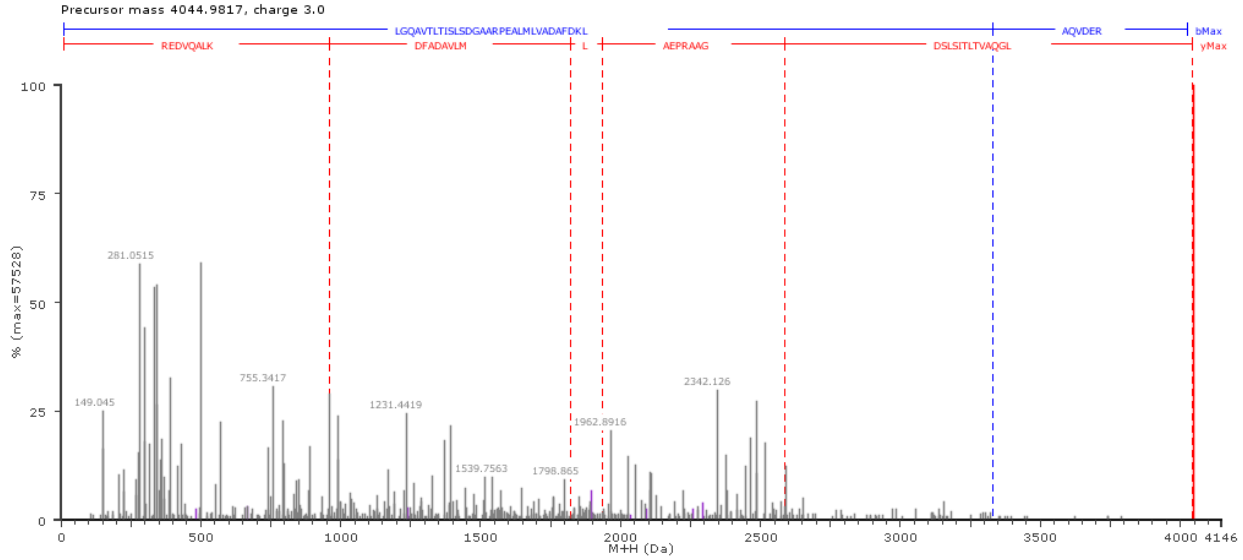

Peptide Sequence	Peptide Modification	Peptide Score
QVYDVLRATAMR	Phosphoryl+STY(9)	8.6795

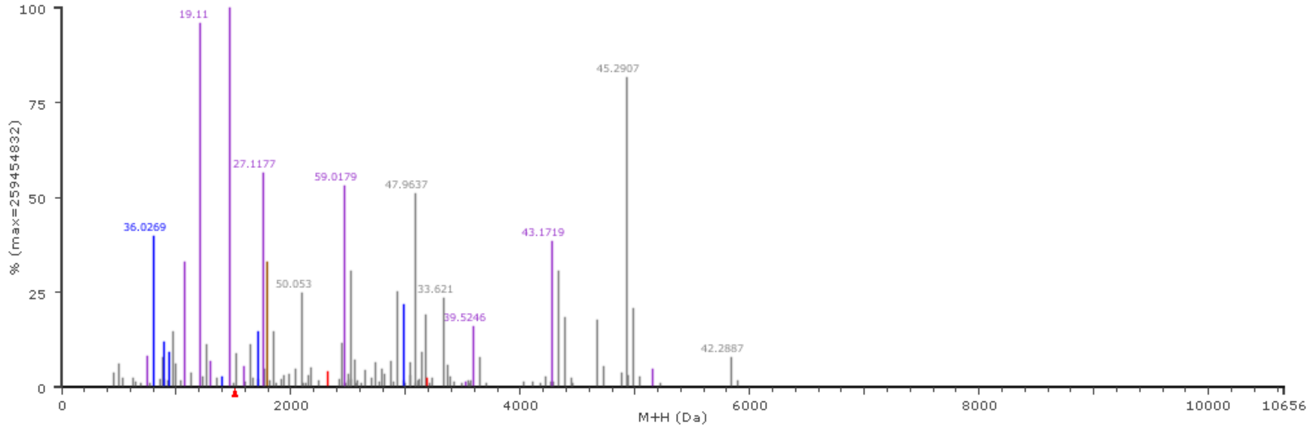

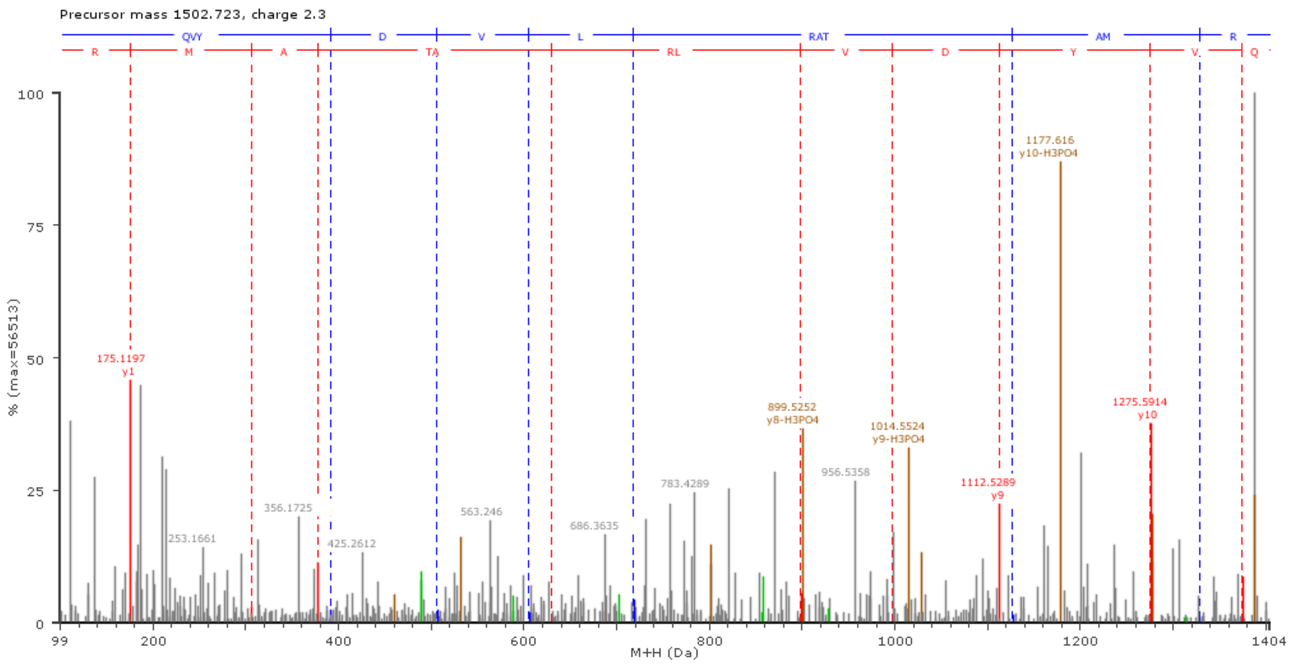

Peptide Sequence	Peptide Modification	Peptide Score
RLGQAV**T**LTI**S**LSDGAARPEALMLVADAFDK	Phosphoryl+STY(7);Phosphoryl+STY(11)	8.8254

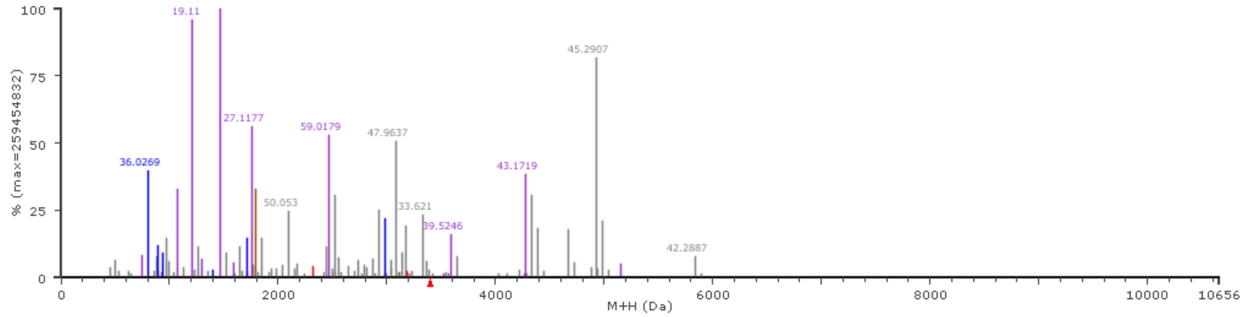

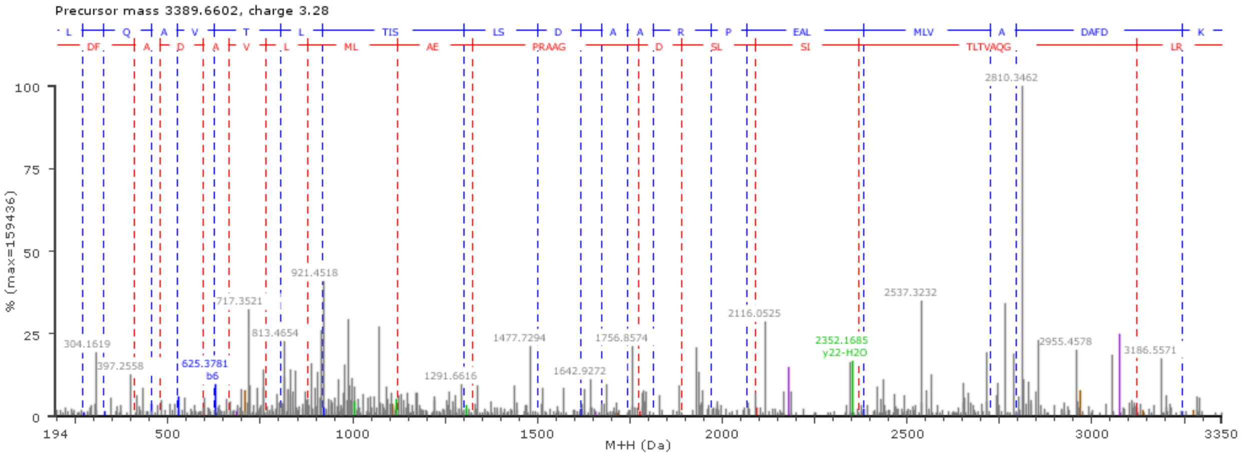

Peptide Sequence	Peptide Modification	Peptide Score
RLGQAV**T**LTISLSDGAARPEALMLVADAFDK	Phosphoryl+STY(7)	8.547

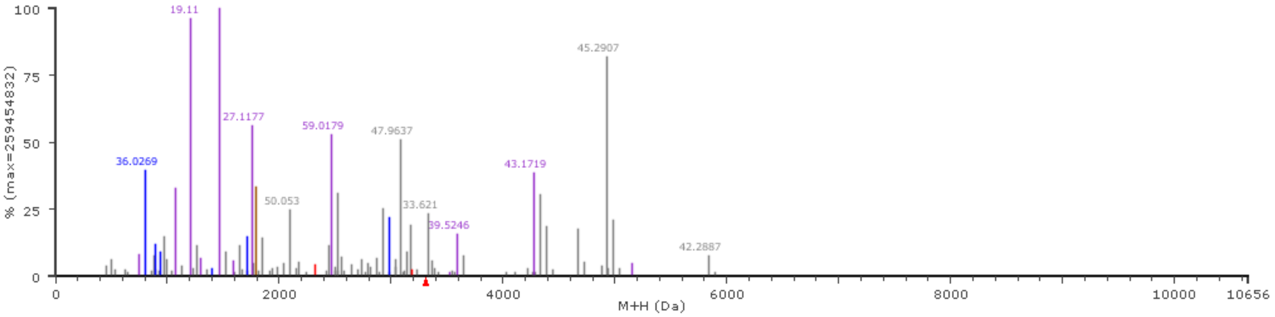

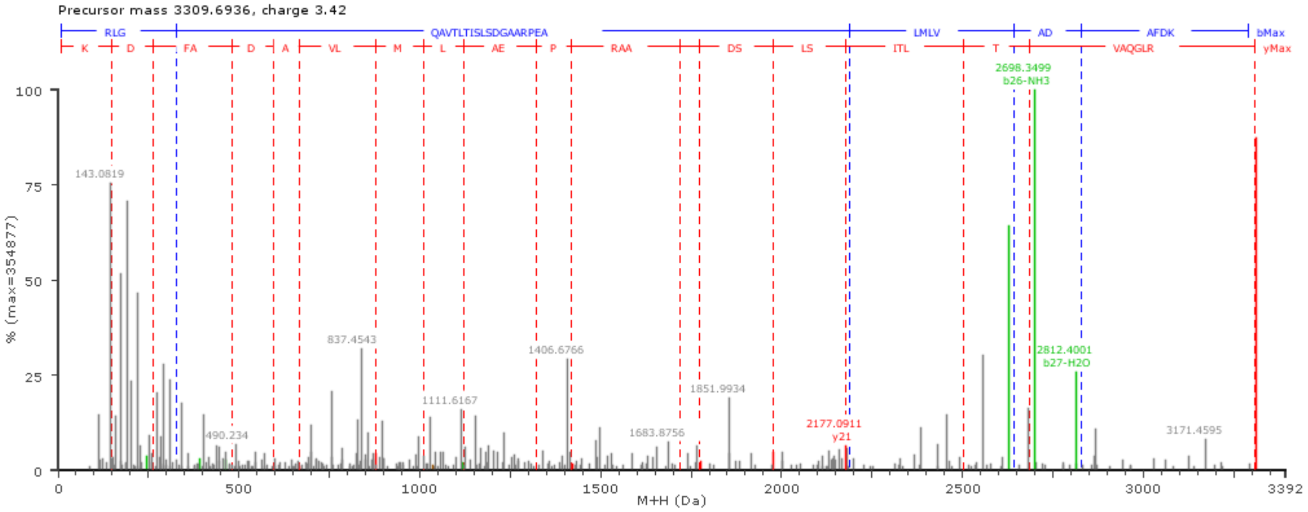

Peptide Sequence	Peptide Modification	Peptide Score
RLGQAVTLTI**S**LSDGAARPEALMLVADAFDK	Phosphoryl+STY(11)	8.2401

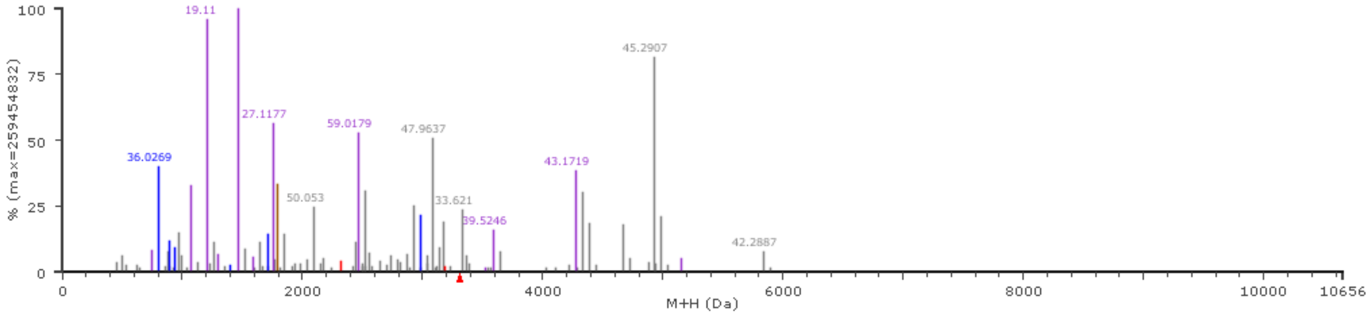

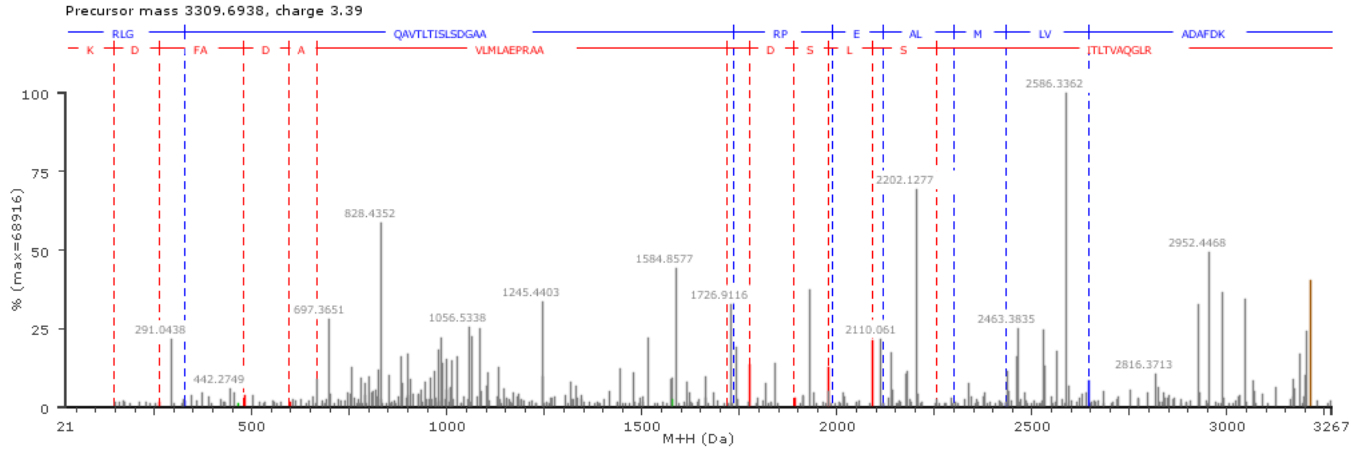

Peptide Sequence	Peptide Modification	Peptide Score
RLGQAVTLTISLSDGAARPEALMLVADAFDK	Phosphoryl+STY(13)	7.3982

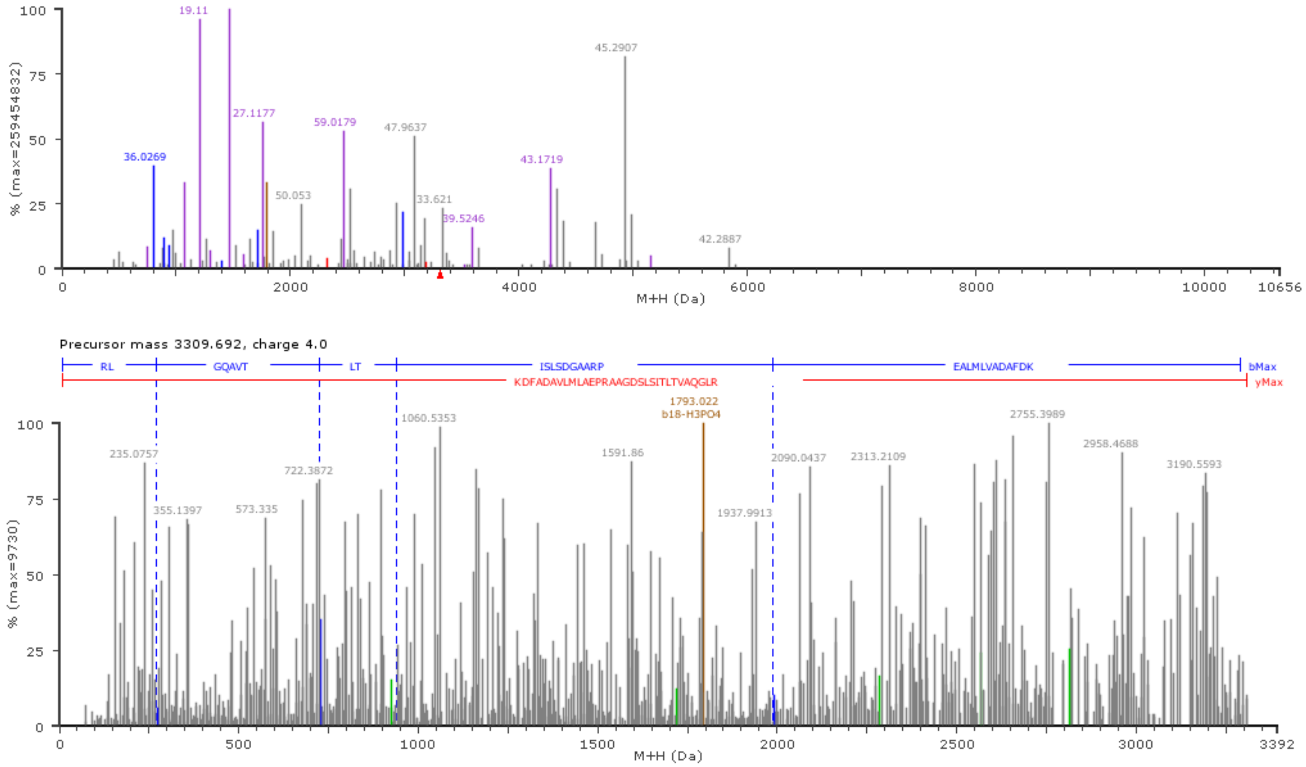

Figure S7: Mass spectra of phosphorylated peptides of His$_6$-EcfK identified by LC-MS/MS (corresponding to Table S3).

Table S6. Amino acid frequencies in all ECF43

	T104	L105	T106	I107	S108	L109	S110
**T**	8%	8%	43%	1%	6%	6%	3%
S	2%	1%	12%	1%	17%	4%	7%
E	3%	2%	11%	2%	13%	28%	15%
D	5%	1%	12%	1%	27%	23%	12%

Table S7. Amino acid frequencies Xanthomonadales' ECF43

	T104	L105	T106	I107	S108	L109	S110
**T**	16%	17%	47%	0%	6%	5%	1%
S	0%	1%	9%	0%	31%	2%	13%
E	1%	1%	14%	1%	8%	30%	8%
D	2%	2%	18%	0%	18%	15%	14%

Figure S8

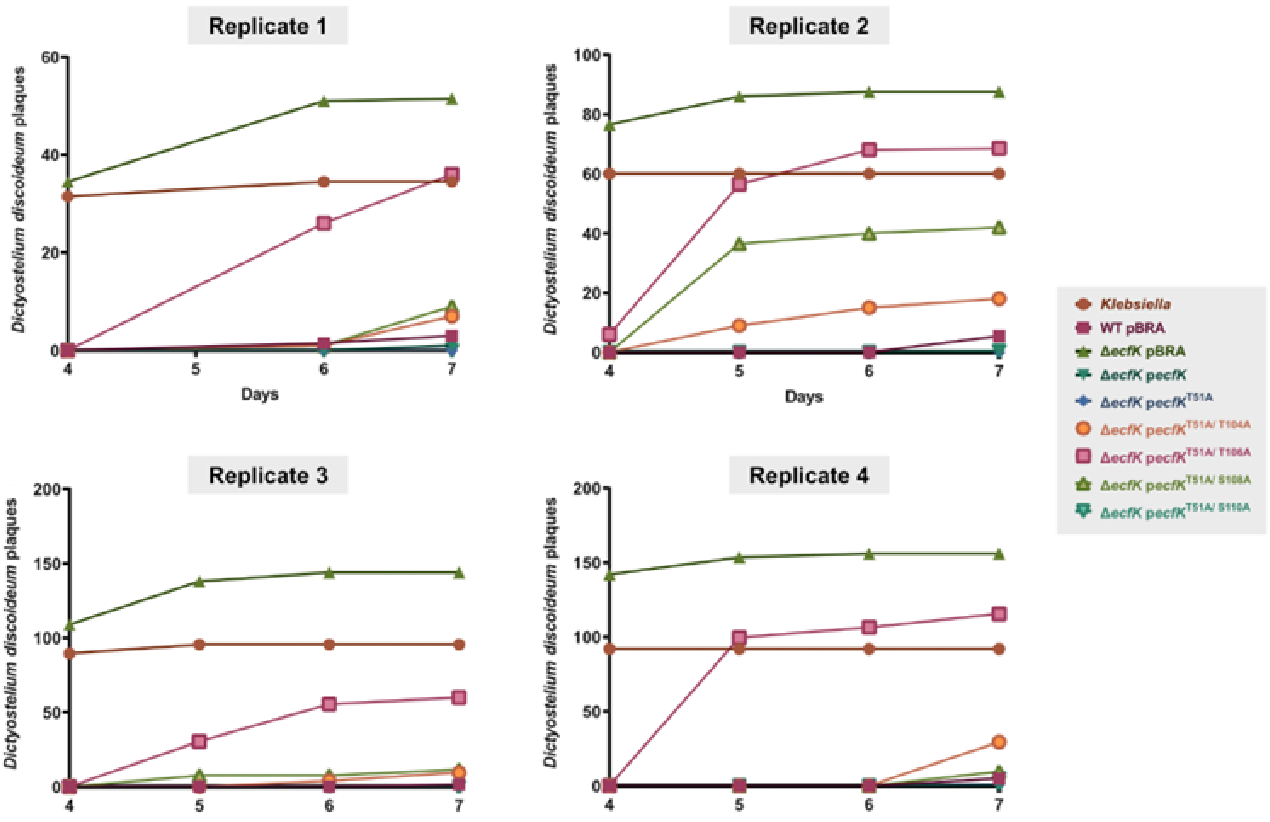

Figure S8: Results of individual plaque assay experiments, which were combined and displayed as relative values in Figure 5D. *X. citri* strains carrying plasmids expressing mutated versions of *ecfK* were mixed with *D. discoideum* amoeba cells and applied onto N agar plates. The total number of amoeba plaques scored on each day (y-axis) are indicated for each strain (color legend on the right grey rectangle). Note that plaques formed onto the lawn of strain carrying the *ecfK*$^{T51A/T106A}$ plasmid appear with a delay compared to the positive control for plaque formation (T6SS mutant, Δ*ecfK* pBRA). A *Klebsiella pneumoniae* strain used as food source for *D. discoideum* was included as a positive control for edibility.

FIGURE S9

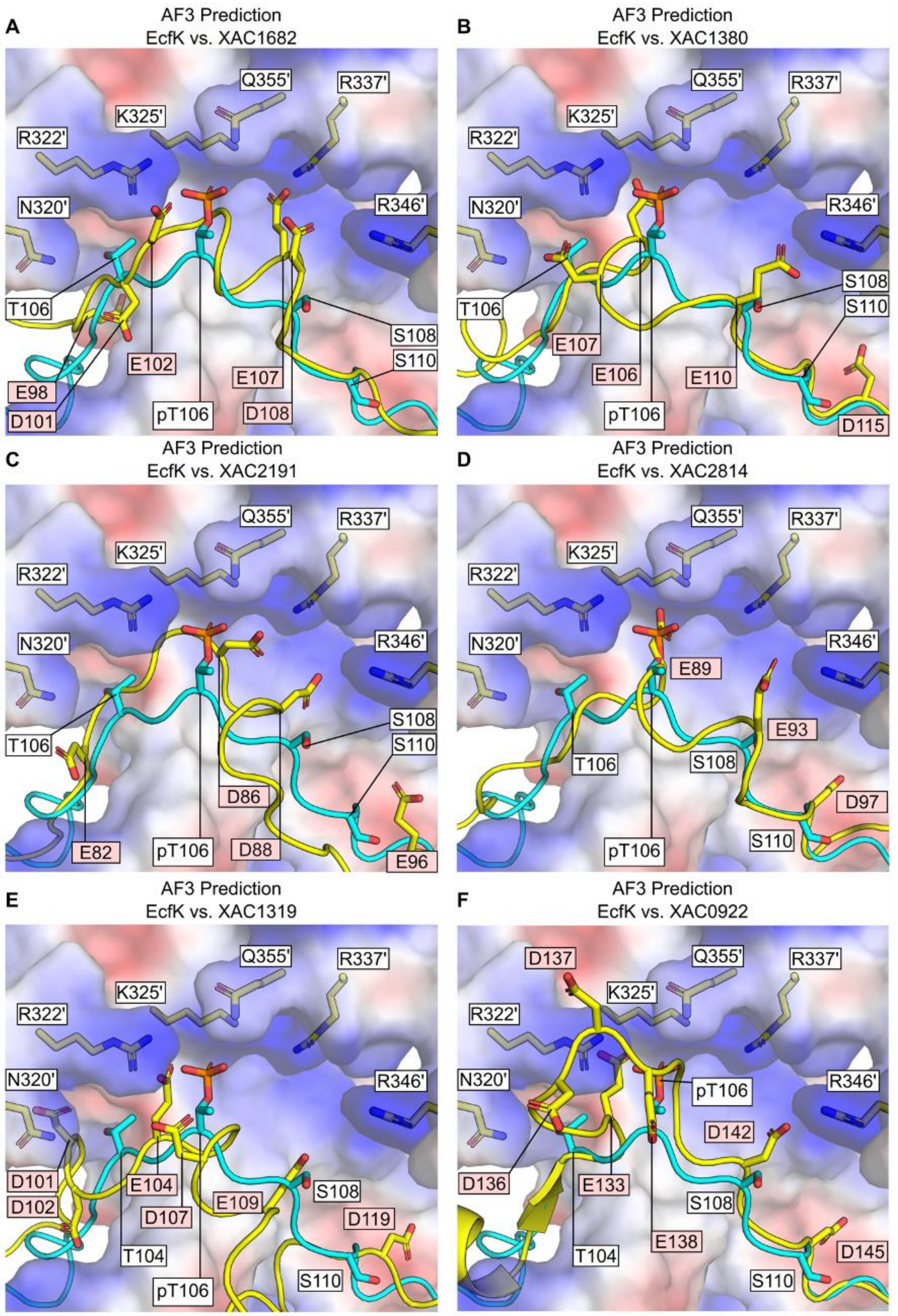

Figure S9: **Detailed views of potential contacts and charge complementarity between *X. citri* RpoC and the selected ECF sigma factors.** Structures were predicted using AlphaFold3. RpoC protein surface is represented as an electrostatic potential map - blue indicates positive charge, red indicates negative charge. Sigma factors are

shown as cartoon models with residues likely important for protein-protein interactions shown as sticks. EcfK is shown with carbon atoms in cyan. Other *X. citri* sigma factors of the ECF family are shown with carbon atoms in yellow. Sigma factors used to build individual complexes were: EcfK (**A-F**; KEGG Entry: XAC4128; UNIPROT ID: A0AAI7ZIW4); **(A)** XAC1682 (KEGG Entry: XAC1682; UNIPROT ID: A0AAI7ZET4); **(B)** RpoE (KEGG Entry: XAC1380 UNIPROT ID: A0AAI8ER26); **(C)** XAC2191 (KEGG Entry: XAC2191; UNIPROT ID: A0AAI7ZFJ4); **(D)** RfaY (KEGG Entry: XAC2814; UNIPROT ID: A0AAI7ZGL0); **(E)** AlgU (KEGG Entry: XAC1319; UNIPROT ID: A0AAI8ES64); **(F)** XAC0922 (KEGG Entry: XAC0922; UNIPROT ID: A0AAI7ZDN4). All images produced using PyMOL Molecular Graphics System, Version 3.0.0.

Figure S10

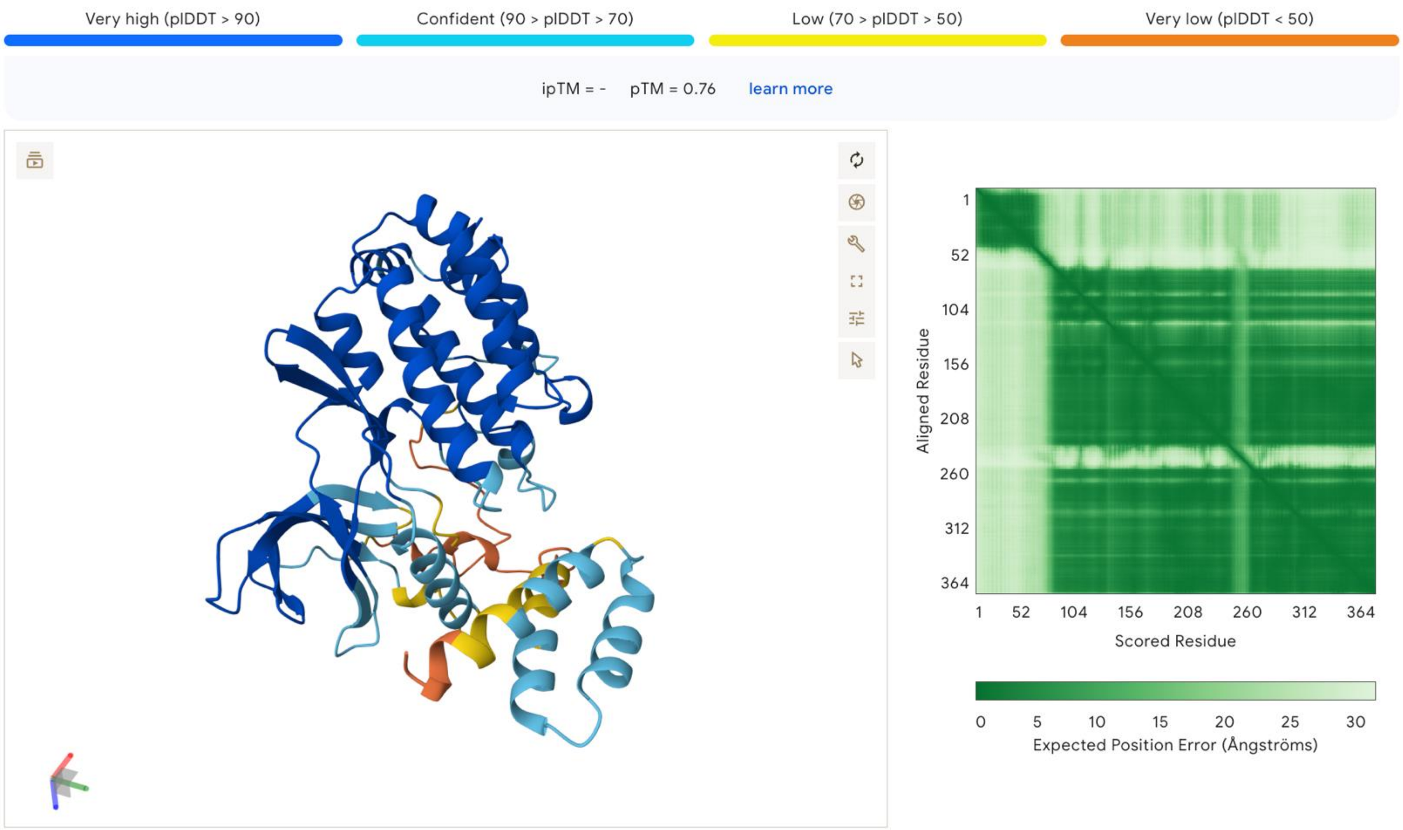

Supplementary Figure S10 - Quality assessment of the AlphaFold3-predicted structure of PknS shown in Figure S3. The plots summarize confidence metrics for the structural model generated using the primary amino acid sequence of PknS. Left panel: Per-residue confidence scores (pLDDT), ranging from 0 to 100. A color bar above the plot indicates confidence levels: blue for very high confidence (pLDDT > 90), cyan for confident (70 < pLDDT ≤ 90), yellow for low confidence (50 < pLDDT ≤ 70), and orange for very low confidence or likely disordered regions (pLDDT ≤ 50). Right panel: Predicted Aligned Error (PAE) heatmap showing the expected positional error (in Å) between residue pairs when aligned on the true structure. Lower values (dark green) indicate high confidence in the relative positioning of residues, while higher values (dark to light green) reflect increased uncertainty, often associated with inter-domain flexibility or disorder. Images for pLDDT and PAE plots were generated using the AlphaFold Server (https://alphafoldserver.com/).

Figure S11

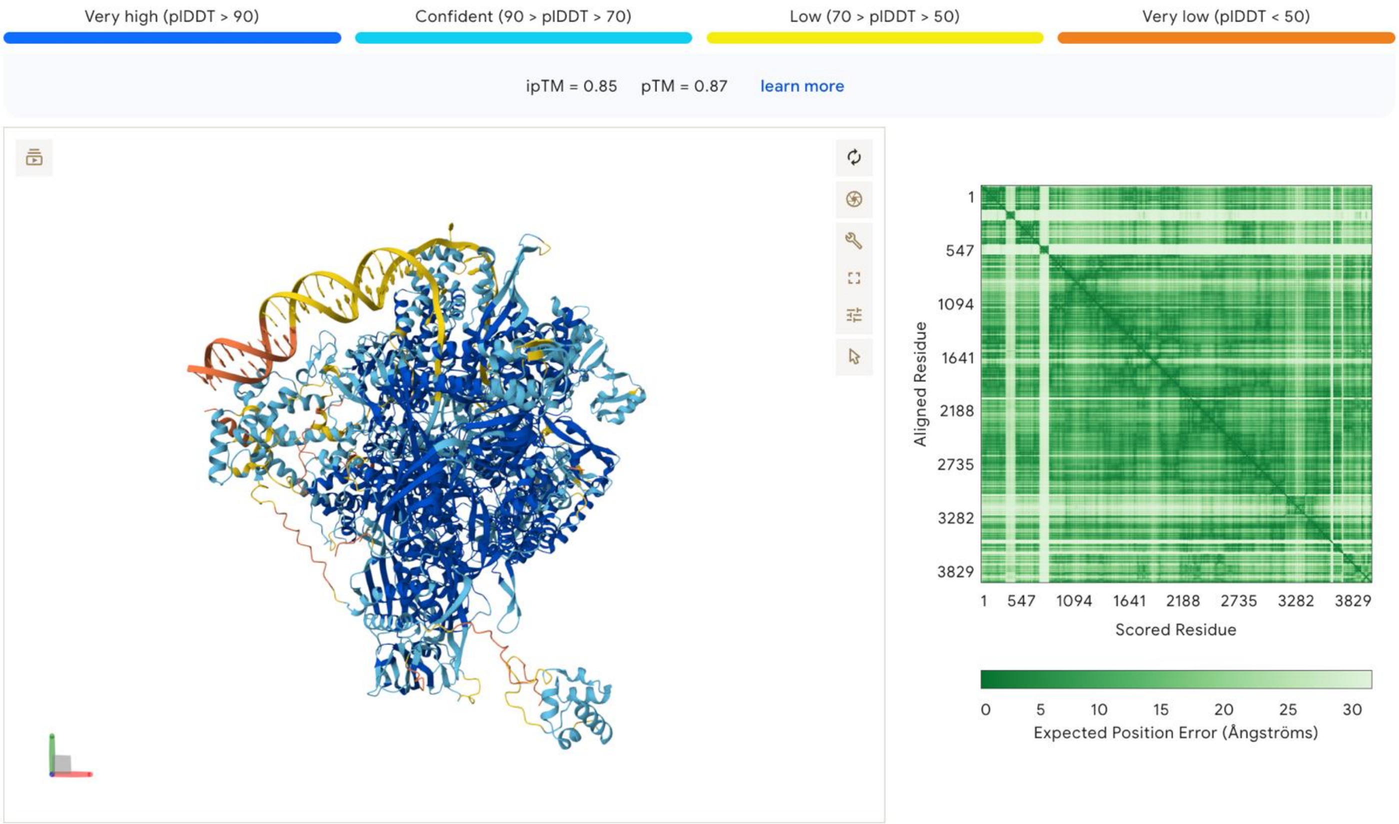

Supplementary Figure S11 - Quality assessment of the AlphaFold3-predicted structure of Holo-RNA Polymerase complexes for *X. citri pv. citri* 306 bound to sigma factor EcfK (XAC4128). The plots summarize confidence metrics for the structural model. Left panel: Per-residue confidence scores (pLDDT), ranging from 0 to 100. A color bar above the plot indicates confidence levels: blue for very high confidence (pLDDT > 90), cyan for confident (70 < pLDDT ≤ 90), yellow for low confidence (50 < pLDDT ≤ 70), and orange for very low confidence or likely disordered regions (pLDDT ≤ 50). Right panel: Predicted Aligned Error (PAE) heatmap showing the expected positional error (in Å) between residue pairs when aligned on the true structure. Lower values (dark green) indicate high confidence in the relative positioning of residues, while higher values (dark to light green) reflect increased uncertainty, often associated with inter-domain flexibility or disorder. Images for pLDDT and PAE plots were generated using the AlphaFold Server (https://alphafoldserver.com/).

Figure S12

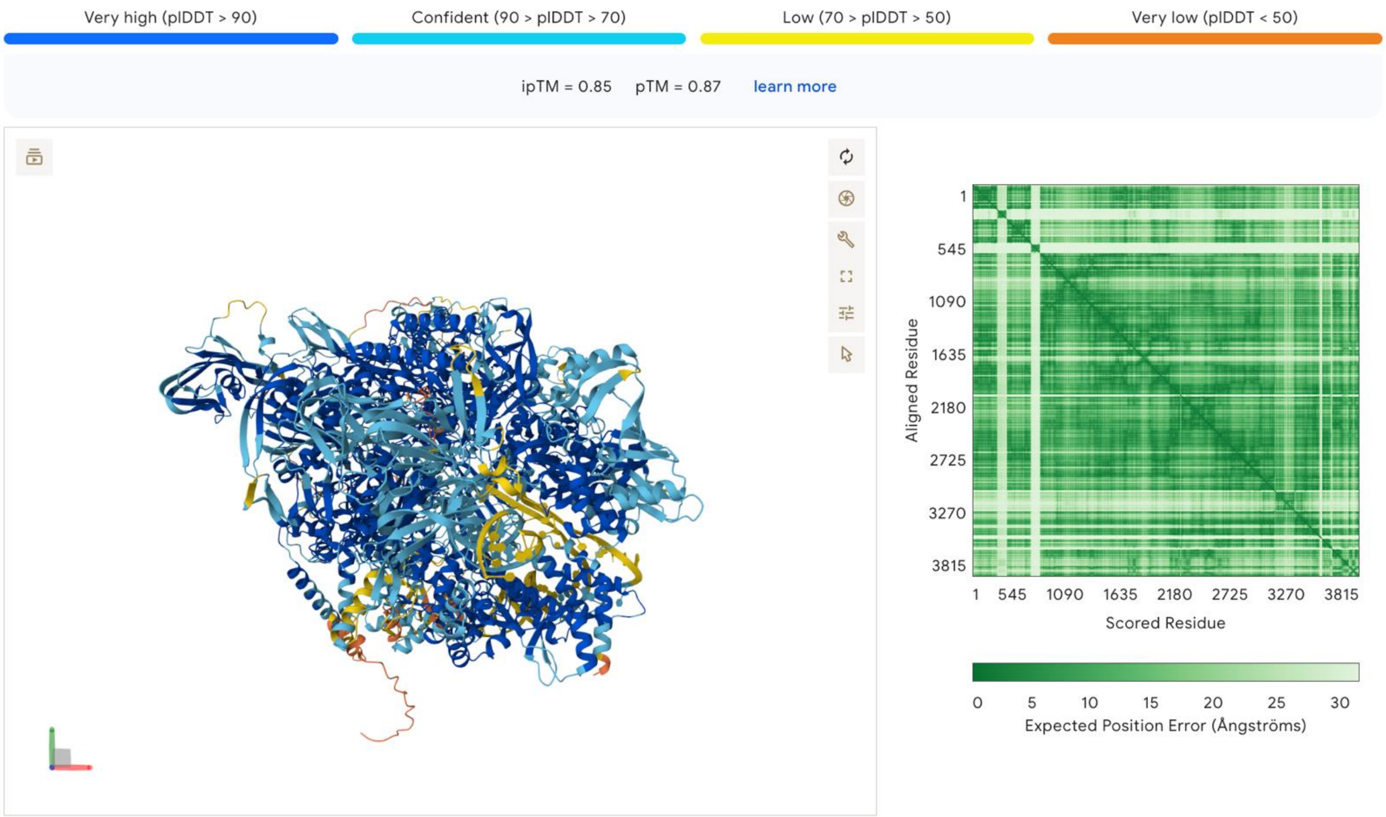

Supplementary Figure S12 - Quality assessment of the AlphaFold3-predicted structure of Holo-RNA Polymerase complexes for *X. citri pv. citri* 306 bound to sigma factor RfaY (XAC2814). The plots summarize confidence metrics for the structural model. Left panel: Per-residue confidence scores (pLDDT), ranging from 0 to 100. A color bar above the plot indicates confidence levels: blue for very high confidence (pLDDT > 90), cyan for confident (70 < pLDDT ≤ 90), yellow for low confidence (50 < pLDDT ≤ 70), and orange for very low confidence or likely disordered regions (pLDDT ≤ 50). Right panel: Predicted Aligned Error (PAE) heatmap showing the expected positional error (in Å) between residue pairs when aligned on the true structure. Lower values (dark green) indicate high confidence in the relative positioning of residues, while higher values (dark to light green) reflect increased uncertainty, often associated with inter-domain flexibility or disorder. Images for pLDDT and PAE plots were generated using the AlphaFold Server (https://alphafoldserver.com/).

Figure S13

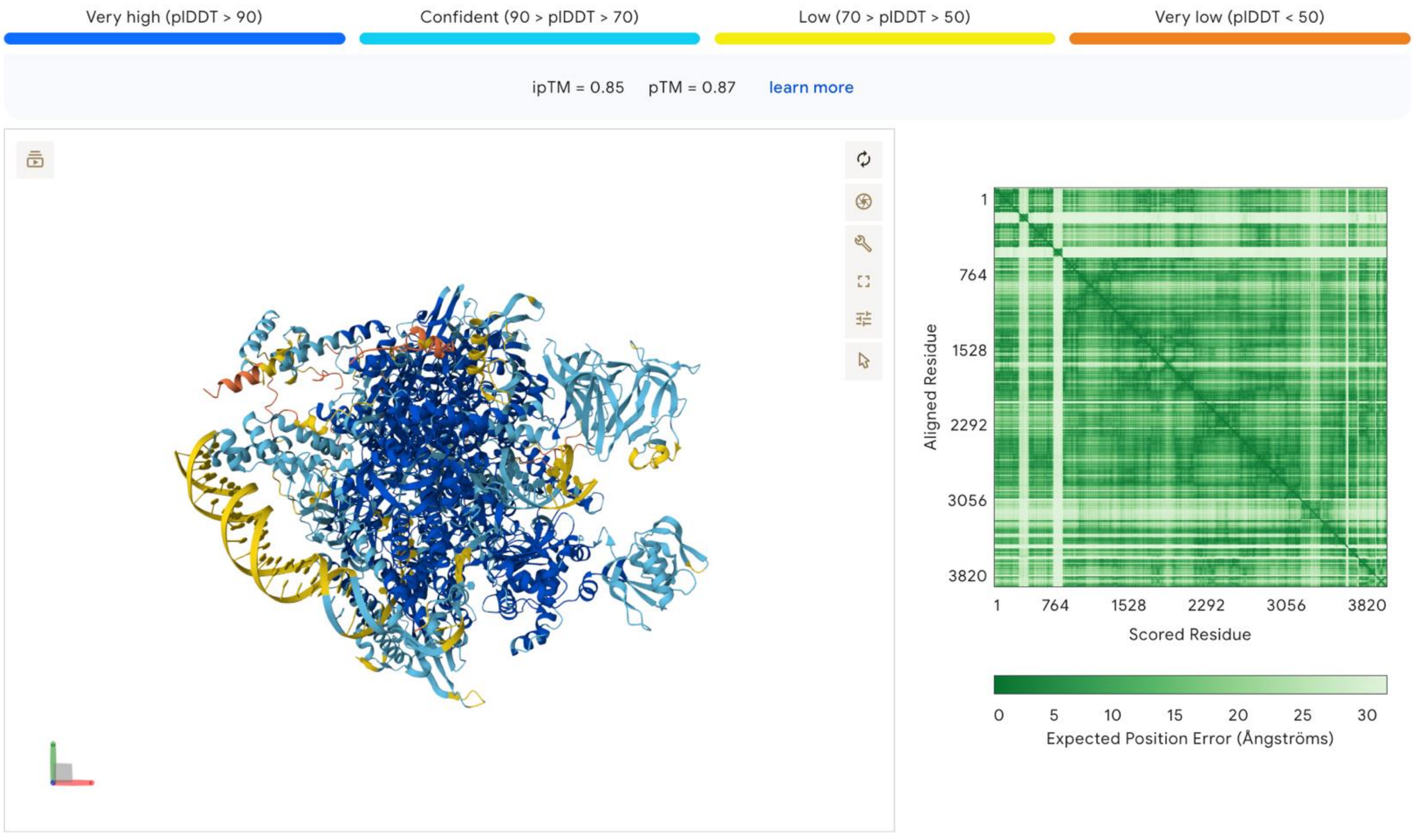

Supplementary Figure S13 - Quality assessment of the AlphaFold3-predicted structure of Holo-RNA Polymerase complexes for *X. citri pv. citri* 306 bound to sigma factor PrtI (XAC3989). The plots summarize confidence metrics for the structural model. Left panel: Per-residue confidence scores (pLDDT), ranging from 0 to 100. A color bar above the plot indicates confidence levels: blue for very high confidence (pLDDT > 90), cyan for confident (70 < pLDDT ≤ 90), yellow for low confidence (50 < pLDDT ≤ 70), and orange for very low confidence or likely disordered regions (pLDDT ≤ 50). Right panel: Predicted Aligned Error (PAE) heatmap showing the expected positional error (in Å) between residue pairs when aligned on the true structure. Lower values (dark green) indicate high confidence in the relative positioning of residues, while higher values (dark to light green) reflect increased uncertainty, often associated with inter-domain flexibility or disorder. Images for pLDDT and PAE plots were generated using the AlphaFold Server (https://alphafoldserver.com/).

Figure S14

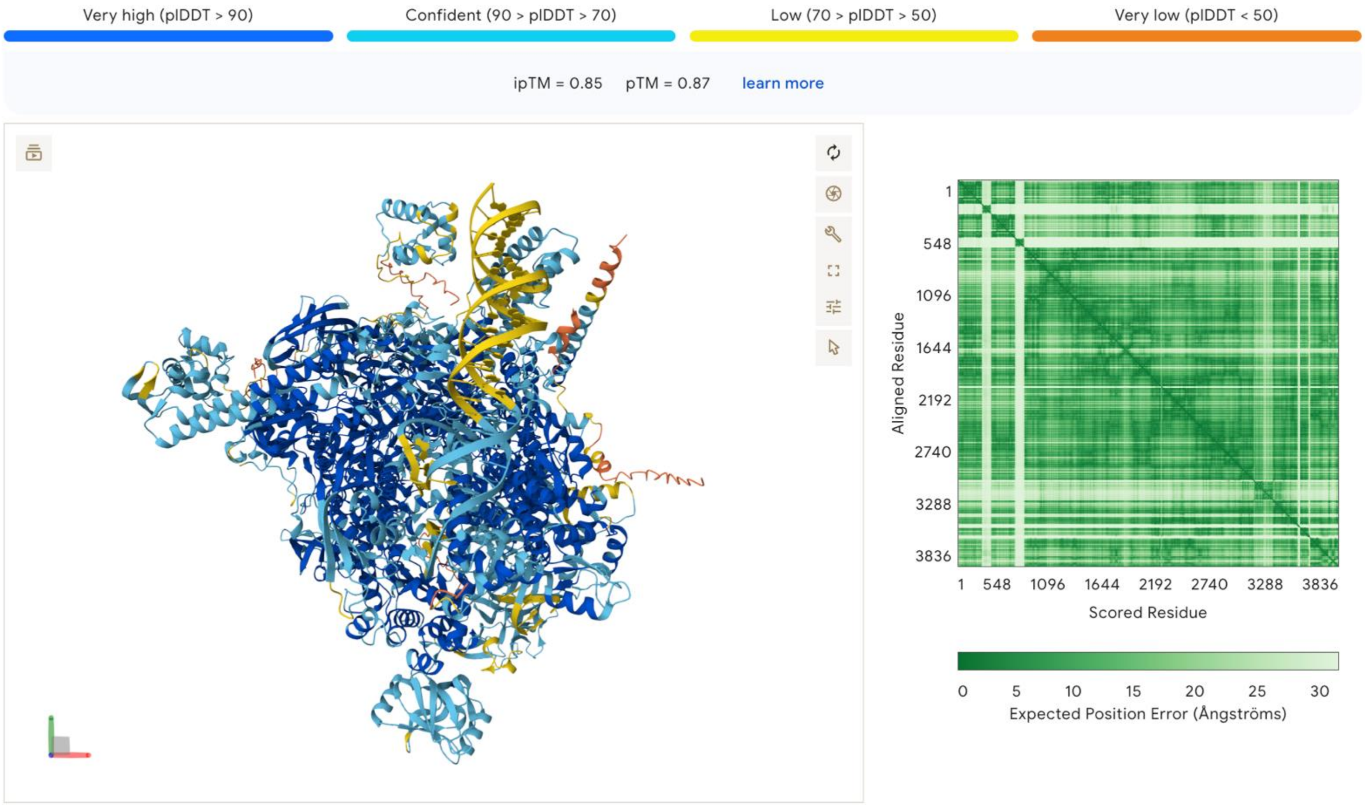

Supplementary Figure S14 - Quality assessment of the AlphaFold3-predicted structure of Holo-RNA Polymerase complexes for *X. citri pv. citri* 306 bound to sigma factor RpoE (XAC1380). The plots summarize confidence metrics for the structural model. Left panel: Per-residue confidence scores (pLDDT), ranging from 0 to 100. A color bar above the plot indicates confidence levels: blue for very high confidence (pLDDT > 90), cyan for confident (70 < pLDDT ≤ 90), yellow for low confidence (50 < pLDDT ≤ 70), and orange for very low confidence or likely disordered regions (pLDDT ≤ 50). Right panel: Predicted Aligned Error (PAE) heatmap showing the expected positional error (in Å) between residue pairs when aligned on the true structure. Lower values (dark green) indicate high confidence in the relative positioning of residues, while higher values (dark to light green) reflect increased uncertainty, often associated with inter-domain flexibility or disorder. Images for pLDDT and PAE plots were generated using the AlphaFold Server (https://alphafoldserver.com/).

Figure S15

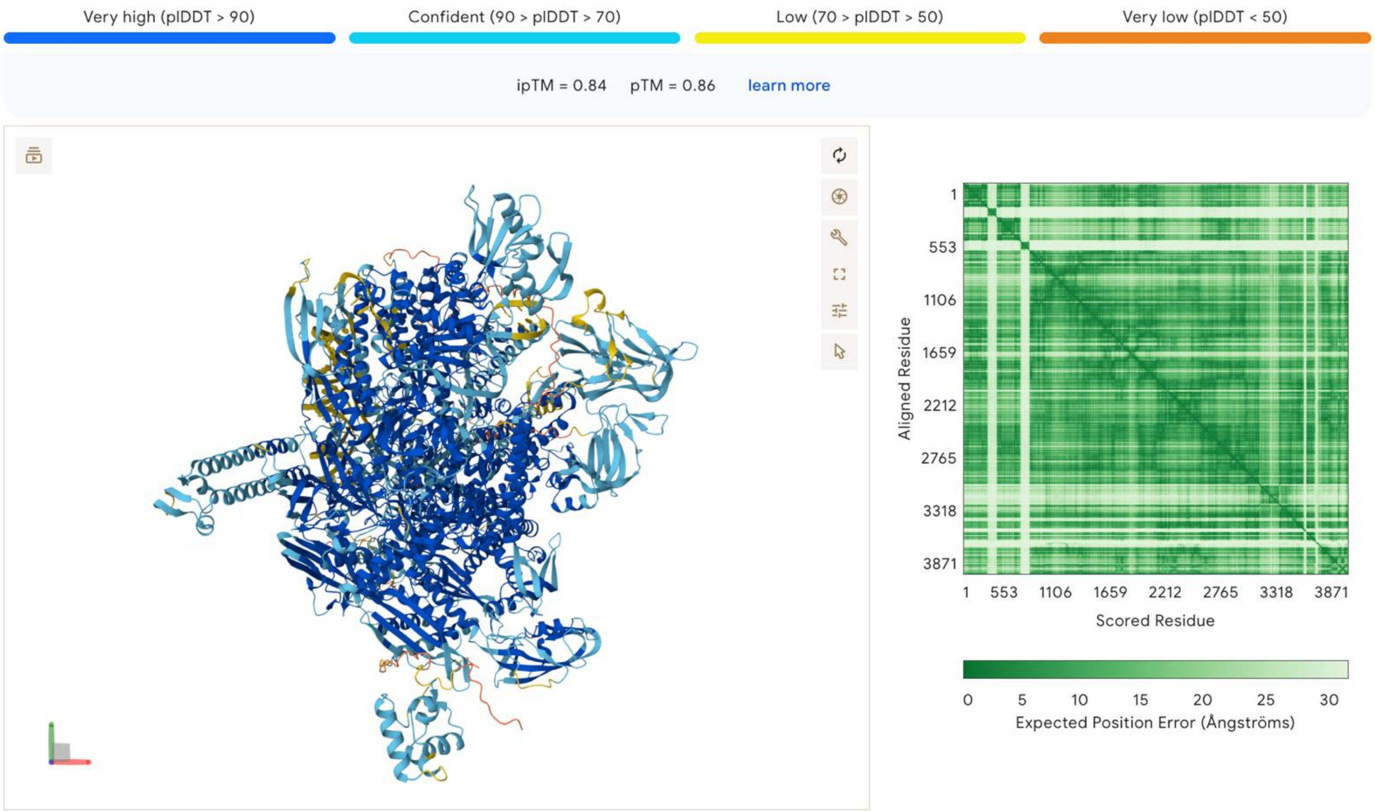

Supplementary Figure S15 - Quality assessment of the AlphaFold3-predicted structure of Holo-RNA Polymerase complexes for *X. citri pv. citri* 306 bound to sigma factor XAC0922. The plots summarize confidence metrics for the structural model. Left panel: Per-residue confidence scores (pLDDT), ranging from 0 to 100. A color bar above the plot indicates confidence levels: blue for very high confidence (pLDDT > 90), cyan for confident (70 < pLDDT ≤ 90), yellow for low confidence (50 < pLDDT ≤ 70), and orange for very low confidence or likely disordered regions (pLDDT ≤ 50). Right panel: Predicted Aligned Error (PAE) heatmap showing the expected positional error (in Å) between residue pairs when aligned on the true structure. Lower values (dark green) indicate high confidence in the relative positioning of residues, while higher values (dark to light green) reflect increased uncertainty, often associated with inter-domain flexibility or disorder. Images for pLDDT and PAE plots were generated using the AlphaFold Server (https://alphafoldserver.com/).

Figure S16

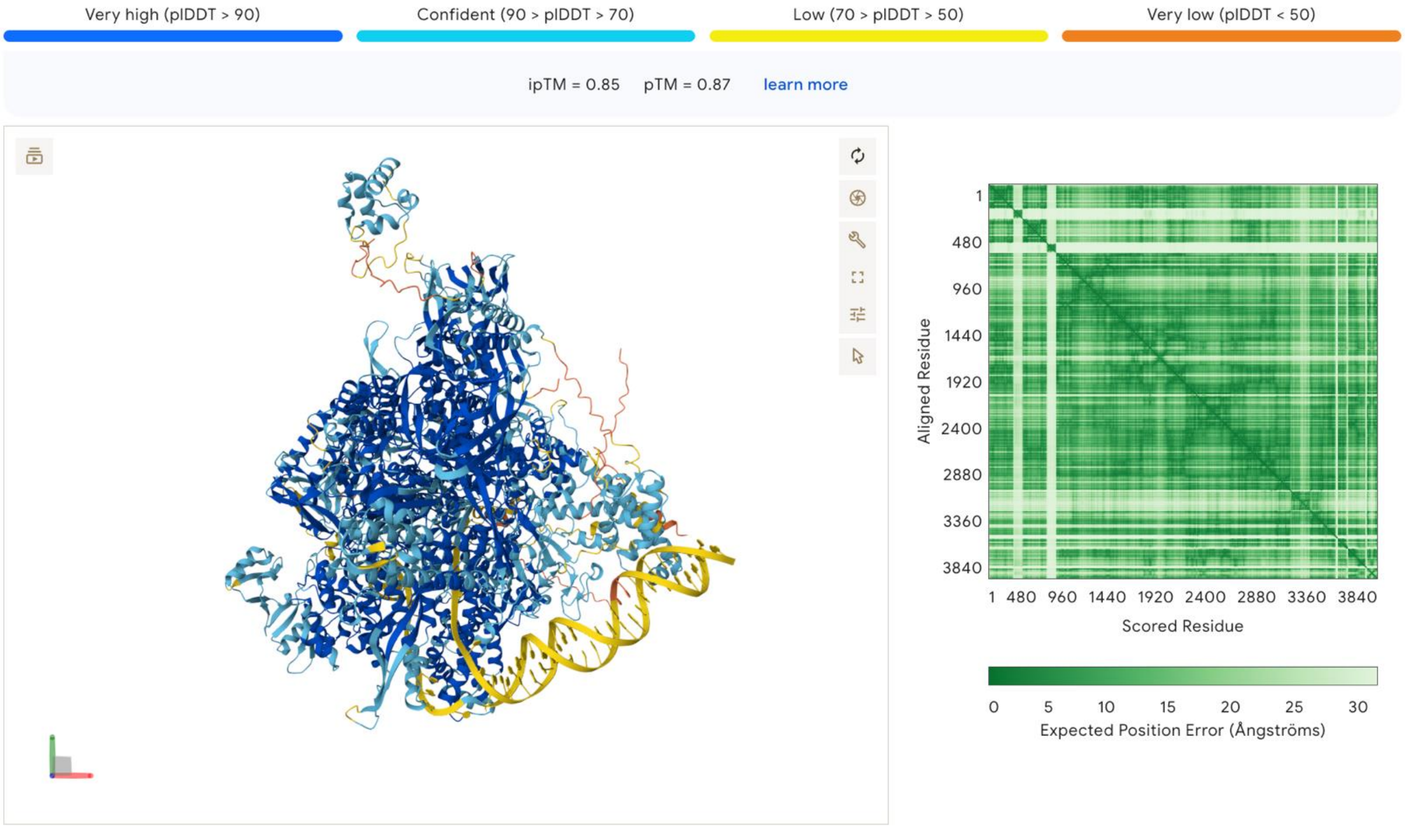

Supplementary Figure S16 - Quality assessment of the AlphaFold3-predicted structure of Holo-RNA Polymerase complexes for *X. citri pv. citri* 306 bound to sigma factor XAC2191. The plots summarize confidence metrics for the structural model. Left panel: Per-residue confidence scores (pLDDT), ranging from 0 to 100. A color bar above the plot indicates confidence levels: blue for very high confidence (pLDDT > 90), cyan for confident (70 < pLDDT ≤ 90), yellow for low confidence (50 < pLDDT ≤ 70), and orange for very low confidence or likely disordered regions (pLDDT ≤ 50). Right panel: Predicted Aligned Error (PAE) heatmap showing the expected positional error (in Å) between residue pairs when aligned on the true structure. Lower values (dark green) indicate high confidence in the relative positioning of residues, while higher values (dark to light green) reflect increased uncertainty, often associated with inter-domain flexibility or disorder. Images for pLDDT and PAE plots were generated using the AlphaFold Server (https://alphafoldserver.com/).

Figure S17

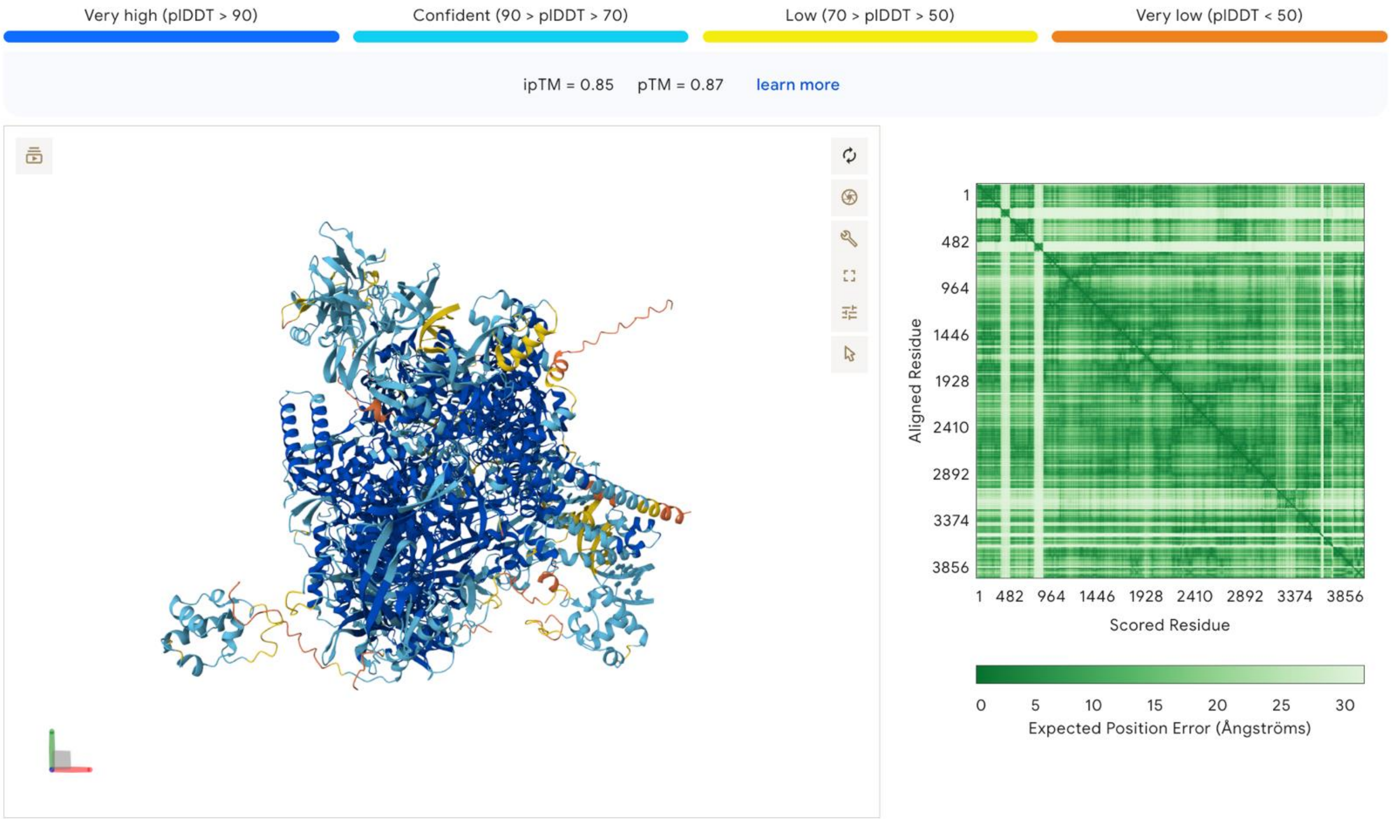

Supplementary Figure S17 - Quality assessment of the AlphaFold3-predicted structure of Holo-RNA Polymerase complexes for *X. citri pv. citri* 306 bound to sigma factor AlgU (XAC1319). The plots summarize confidence metrics for the structural model. Left panel: Per-residue confidence scores (pLDDT), ranging from 0 to 100. A color bar above the plot indicates confidence levels: blue for very high confidence (pLDDT > 90), cyan for confident (70 < pLDDT ≤ 90), yellow for low confidence (50 < pLDDT ≤ 70), and orange for very low confidence or likely disordered regions (pLDDT ≤ 50). Right panel: Predicted Aligned Error (PAE) heatmap showing the expected positional error (in Å) between residue pairs when aligned on the true structure. Lower values (dark green) indicate high confidence in the relative positioning of residues, while higher values (dark to light green) reflect increased uncertainty, often associated with inter-domain flexibility or disorder. Images for pLDDT and PAE plots were generated using the AlphaFold Server (https://alphafoldserver.com/).

Figure S18

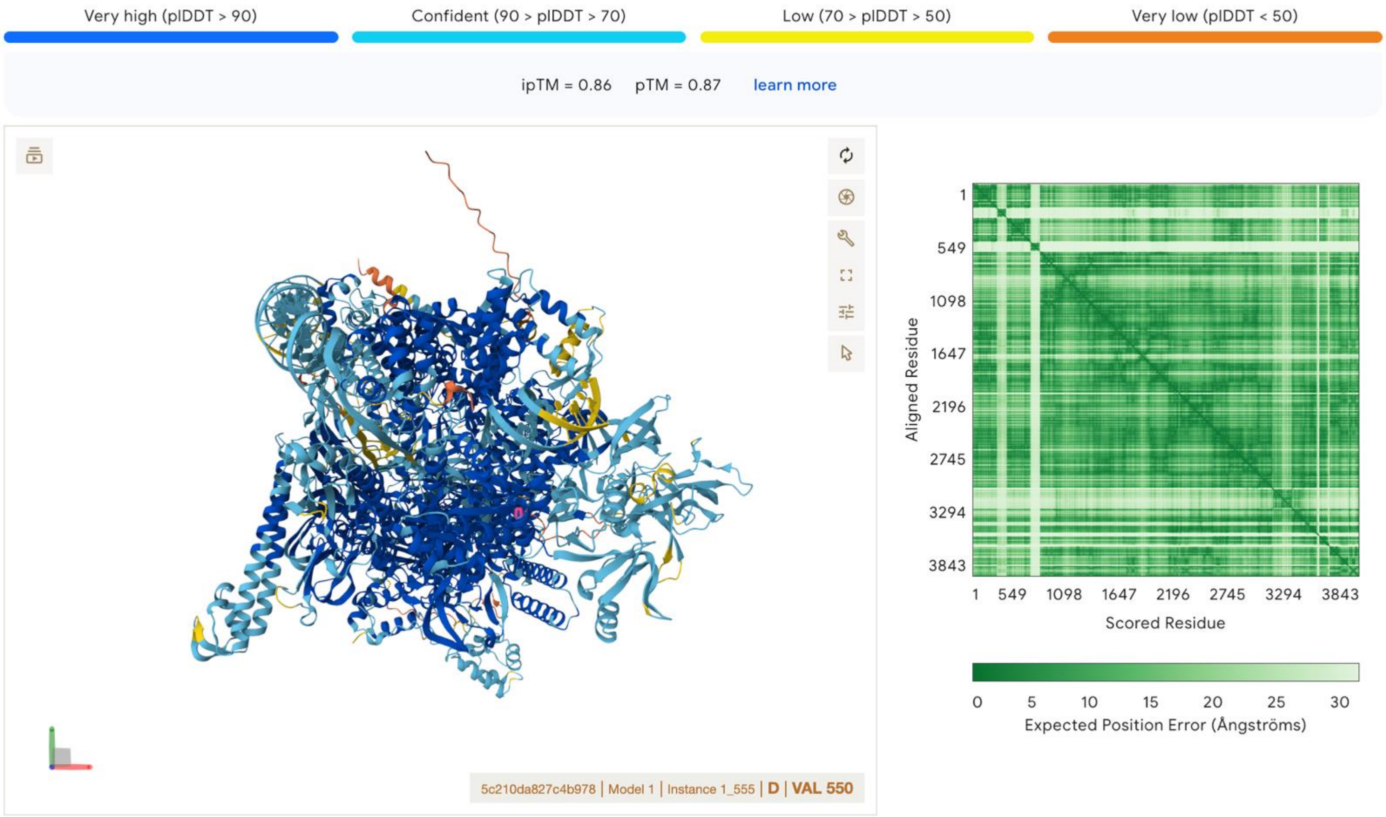

Supplementary Figure S18 - Quality assessment of the AlphaFold3-predicted structure of Holo-RNA Polymerase complexes for *X. citri pv. citri* 306 bound to sigma factor XAC1682. The plots summarize confidence metrics for the structural model. Left panel: Per-residue confidence scores (pLDDT), ranging from 0 to 100. A color bar above the plot indicates confidence levels: blue for very high confidence (pLDDT > 90), cyan for confident (70 < pLDDT ≤ 90), yellow for low confidence (50 < pLDDT ≤ 70), and orange for very low confidence or likely disordered regions (pLDDT ≤ 50). Right panel: Predicted Aligned Error (PAE) heatmap showing the expected positional error (in Å) between residue pairs when aligned on the true structure. Lower values (dark green) indicate high confidence in the relative positioning of residues, while higher values (dark to light green) reflect increased uncertainty, often associated with inter-domain flexibility or disorder. Images for pLDDT and PAE plots were generated using the AlphaFold Server (https://alphafoldserver.com/).

Figure S19

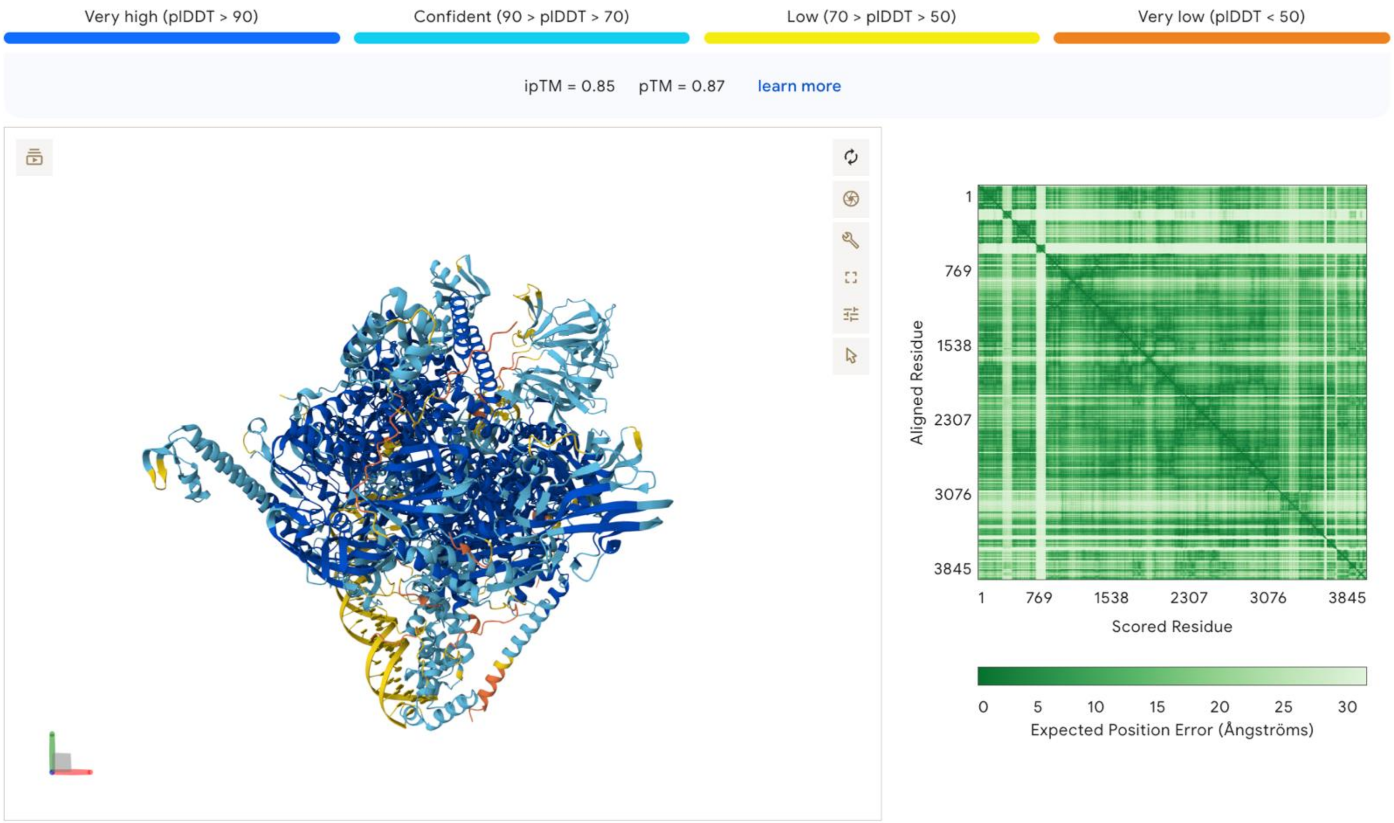

Supplementary Figure S19 - Quality assessment of the AlphaFold3-predicted structure of Holo-RNA Polymerase complexes for *V. parahaemolyticus* RIMD 2210633. The plots summarize confidence metrics for the structural model. Left panel: Per-residue confidence scores (pLDDT), ranging from 0 to 100. A color bar above the plot indicates confidence levels: blue for very high confidence (pLDDT > 90), cyan for confident (70 < pLDDT ≤ 90), yellow for low confidence (50 < pLDDT ≤ 70), and orange for very low confidence or likely disordered regions (pLDDT ≤ 50). Right panel: Predicted Aligned Error (PAE) heatmap showing the expected positional error (in Å) between residue pairs when aligned on the true structure. Lower values (dark green) indicate high confidence in the relative positioning of residues, while higher values (dark to light green) reflect increased uncertainty, often associated with inter-domain flexibility or disorder. Images for pLDDT and PAE plots were generated using the AlphaFold Server (https://alphafoldserver.com/).

Table S8. Strains used in this study.

Strain	Description	Source/reference
	***Escherichia coli* strains**	
DH5α	F– φ80*lacZ*ΔM15 Δ(*lacZYA-argF*)U169 *recA1 endA1 hsdR*17(rK–, mK+) *phoA supE44* λ–*thi*-1 *gyrA*96 *relA1*. Cloning strain.	[55]
Mach1™-T1^R	F^- ϕ80(*lacZ*)ΔM15 Δ*lacX*74 *hsdR*(r_K^- m_K^+) Δ*recA*1398 *endA1 tonA*. Resistant to bacteriophage T1 and T5. Cloning strain.	ThermoFisher Scientific (C862003)
BL21(DE3)-R3-pRARE2	Phage resistant derivative of BL21(DE3) (F^-*ompT hsdS*$_B$($r_B^-m_B^-$) *gal dam* (DE3)), carrying pRARE2 plasmid for expression of rare-codon tRNA genes (Cm^r). Expression strain.	[58,61]
BL21(DE3)-R3-lambdaPpase	Phage resistant derivative of BL21(DE3) (F-ompT hsdSB(rB-mB-) gal dam (DE3)) with plasmid pACYC-LIC expressing Lambda phosphatase. Expression strain. Cm^r.	[58,61]
	***Xanthomonas citri* strains**	
X. citri 306	*Xanthomonas citri* pv. citri, strain 306, IBSBF1594, Wild type. Ap^r.	[56]
Δ*ecfK*	*ecfK* (XAC4128) in-frame deletion strain, Ap^r.	[14]
Δ*ecfK* pBRA	Δ*ecfK* strain carrying the empty vector pBRA, Ap^r, Sp^r, Str^r.	[14]
Δ*ecfK ecfK*	Δ*ecfK* strain carrying pBRA with the wild-type copy of *ecfK,* Ap^r, Sp^r, Str^r.	[14]
Δ*ecfK ecfK*^{T51A}	Δ*ecfK* strain carrying pBRA containing *ecfK* gene with T51A substitution, Ap^r, Sp^r, Str^r.	[14]
Δ*ecfK ecfK*$^{T51A/T104A}$	Δ*ecfK* strain carrying pBRA containing *ecfK* gene with T51A and T104A substitutions, Ap^r, Sp^r, Str^r.	This work
Δ*ecfK ecfK*$^{T51A/T106A}$	Δ*ecfK* strain carrying pBRA containing *ecfK* gene with T51A and T106A substitutions, Ap^r, Sp^r, Str^r.	This work
Δ*ecfK ecfK*$^{T51A/S108A}$	Δ*ecfK* strain carrying pBRA containing *ecfK* gene with T51A and S108A substitutions, Ap^r, Sp^r, Str^r.	This work
Δ*ecfK ecfK*$^{T51A/S110A}$	Δ*ecfK* strain carrying pBRA containing *ecfK* gene with T51A and S110A substitutions, Ap^r, Sp^r, Str^r.	This work
Δ*pknS*	*pknS* (XAC4127) in-frame deletion strain, Ap^r.	[14]

Δ*pknS FLAG-pknS*$_{(FL)}$	Δ*pknS* strain carrying pBRA containing full-length *pknS* open reading frame fused to the 1xFLAG epitope sequence (coding for DYKDDDDK). Ap^r, Sp^r, Str^r.	This work.
Δ*pknS FLAG-pknS*$_{(1\text{-}364)}$	Δ*pknS* strain carrying pBRA with *pknS* open reading frame encompassing residues 1-364 (kinase domain) fused to the 1xFLAG epitope sequence (coding for DYKDDDDK). Ap^r, Sp^r, Str^r.	This work.
Δ*pknS FLAG-pknS*$_{(1\text{-}449)}$	Δ*pknS* strain carrying pBRA with *pknS* open reading frame encompassing residues 1-449 (membrane-tethered kinase) fused to the 1xFLAG epitope sequence (coding for DYKDDDDK). Ap^r, Sp^r, Str^r.	This work.
	***Dictyostelium discoideum* strain**	
AX2	*Dictyostelium discoideum*, strain Ax2(Ka)	[57]

Table S9. Plasmids used in this study.

Plasmid	Description	Source/reference
pBRA	Contains *araC* gene and the arabinose-inducible promoter derived from pBAD24, *mob* and pBBRMCS1 *ori*. Str^rSp^r.	Marroquim M, Andrade M e Farah, CS, unpublished.
pNIC28-Bsa4	pET28a derivative. Contains sequence corresponding to a 22 amino acid N-terminal peptide, which includes His_6 tag and TEV protease cleavage site. Suitable for ligase-independent cloning (LIC), includes the *sacB* gene for negative selection. T7-lacO promoter. Km^R.	[58], Structural Genomics Consortium (SGC)
pBRA-*ecfK*^{T51A}	pBRA containing the ecfK with T51A mutation. Used as template for generation of *ecfK* versions with double mutations. Str^rSp^r.	[14]

Table S10. Constructs for heterologous expression in *E. coli*.

Constructs	Description	Reference
pNIC-His_6EcfK	pNIC28-Bsa4 with *ecfK* open reading frame. Generated by LIC cloning. Expression of His_6-EcfK. Km^R.	[14]
pNIC-His_6PknS$_{1\text{–}364}$	Expression vector with *pknS* open reading encompassing residues 1-364 (kinase domain). Generated by LIC cloning. Expression of His_6-PknS$_{(1\text{-}364)}$.	This work.
pNIC-His_6PknS$_{1\text{–}364}$M$_{164}$A	Expression vector containing *pknS* open reading encompassing residues 1-364 (kinase domain) with mutation M164A. Generated by site-directed mutagenesis using pNIC-His_6PknS$_{1\text{–}364}$ as template. Expression of His_6-PknS$_{1\text{–}364}$M$_{164}$A.	This work.

Table S11. Oligonucleotides used in this study.

**Name**	**Sequence (5' > 3')**	**Description**
	**Cloning**	
PnkS_LIC_for	TAC TTC CAA TCC ATG AGC CTG GCG GCC ACC	For cloning pNIC-His$_6$PknS$_{1–364}$. LIC cloning site is underlined.
PknS_LIC_rev	TAT CCA CCT TTA CTG TTA ACC GTC CAG CCA GCG ATG	For cloning pNIC-His$_6$PknS$_{1–364}$. LIC cloning site is underlined.
pLIC-F	TGTGAGCGGATAACAATTCC	DNA sequencing and colony PCR and colony PCR screening of pNIC clones
pLIC-R	AGCAGCCAACTCAGCTTCC	DNA sequencing and colony PCR screening of pNIC clones
flag-pknS-F1	AAAGCTAGCAGGAGGAATTCACC ATGGACTACAAGGATGACGATGA CAAGGTGAGCCTGGCGGCCAC	Forward primer with NheI site and flag epitope sequence. For cloning of *FLAG-pknS* versions in the pBRA vector.
pknS-FL-R	AAAGTCGACCAATTCGTCTCAGC GCTG	Reverse primer with SalI site. For cloning *FLAG-pknS*$_{(FL)}$ in the pBRA vector.
pknS-kin-R1	AATGTCGACTCAACCGTCCAGCC AGCGATGCA	Reverse primer with SalI site. For cloning *FLAG-pknS*$_{(1-364)}$ in the pBRA vector.
pknS-TMD-R2	AATGTCGACTCACAGCATCAGGG TCTTGTCCA	Reverse primer with SalI site. For cloning *FLAG-pknS*$_{(1-449)}$ in the pBRA vector.
	**Site directed-mutagenesis**	
EcfK-T104A_1	GGGCAGGCGGTGGCGCTGACCAT CA	EcfK mutation T104A.
EcfK-T104A_2	TGATGGTCAGCGCCACCGCCTGC CC	EcfK mutation T104A.
ecfK T106A 1	GGC GGT GAC GCT GGC CAT CAG CCT GTC	EcfK mutation T106A.
ecfK T106A 2	GAC AGG CTG ATG GCC AGC GTC ACC GCC	EcfK mutation T106A.
EcfK-S108A_1	GACGCTGACCATCGCCCTGTCCG ACGGC	EcfK mutation S108A.
EcfK-S108A_2	GCCGTCGGACAGGGCGATGGTCA GCGTC	EcfK mutation S108A.
EcfK-S110A_1	GGCGCCGTCGGCCAGGCTGATGG	EcfK mutation S110A.
EcfK-S110A_2	CCATCAGCCTGGCCGACGGCGCC	EcfK mutation S110A.
pknS M164A_P1	CCGCGCAGGTATTCCGCCACCAG GTACGGCTG	PknS mutation M164A.
pknS M164A_P2	CAGCCGTACCTGGTGGCGGAATA CCTGCGCGG	PknS mutation M164A.
